# Stereoselective syntheses of 3-aminocyclooctanetriols and halocyclooctanetriols

**DOI:** 10.3762/bjoc.17.59

**Published:** 2021-03-11

**Authors:** Emine Salamci, Yunus Zozik

**Affiliations:** 1 Department of Chemistry, Faculty of Sciences, Atatürk University, 25240 Erzurum, Turkey

**Keywords:** aminocyclitols, aminocyclooctanetriol, chlorocyclooctanetriol, cyclic sulfate, cyclitols

## Abstract

The efficient synthesis of two new stereoisomeric 3-aminocyclooctanetriols and their new halocyclitol derivatives starting from *cis,cis*-1,3-cyclooctadiene are reported. Reduction of cyclooctene endoperoxide, obtained by photooxygenation of *cis,cis*-1,3-cyclooctadiene, with zinc yielded a cyclooctene diol followed by acetylation of the hydroxy group, which gave dioldiacetate by OsO_4_/NMO oxidation. The cyclooctane dioldiacetate prepared was converted to the corresponding cyclic sulfate via the formation of a cyclic sulfite in the presence of catalytic RuO_4_. The reaction of this cyclic sulfate with a nucleophilic azide followed by the reduction of the azide group provided the target, 3-aminocyclooctanetriol. The second key compound, bromotriol, was prepared by epoxidation of the cyclooctenediol with *m*-chloroperbenzoic acid followed by hydrolysis with HBr(g) in methanol. Treatment of bromotriol with NaN_3_ and the reduction of the azide group yielded the other desired 3-aminocyclooctanetriol. Hydrolysis of the epoxides with HCl(g) in methanol gave stereospecifically new chlorocyclooctanetriols.

## Introduction

**Figure 1:**
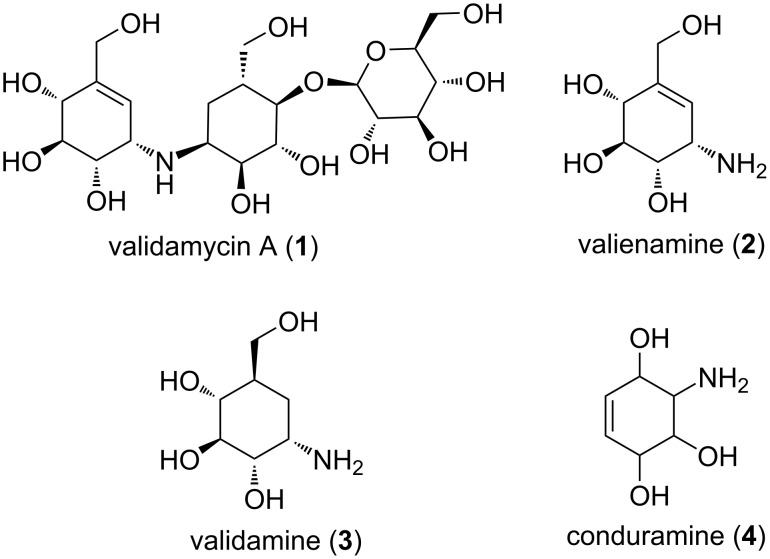
Structures of some important aminocyclitols.

The synthesis of aminocyclitols has attracted attention because they contain substructures of many biologically active natural products [1–3]. They have become important structural components for drug development with a modifying action as inhibitors of glycosidases [4–10]. Aminocyclitols are amino polyhydroxy cycloalkanes [2] formally derived from cyclitols [11–15], which are polyhydroxylated cycloalkanes, via replacement of one of the hydroxy groups with an amino group. Many aminocyclitols and their derivatives have been found to possess antibiotic properties, such as validamycins (**1**) [16]. Validamycin A (**1**) contains two aminocyclitol units, the one is valienamine (**2**) and the other is validamine (**3**, Figure 1).

One of the most important conduramines **4** is valienamine (**3**) [17], which is found as a building block in several aminoglycoside antibiotics [2]. Furthermore, conduramines **4** and their derivatives are used as both inhibitors of glycosidases and useful intermediates in organic synthesis [18]. Halocyclitols are also cyclitol derivatives, in which one of the hydroxy groups is replaced by a halogen. They have also attracted interest in the last decade because of their biological activities [11,19]. For instance, some brominated quercitol (cyclohexanepentol) derivatives and bromoconduritol-B act as strong inhibitors of α-glycosidases [11,19]. Recent reviews report on the latest synthetic methodologies for aminocyclitols and related compounds [1–3,16].

Many methods have been previously reported for the synthesis of aminocyclitols containing five- and six-membered rings, along with their diverse biological activities [1–3,16–26]. However, only a limited number of synthetic methods are available for the synthesis of seven- [27–28], eight- [29–38], and nine- [35] membered aminocyclitols. Therefore, we were inspired to work on the development of the first synthesis of some C8-amino- and chloro-substituted cyclitols. Recently, we developed the first synthesis of various C8-amino- [29,31] and diaminocyclitol derivatives [30]. As part of our work involving the synthesis of C8-cyclitols, we report the stereospecific syntheses of two new 3-aminocyclooctanetriols and some chlorinated C8-cyclitols starting from *cis,cis*-1,3-cyclooctadiene.

## Results and Discussion

**Scheme 1:**
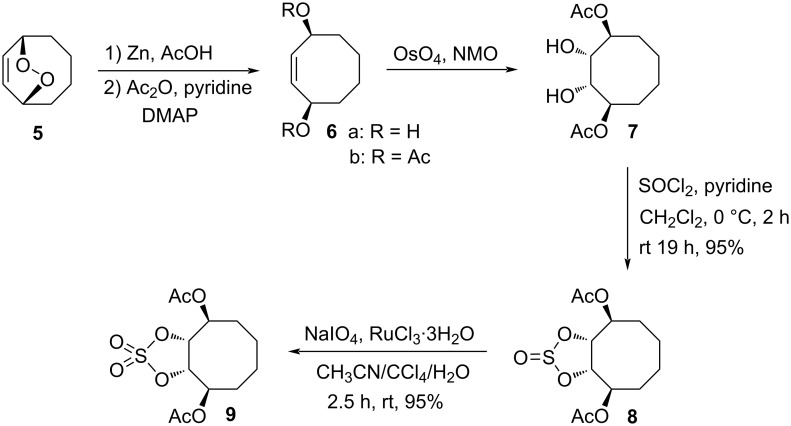
Synthesis of cyclic sulfate **9**.

For the synthesis of amino- and chlorocyclitols and their derivatives, we first selected endoperoxide **5** as the starting molecule, which was prepared using a procedure described in the literature [33] (Scheme 1). Among the most relevant precursors for the synthesis of aminocyclitols are cyclic sulfates [36–37,39–40] and they have been also used in the synthesis of C8-aminocarbasugars [36–37] recently. We envisioned that aminotriol **12** could be prepared by the reaction with sodium azide of the corresponding cyclic sulfate intermediate **9**, which contains the only stereocentre. The cyclic sulfate **9** could be synthesized from diacetatediol **7** [33].

For this purpose, the reduction of the endoperoxide **5** with zinc followed by acetylation of the hydroxy group and OsO_4_/NMO oxidation of the double bond gave diacetatediol **7** [33]. Treatment of diacetatediol **7** with thionyl chloride in pyridine gave the corresponding cyclic sulfite **8** in 95% yield (Scheme 1). Oxidation of the cyclic sulfite **8** with sodium periodate in the presence of ruthenium trichloride provided the corresponding cyclic sulfate **9** in 95% yield.

**Scheme 2:**
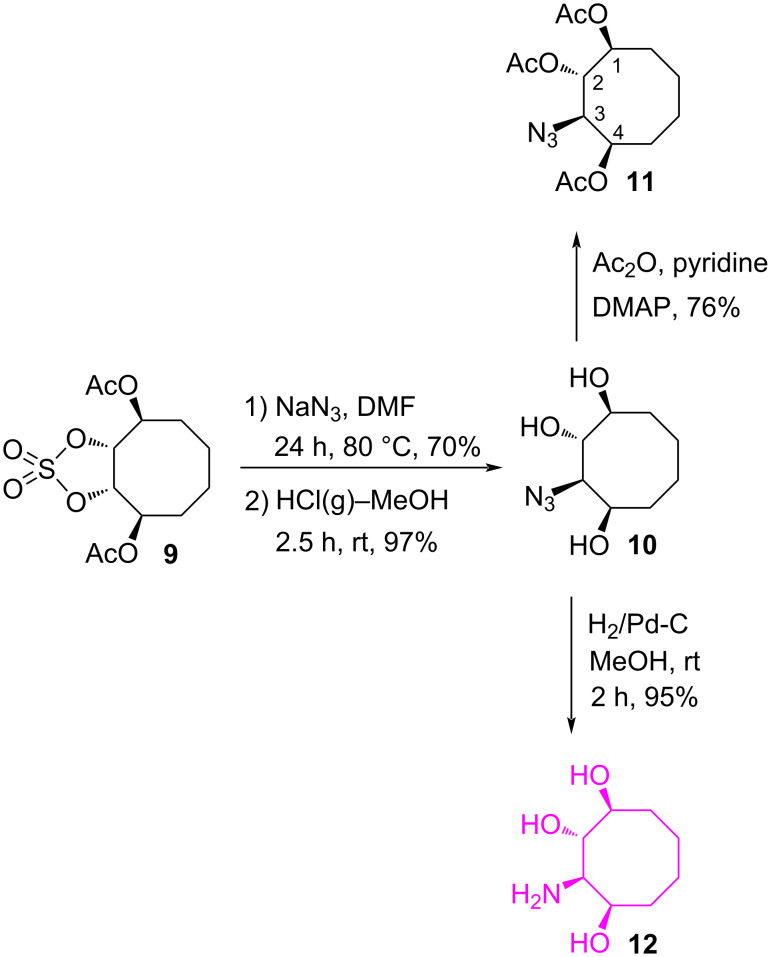
Synthesis of aminocyclooctanetriol **12**.

The cyclic sulfate moiety in **9** was reacted with sodium azide in DMF at 80 °C followed by acidic hydrolysis of the resulting acyclic sulfate ester to give azidotriol **10** as a single stereoisomer in 97% yield (Scheme 2). For further structural proof, the azidotriol **10** was converted into the corresponding triacetate **11** with acetic anhydride in pyridine and 4-(dimethylamino)pyridine (DMAP) (yield 76%). To determine the exact configurations of the substituents in **11**, we made full assignments for the H-3 and the acetoxy protons with the help of the 1D and 2D NMR experiments. First, protons H-3 and H-1 in the triacetate **11** were irradiated separately at their resonance frequencies and the changes in the spectrum were observed. Upon irradiation at the resonance frequency of the proton H-3 at 3.87 ppm there is no change in the multiplet at 5.04–4.97 ppm. However, in the multiplet part at 5.24–5.15 ppm, some splittings disappeared. This experiment clearly shows that the proton H-3 has couplings to both acetoxy protons H-2 and H-4. Furthermore, the proton H-3 resonates as a doublet of doublets with coupling constants of *J* = 8.8 and 2.7 Hz, clearly indicating that H-3 and H-2 with a large coupling constant (*J*_2,3_ = 8.8 Hz) are *trans* to each other. The small coupling constant (*J*_3,4_ = 2.7 Hz) between H-3 and H-4 shows the *cis* relationship between those protons. The configuration of the azide group in **11** was also confirmed by the cross peak between the proton H-3 and the protons H-2 and H-4 in the COSY spectrum. Moreover, the fact that the proton H-3 gives positive NOE clearly indicates that the proton H-3 should have a *cis* configuration relative to the proton H-1. On the other hand, the fact that the proton H-1 gives a positive NOE’s clearly indicates that the proton H-1 should have a *cis* configuration relative to the protons H-3 and H-4. Next, the reduction of azidotriol **10** by hydrogenation afforded the target aminotriol **12** in 95% yield.

**Scheme 3:**
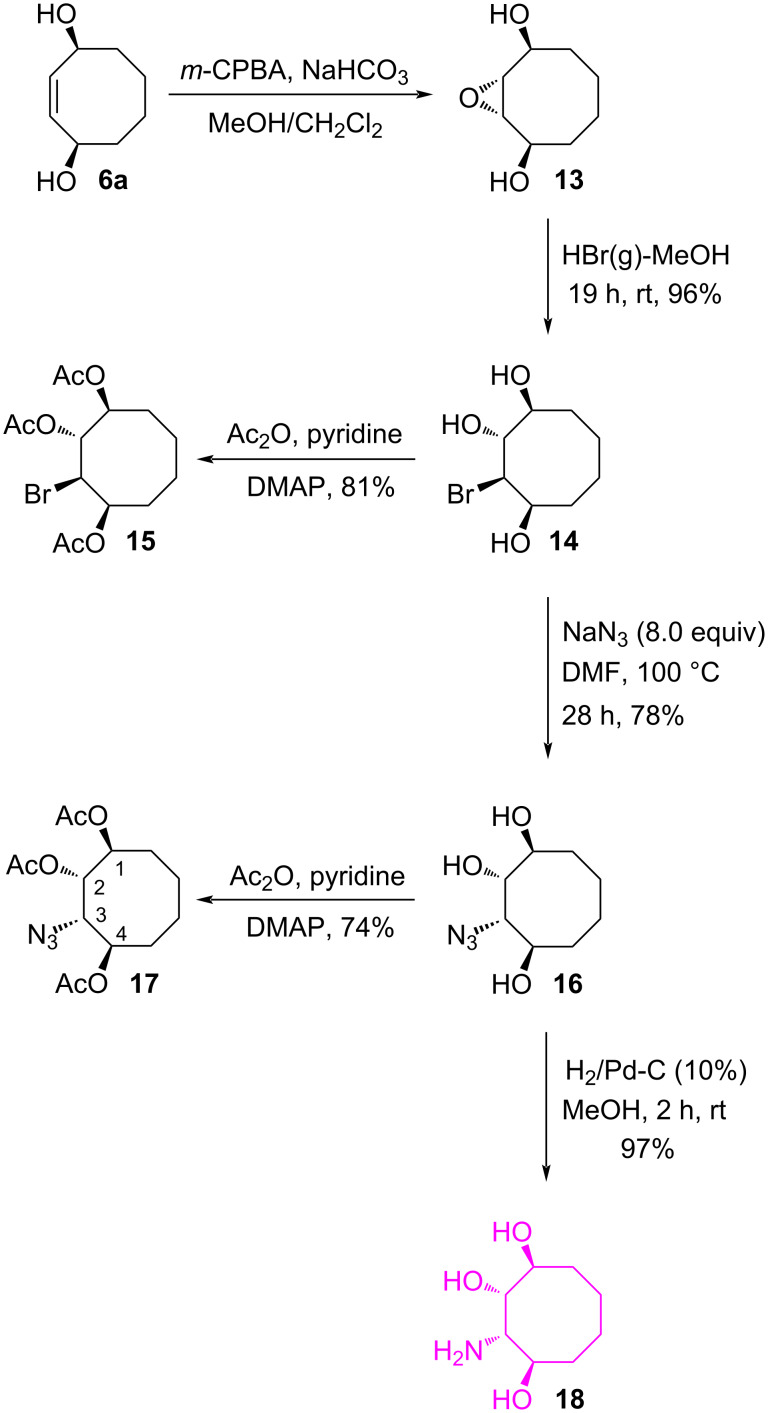
Synthesis of aminocyclooctanetriol **18**.

For the synthesis of the other aminocyclooctanetriol **18**, the diol **6a** [33] was reacted with *m*-CPBA to give *trans*-epoxide isomer **13** [33] (79% yield) as the sole product (Scheme 3). Ring opening of *trans*-epoxide **13** by HBr(g)–MeOH gave bromotriol **14**, which is an ideal substrate for the synthesis of the aminocyclooctanetriol **18**. For structural proof, bromotriol **14** was converted into the corresponding acetate **15** using Ac_2_O in pyridine and DMAP (81%).

Next, to introduce the azido group in a *cis*-configuration, the bromotriol **14** was treated with sodium azide in DMF at 100 °C to afford azidotriol **16** as a single product in 78% yield. Compound **16** was transformed into the corresponding triacetate **17** for full characterization of the structure (Scheme 3). The position of the azide group in **17** was confirmed by the help of the COSY spectrum. The diagonal peak at 3.95 ppm has cross peaks with the protons resonating at 4.96 and 5.47 ppm, respectively. Analysis of these cross peaks shows that the cross peak at 5.47 ppm is weaker. This weak correlation is due to the small coupling constant (*J* = 2.2 Hz). On the other hand, the resonance signal of H-3 appears as a doublet of doublets at 3.95 ppm with coupling constants of *J* = 8.6 and 2.2 Hz. The large coupling constant (*J* = 8.6 Hz) clearly supports the *trans* relation of the protons H-3 and H-4 and the small coupling constant (*J* = 2.2 Hz) the *cis* relation of the protons H-3 and H-2. Finally, the desired aminocyclooctanetriol **18** was obtained by hydrogenation of the azide functionality in compound **16** in 97% yield.

**Scheme 4:**
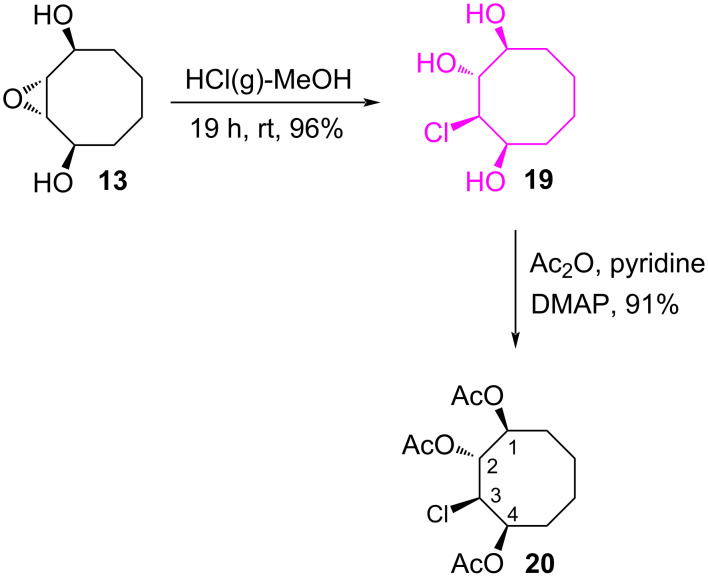
Synthesis of chlorocyclooctanetriol **20**.

In the second part of this work, we turned our attention to the stereospecific synthesis of chlorocyclooctanetriol **19** starting from the *trans*-epoxide **13** (Scheme 4). The hydroxy groups in **19** were acetylated to give **20** for further characterization of the structure. The position of the chlorine atom in **20** was confirmed with the help of the COSY spectra. The resonance signal of H-3 appears as a doublet of doublets at 4.35 ppm with coupling constants of *J* = 9.0 and 2.5 Hz. The large coupling constant (*J* = 9.0 Hz) clearly supports the *trans* relation of the protons H-3 and H-2 and the small coupling constant (*J* = 2.5 Hz) the *cis* relation of the protons H-3 and H-4.

**Scheme 5:**
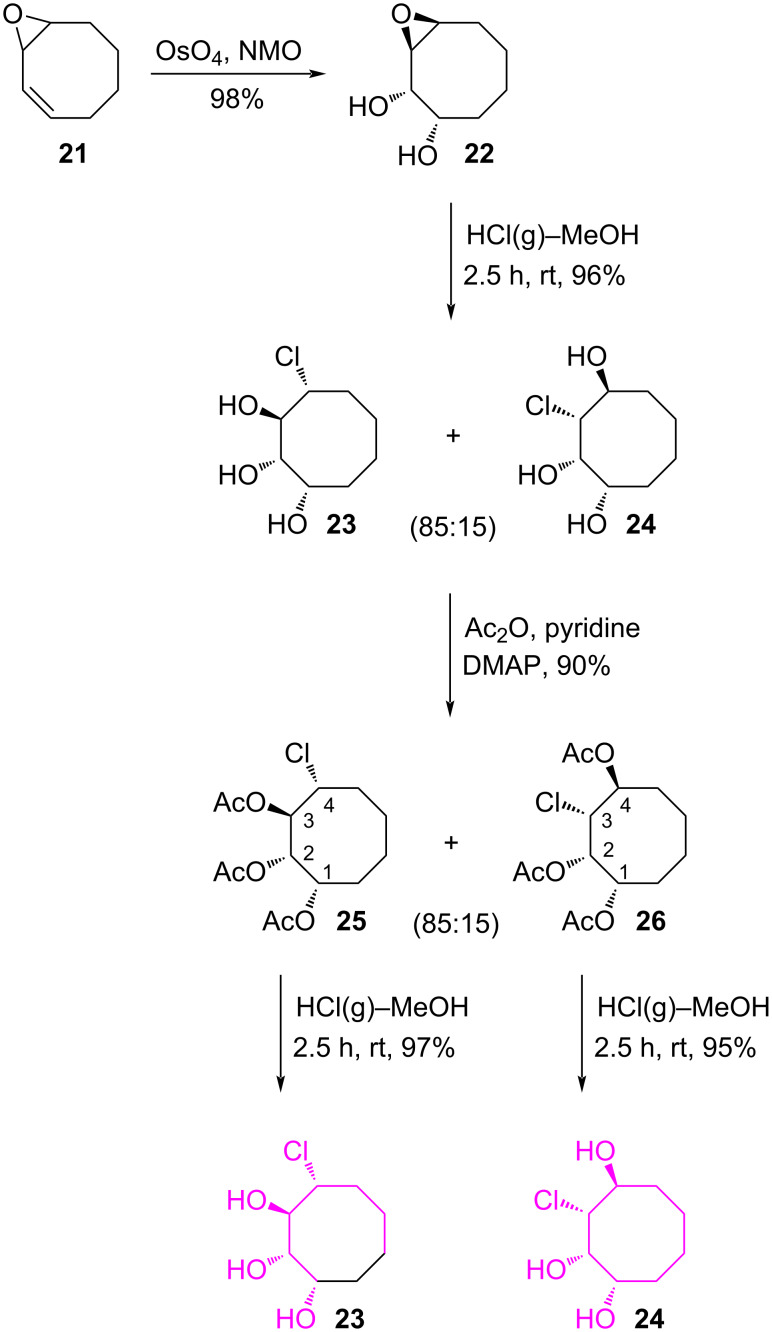
Synthesis of chlorocyclooctanetriols **23** and **24**.

As an alternate method for the synthesis of a novel chlorocyclooctanetriol isomer, epoxy-diol **22**, which was synthesized in our previous work [31], was hydrolysed by HCl(g) in MeOH, resulting in the formation of two chlorocyclooctanetriol isomers **23** and **24** in an 85:15 ratio (^1^H NMR) in 96% combined yield (Scheme 5). Chlorotriols **23** and **24** were transformed into the corresponding triacetates **25** and **26** for full characterization of their structures. A mixture of isomeric triacetates **25** and **26** was isolated by column chromatography in 74% and 12% yields, respectively. The structures and configurations of these compounds were assigned using ^1^H NMR and 2D NMR spectroscopic data. The position of the chlorine atom in **25** was confirmed with the help of the COSY spectra. The diagonal peak at 4.26 ppm has cross peaks with the protons resonating at 2.15 and 5.62 ppm, respectively. Analysis of these cross peaks shows that the cross peak at 5.62 ppm is strong. This strong correlation is due to the large coupling constant (*J* = 8.7 Hz). The fact that the proton H-4 appears as a doublet of doublet of doublets with coupling constants of *J* = 13.1, *J* = 8.7, and 3.3 Hz also supports the *trans* relation (*J* = 8.7 Hz) of the protons H-4 and H-3. Similarly, the configuration of the chlorine atom and the acetoxy groups in **26** was determined with ^1^H NMR and COSY spectra. Finally, deacetylation of chlorotriacetates **25** and **26** was carried out with HCl(g) in MeOH to give the free chlorotriols derivatives **23** and **24**.

## Conclusion

The synthesis of two stereoisomeric 3-aminocyclooctanetriols **12** and **18** and their halocyclitol derivatives **14**, **19**, **23**, and **24** was achieved for the first time concisely and efficiently from *cis,cis*-1,3-cyclooctadiene. The nitrogen functionalities were introduced by the substitution with NaN_3_ of the corresponding cyclic sulfate and bromo groups, while the halogen functionality was introduced to the molecule by opening of the epoxide ring with HBr(g) or HCl(g) in MeOH.

## Supporting Information

File 1 Experimental section, ^1^H and ^13^C NMR spectra for all new compounds, as well as selected 2D NMR spectra.
