# Supplementary material for: Stereoselective syntheses of 3-aminocyclooctanetriols and halocyclooctanetriols
Source: Beilstein J Org Chem. 2021 Mar 11;17:705–10. doi: 10.3762/bjoc.17.59 (PMC7961859; doi:10.3762/bjoc.17.59)

BEILSTEIN JOURNAL OF ORGANIC CHEMISTRY

Supporting Information

for

Stereoselective syntheses of 3-aminocyclooctanetriols and halocyclooctanetriols

Emine Salamci and Yunus Zozik

Beilstein J. Org. Chem. **2021,** *17,* 705–710. doi:10.3762/bjoc.17.59

Experimental section, ^{1}H and ^{13}C NMR spectra for all new compounds, as well as selected 2D NMR spectra

License and Terms: This is a supporting information file under the terms of the Creative Commons Attribution License (https://creativecommons.org/licenses/by/4.0). Please note that the reuse, redistribution and reproduction in particular requires that the author(s) and source are credited and that individual graphics may be subject to special legal provisions.
The license is subject to the *Beilstein Journal of Organic Chemistry* terms and conditions: (https://www.beilstein-journals.org/bjoc/terms)

Table of Contents

A. Experimental section S3

General information S3

(2*s*,3a*R*(*S*),4*S*(*R*),9*R*(*S*),9a*S*(*R*))-2-oxidooctahydrocycloocta[*d*][1,3,2]dioxathiole-4,9-diyl diacetate (**8**) S3

(3a*R*(*S*),4*S*(*R*),9*R*(*S*),9a*S*(*R*))-2,2-dioxidooctahydrocycloocta[*d*][1,3,2]dioxathiole-4,9-diyl diacetate (**9**) S3

(1*S*(*R*),2*S*(*R*),3*R*(*S*),4*R*(*S*))-3-azidocyclooctane-1,2,4-triol (**10**) S4

(1*S*(*R*),2*S*(*R*),3*R*(*S*),4*R*(*S*))-3-azidocyclooctane-1,2,4-triyl triacetate (**11**). General Procedure for the Acetylation of Hydroxyl Groups S4

(1*S*(*R*),2*S*(*R*),3*R*(*S*),4*R*(*S*))-3-aminocyclooctane-1,2,4-triol (**12**). General Procedure for Hydrogenation S5

(1*R*(*S*),2*S*(*R*),7*R*(*S*),8*S*(*R*))-9-oxabicyclo[6.1.0]nonane-2,7-diol (**13**) S5

(1*S*(*R*),2*R*(*S*),3*R*(*S*),4*R*(*S*))-3-bromocyclooctane-1,2,4-triol (**14**) S5

(1*S*(*R*),2*R*(*S*),3*R*(*S*),4*R*(*S*))-3-bromocyclooctane-1,2,4-triyl triacetate (**15**) S6

(1*S*(*R*),2*S*(*R*),3*S*(*R*),4*R*(*S*))-3-azidocyclooctane-1,2,4-triol (**16**) S6

(1*S*(*R*),2*S*(*R*),3*S*(*R*),4*R*(*S*))-3-azidocyclooctane-1,2,4-triyl triacetate (**17**) S6

(1*S*(*R*),2*S*(*R*),3*S*(*R*),4*R*(*S*))-3-aminocyclooctane-1,2,4-triol (**18**) S7

(1*S*(*R*),2*R*(*S*),3*R*(*S*),4*R*(*S*))-3-chlorocyclooctane-1,2,4-triol (**19**) S7

(1*S*(*R*),2*R*(*S*),3*R*(*S*),4*R*(*S*))-3-chlorocyclooctane-1,2,4-triyl triacetate (**20**) S7

Acetylation and reaction of epoxy-diol **22** with HCl(g)-MeOH S8

(1*S*(*R*),2*S*(*R*),3*R*(*S*),4*R*(*S*))-4-chlorocyclooctane-1,2,3-triyl triacetate (**25**) S8

(1*S*(*R*),2*S*(*R*),3*R*(*S*),4*S*(*R*))-3-chlorocyclooctane-1,2,4-triyl triacetate (**26**) S8

(1*R*(*S*),2*R*(*S*),3*S*(*R*),4*S*(*R*))-4-chlorocyclooctane-1,2,3-triol (**23**) S9

(1*R*(*S*),2*R*(*S*),3*S*(*R*),4*R*(*S*))-3-chlorocyclooctane-1,2,4-triol (**24**) S9

References S9

B. NMR Spectra S10

(2*s*,3a*R*(*S*),4*S*(*R*),9*R*(*S*),9a*S*(*R*))-2-oxidooctahydrocycloocta[*d*][1,3,2]dioxathiole-4,9-diyl diacetate (**8**): $CDCl_3$ (^{1}H NMR and ^{13}C NMR) S10

(2s,3a*R*(*S*),4*S*(*R*),9*R*(*S*),9a*S*(*R*))-2-oxidooctahydrocycloocta[*d*][1,3,2]dioxathiole-4,9-diyl diacetate (**8**): $CDCl_3$-HMQC S11

(2s,3a*R*(*S*),4*S*(*R*),9*R*(*S*),9a*S*(*R*))-2-oxidooctahydrocycloocta[*d*][1,3,2]dioxathiole-4,9-diyl diacetate (**8**):$CDCl_3$-COSY S12

(3a*R*(*S*),4*S*(*R*),9*R*(*S*),9a*S*(*R*))-2,2-dioxidooctahydrocycloocta[*d*][1,3,2]dioxathiole-4,9-diyl diacetate (**9**): $CDCl_3$ (^{1}H NMR and ^{13}C NMR) S13

1*S*(*R*),2*S*(*R*),3*R*(*S*),4*R*(*S*))-3-azidocyclooctane-1,2,4-triol (**10**): D_2O (^{1}H NMR and ^{13}C NMR) S14

(1*S*(*R*),2*S*(*R*),3*R*(*S*),4*R*(*S*))-3-azidocyclooctane-1,2,4-triyl triacetate (**11**): $CDCl_3$ (^{1}H NMR and Double Resonance) S15

(1*S*(*R*),2*S*(*R*),3*R*(*S*),4*R*(*S*))-3-azidocyclooctane-1,2,4-triyl triacetate (**11**): $CDCl_3$ (^{13}C NMR) S16

(1*S*(*R*),2*S*(*R*),3*R*(*S*),4*R*(*S*))-3-azidocyclooctane-1,2,4-triyl triacetate (**11**): NOE-Dif spectra S17

(1*S*(*R*),2*S*(*R*),3*R*(*S*),4*R*(*S*))-3-azidocyclooctane-1,2,4-triyl triacetate (**11**): HMQC S19

(1*S*(*R*),2*S*(*R*),3*R*(*S*),4*R*(*S*))-3-azidocyclooctane-1,2,4-triyl triacetate (**11**): COSY S20

(1*S*(*R*),2*S*(*R*),3*R*(*S*),4*R*(*S*))-3-aminocyclooctane-1,2,4-triol (**12**): CD_3OD (^{1}H NMR and ^{13}C NMR) S21

(1*S*(*R*),2*R*(*S*),3*R*(*S*),4*R*(*S*))-3-bromocyclooctane-1,2,4-triol (**14**): CD_3OD (^{1}H NMR and ^{13}C NMR) S22

(1*S*(*R*),2*R*(*S*),3*R*(*S*),4*R*(*S*))-3-bromocyclooctane-1,2,4-triyl triacetate (**15**): $CDCl_3$ (^{1}H NMR and Double Resonance) S23

(1*S*(*R*),2*R*(*S*),3*R*(*S*),4*R*(*S*))-3-bromocyclooctane-1,2,4-triyl triacetate (**15**): $CDCl_3$ (^{13}C NMR) S24

(1*S*(*R*),2*S*(*R*),3*S*(*R*),4*R*(*S*))-3-azidocyclooctane-1,2,4-triol (**16**): D_2O (^{1}H NMR and ^{13}C NMR) S25

(1*S*(*R*),2*S*(*R*),3*S*(*R*),4*R*(*S*))-3-azidocyclooctane-1,2,4-triyl triacetate (**17**): $CDCl_3$ (^{1}H NMR and Double Resonance) S26

(1*S*(*R*),2*S*(*R*),3*S*(*R*),4*R*(*S*))-3-azidocyclooctane-1,2,4-triyl triacetate (**17**): $CDCl_3$ (^{13}C NMR) S27

(1*S*(*R*),2*S*(*R*),3*S*(*R*),4*R*(*S*))-3-azidocyclooctane-1,2,4-triyl triacetate (**17**): $CDCl_3$-HMQC S28

(1*S*(*R*),2*S*(*R*),3*S*(*R*),4*R*(*S*))-3-azidocyclooctane-1,2,4-triyl triacetate (**17**): $CDCl_3$-COSY S29

(1*S*(*R*),2*S*(*R*),3*S*(*R*),4*R*(*S*))-3-aminocyclooctane-1,2,4-triol (**18**): D_2O (^{1}H NMR and ^{13}C NMR) S30

(1*S*(*R*),2*R*(*S*),3*R*(*S*),4*R*(*S*))-3-chlorocyclooctane-1,2,4-triol (**19**): D_2O (^{1}H NMR and ^{13}C NMR) S31

(1*S*(*R*),2*R*(*S*),3*R*(*S*),4*R*(*S*))-3-chlorocyclooctane-1,2,4-triyl triacetate (**20**): $CDCl_3$ (^{1}H NMR and Double Resonance) S32

(1*S*(*R*),2*R*(*S*),3*R*(*S*),4*R*(*S*))-3-chlorocyclooctane-1,2,4-triyl triacetate (**20**): $CDCl_3$ (^{13}C NMR) S33

(1*S*(*R*),2*R*(*S*),3*R*(*S*),4*R*(*S*))-3-chlorocyclooctane-1,2,4-triyl triacetate (**20**): $CDCl_3$-HMQC S34

1*S*(*R*),2*R*(*S*),3*R*(*S*),4*R*(*S*))-3-chlorocyclooctane-1,2,4-triyl triacetate (**20**): $CDCl_3$-COSY S35

(1*R*(*S*),2*R*(*S*),3*S*(*R*),4*S*(*R*))-4-chlorocyclooctane-1,2,3-triol (**23**): D_2O (^{1}H NMR and ^{13}C NMR) S36

(1*R*(*S*),2*R*(*S*),3*S*(*R*),4*R*(*S*))-3-chlorocyclooctane-1,2,4-triol (**24**): D_2O (^{1}H NMR and ^{13}C NMR) S37

(1*R*(*S*),2*R*(*S*),3*S*(*R*),4*S*(*R*))-4-chlorocyclooctane-1,2,3-triyl triacetate (**25**): $CDCl_3$ (^{1}H NMR and ^{13}C NMR) S38

1*R*(*S*),2*R*(*S*),3*S*(*R*),4*S*(*R*))-4-chlorocyclooctane-1,2,3-triyl triacetate (**25**): $CDCl_3$-HMQC S39

(1*R*(*S*),2*R*(*S*),3*S*(*R*),4*S*(*R*))-4-chlorocyclooctane-1,2,3-triyl triacetate (**25**): $CDCl_3$-COSY S40

(1*R*(*S*),2*R*(*S*),3*S*(*R*),4*R*(*S*))-3-chlorocyclooctane-1,2,4-triyl triacetate (**26**): $CDCl_3$ (^{1}H NMR and ^{13}C NMR) S41

(1*R*(*S*),2*R*(*S*),3*S*(*R*),4*R*(*S*))-3-chlorocyclooctane-1,2,4-triyl triacetate (**26**): $CDCl_3$-HMQC S42

(1*R*(*S*),2*R*(*S*),3*S*(*R*),4*R*(*S*))-3-chlorocyclooctane-1,2,4-triyl triacetate (**26**): $CDCl_3$-COSY S43

A. Experimental section

General information

Melting points are uncorrected. Infrared spectra were obtained from solution in 0.1 mm cells or KBr pellets on an FTIR Mattson 1000 instrument. The ^{1}H and ^{13}C NMR spectra were recorded on 400 (100) MHz Varian or 400 (100) MHz Bruker spectrometer and are reported in δ units with $SiMe_4$ as internal standard. Elemental analyses were carried out on a LECO's CHNS-932 instrument. Melting points were determined on a GallenKamp MPD 350. Column chromatography was performed on silica gel (60 mesh, Merck). TLC was carried out on Merck 0.2 mm silica gel 60 F_{254} analytical aluminium plates.

7,8-Dioxabicyclo[4.2.2]dec-9-ene (5), ***cis*-2-cyclooctene-1,4-diol (6a)**, ***cis*-1,4-diacetoxy-2-cyclooctene (6b)** and **(1*R*(*S*),2*R*(*S*),3*S*(*R*),4*S*(*R*))-2,3-dihydroxycyclooctane-1,4-diyl diacetate (7)** were prepared as described in the literature [1].

(2*s*,3a*R*(*S*),4*S*(*R*),9*R*(*S*),9a*S*(*R*))-2-Oxidooctahydrocycloocta[*d*][1,3,2]dioxathiole-4,9-diyl diacetate (8): To a cooled (at 0 °C), magnetically stirred solution of diacetate diol **7** (1.60 g, 6.15 mmol) and pyridine (1.46 g, 18.51 mmol) in CH_2Cl_2 (20 mL) was added a dichloromethane solution (5 mL) of thionyl chloride (2.20 g, 18.51 mmol) dropwise over a period of 10 min. Stirring was continued for 2 h at 0 °C and stirred at room temperature for 19 h (the reaction was followed by TLC). To the reaction mixture was added water (25 mL) and extracted with ethyl acetate (4 × 30 mL). The combined organic extracts were washed with 1 N HCl solution (50 mL) and saturated $NaHCO_3$ solution (20 mL) and NaCl solution (20 mL). The organic solution was dried over Na_2SO_4. Evaporation of the solvents gave pure **8** (1.79 g, 95%). Sulfite **8** was recrystallized from CH_2Cl_2/*n*-hexane (9:1) as a colourless crystal; mp 146-147 °C. ^{1}H-NMR (400 MHz, $CDCl_3$): δ 5.30-5.20 (m, 2H, CH-OAc), 5.00-4.93 (m, 2H, CH-SO_3), 2.07 (s, 6H, 2xOAc), 2.06-1.48 (series of m, 8H, CH_2). ^{13}C-NMR (100 MHz, $CDCl_3$): δ 170.1, 82.2, 69.6, 32.3, 22.6, 21.2; IR (KBr, cm^{-1}): 2951, 2877, 1744, 1465, 1451, 1429, 1374, 1323, 1235, 1205, 1029, 996, 969, 955. Anal. Calcd for $C_{12}H_{18}O_7S$ (306.08): C 47.05; H 5.92; S 10.47; found C 46.89; H 5.81; S 10.47.

(3a*R*(*S*),4*S*(*R*),9*R*(*S*),9a*S*(*R*))-2,2-Dioxidooctahydrocycloocta[*d*][1,3,2]dioxathiole-4,9-diyl diacetate (9): The sulfite **8** (1.81 g, 5.91 mmol) was dissolved in CCl_4 (20 mL). To mixture

was added $NaIO_4$ (1.90 g, 8.88 mmol) and acetonitrile (20 mL) and water (20 mL) and $RuCl_3 \cdot 3H_2O$ (15 mg). The reaction mixture was stirred 2.5 h at room temperature. The mixture was extracted with ethyl acetate (4 × 30 mL). The organic solution was dried over Na_2SO_4. Evaporation of the solvents gave pure **9** (1.80 g, 95%). Sulfate **9** was recrystallized from CH_2Cl_2/*n*-hexane (9:1) as a colourless crystal; mp 130-131 °C. ^{1}H-NMR (400 MHz, $CDCl_3$): δ 5.52-5.41 (m, 2H, CH-OAc), 5.12-5.05 (m, 2H, CH-SO_4), 2.09 (s, 6H, 2xOAc), 2.09-1.46 (series of m, 8H, CH_2). ^{13}C-NMR (100 MHz, $CDCl_3$): δ 169.6, 84.5, 69.5, 31.4, 22.6, 21.1; IR (KBr, cm^{-1}): 2950, 2900, 1753, 1477, 1390, 1232, 1106, 1030, 957. Anal. Calcd for $C_{12}H_{18}O_8S$ (322.07): C 44.72; H 5.63; S 9.95; found C 44.91; H 5.48; S 10.04.

(1*S*(*R*),2*S*(*R*),3*R*(*S*),4*R*(*S*))-3-Azidocyclooctane-1,2,4-triol (10): A mixture of the sulfate **9** (1.00 g, 3.10 mmol) and NaN_3 (1.00 g, 15.38 mmol) in absolute DMF (10 mL) was stirred under nitrogen for 24 h at 80 °C. The mixture was cooled to room temperature and THF (20 mL) was added. Then concentrated H_2SO_4 (4 drops) and water (4 drops) were added to the stirred suspension. After 40 min $NaHCO_3$ (400 mg) was added and the reaction mixture was stirred for 40 min. Filtration through a Celite and silica gel bed and concentration of the filtrate under reduced pressure provided a viscous oil. Column chromatography (silica gel, 100 g) eluting with MeOH/CH_2Cl_2 (3:97) gave azidosulfate diacetate (0.8 g, 70%) as a white solid. Absolute methanol (10 mL) containing 20% HCl gas was added into the azidosulfate diacetate (220 mg, 0.60 mmol). The mixture was stirred at room temperature for 2.5 h. Evaporation of solvent gave 118 mg (97% yield) of azidotriol **10** as a colourless oil. ^{1}H-NMR (400 MHz, D_2O): δ 4.07 (dt, *J* = 8.7, 2.3 Hz, 1H, H-4), 3.68-3.59 (m, 2H, H-1 and H-2), 3.59-3.52 (m, 1H, H-3), 1.90-1.30 (series of m, 8H, CH_2). ^{13}C-NMR (100 MHz, D_2O): δ 73.4, 72.1, 70.0, 69.8, 31.8, 30.7, 23.6, 20.5; IR (KBr, cm^{-1}): 3407, 2934, 1420, 1365, 1264, 1230, 1068, 1038, 959.

Anal. Calcd for $C_8H_{15}N_3O_3$ (201.11): C 47.75; H 7.51; N 20.88; found C 47.76; H 7.35; N 20.71.

(1*S*(*R*),2*S*(*R*),3*R*(*S*),4*R*(*S*))-3-Azidocyclooctane-1,2,4-triyl triacetate (11). General procedure for the acetylation of hydroxyl groups: In a similar manner as described in the literature [2], the azidotriol **10** (280 mg, 1.39 mmol) was dissolved in anhydrous pyridine (3 mL) and the solution was cooled 0 °C. Ac_2O (0.7 mL, 8.35 mmol) and 4-(dimethylamino)pyridine (DMAP) (2.8 mg) were added and the solution was stirred for 2.5 day at room temperature. The mixture was cooled to 0 °C and 2 N HCl (70 mL) solution was

added. The mixture was extracted with ether (6 × 30 mL). The combined organic extracts were washed with saturated $NaHCO_3$ solution (70 mL) and water (3 × 10 mL) and then dried (Na_2SO_4). After evaporation of solvent, chromatography of the mixture on a silica gel column eluting with EtOAc/*n*-hexane (3:97) gave 350 mg (76%) of triacetate **11** as a colourless oil. ^{1}H-NMR (400 MHz, $CDCl_3$): δ 5.24-5.15 (m, 2H, H-4 and H-2), 5.04-4.97 (m, 1H, H-1), 3.87 (dd, *J* = 8.8, 2.7 Hz, 1H, H-3), 2.06 (s, 3H), 2.05 (s, 3H) 1.98 (s, 3H), 2.10-1.58 (series of m, 8H, CH_2). ^{13}C-NMR (100 MHz, $CDCl_3$): δ 170.1, 170.0, 169.8, 72.7, 72.1, 71.3, 65.6, 31.2, 28.6, 23.5, 21.8, 21.1, 21.0, 20.8; IR (KBr, cm^{-1}): 2941, 2870, 2106, 1744, 1433, 1371, 1238, 1028, 974, 906. Anal. Calcd for $C_{14}H_{21}N_3O_6$ (327.14): C 51.37; H 6.47; N 12.84; found C 51.52; H 6.52; N 12.99.

(1*S*(*R*),2*S*(*R*),3*R*(*S*),4*R*(*S*))-3-Aminocyclooctane-1,2,4-triol (12). General procedure for hydrogenation: In a similar manner as described in the literature [2], into a 50 mL flask was placed palladium on charcoal (30 mg, 10%) and azidotriol **10** (250 mg, 1.24 mmol) in absolute methanol (30 mL). The reaction mixture was flushed with hydrogen gas (the air in the solvent was removed under vacuum, and then the flask was filled with hydrogen gas; this process was repeated three times). The resulting mixture was stirred at room temperature for 2 h under the hydrogen atmosphere. The catalyst was removed by filtration. Evaporation of the solvent gave pure aminotriol **12** (207 mg, 95%) as a colourless viscous oil. ^{1}H-NMR (400 MHz, CD_3OD): δ 3.99-3.91 (m, 1H, H-4), 3.75 (t, *J* = 8.4, 1H, H-2), 3.54-3.47 (m, H-1), 3.08 (dd, *J* = 7.9, 2.8 Hz, H-3), 1.96-1.34 (series of m, 8H, CH_2). ^{13}C-NMR (100 MHz, CD_3OD): δ 74.8, 69.8, 69.5, 55.1, 30.2, 30.1, 21.7, 20.8; IR (KBr, cm^{-1}): 3390, 2935, 1466, 1386, 1123, 1037. Anal. Calcd for $C_8H_{17}NO_3$ (175.12): C 54.84; H 9.78; N 7.99; found C 54.74; H 9.71; N 7.88.

(1*R*(*S*),2*S*(*R*),7*R*(*S*),8*S*(*R*))-9-Oxabicyclo[6.1.0]nonane-2,7-diol (13): Compound **13** was prepared as described in the literature [1].

(1*S*(*R*),2*R*(*S*),3*R*(*S*),4*R*(*S*))-3-Bromocyclooctane-1,2,4-triol (14): Absolute methanol (25 mL) containing 35% HBr gas was added into *trans*-epoxide **13** (1.30 g, 8.22 mmol) and stirred at 0 °C for 30 min. Then, the reaction mixture was stirred for 19 h at room temperature. After removal of the solvent, crude product was dissolved in 25 mL of methanol and added into excess $BaCO_3$ (10 g) for neutralization (pH = 8), and stirred magnetically at room temperature for 4 h. The solvent was removed under reduced pressure and the residue

was dissolved in acetone and stirred for 5 min. The solid was filtered off. Evaporation of the solvent gave bromotriol **14** (1.89 g, 96%) as a colourless oil. ^{1}H-NMR (400 MHz, CD_3OD): δ 4.25 (dd, *J* = 8.7, 2.2 Hz, 1H, H-3), 4.05-3.97 (m, H-4), 3.93 (t, *J* = 8.6 Hz, 1H, H-2), 3.76-3.64 (m, H-1), 2.00-1.40 (series of m, 8H, CH_2). ^{13}C-NMR (100 MHz, CD_3OD): δ 76.6, 71.5, 69.1, 68.1, 35.1, 33.2, 23.3, 21.8; IR (KBr, cm^{-1}): 3418, 2986, 2930, 2866, 1456, 1371, 1216, 1171, 1055, 1019, 949. Anal. Calcd for $C_8H_{15}BrO_3$ (238.02): C 40.19; H 6.32; found C 40.09; H 6.40.

(1*S*(*R*),2*R*(*S*),3*R*(*S*),4*R*(*S*))-3-Bromocyclooctane-1,2,4-triyl triacetate (15): The bromotriol **14** (3.0 g, 12.55 mmol) was submitted to acetylation with Ac_2O and DMAP in pyridine following the method described above for the acetylation of **10** to give **15**: 3.75 g, 81%. Bromotriacetate **15** was recrystallized from CH_2Cl_2/hexane as colourless crystals; mp 92-94 °C. ^{1}H-NMR (400 MHz, $CDCl_3$): δ 5.46 (t, *J* = 9.0 Hz, 1H, H-2), 5.21-5.15 (m, 1H, H-4), 5.07 (ddd, *J* = 8.5, 7.3, 3.9 Hz, 1H, H-1), 4.42 (dd, *J* = 9.3, 2.4 Hz, 1H, H-3), 2.08 (s, 3H), 2.07 (s, 3H), 2.00 (s, 3H), 1.99-1.58 (series of m, 8H, CH_2). ^{13}C-NMR (100 MHz, $CDCl_3$): δ 170.2, 169.8, 169.7, 74.3, 71.8, 71.0, 57.3, 31.8, 30.5, 23.3, 21.9, 21.3, 21.0, 20.8; IR (KBr, cm^{-1}): 2938, 2882, 1743, 1469, 1447, 1380, 1233, 1090, 1033, 975. Anal. Calcd for $C_{14}H_{21}BrO_6$ (364.05): C 46.04; H 5.80; found C 46.06; H 5.66.

(1*S*(*R*),2*S*(*R*),3*S*(*R*),4*R*(*S*))-3-Azidocyclooctane-1,2,4-triol (16): To a magnetically stirred solution of **15** (1.26 g, 5.27 mmol) in DMF (15 mL) to the mixture was added NaN_3 (2.74 g, 42.16 mmol) and the mixture heated to 100 °C and was stirred at 100 °C for 28 h. The reaction mixture was filtered with methanol through a pad of silica gel in a sintered glass funnel. After evaporation of the solvents, chromatography of the mixture on a silica gel (100 g) column eluting with MeOH/CH_2Cl_2 (4:96) gave the azidotriol **16** (0.83 g, 78%) as a colourless oil. ^{1}H-NMR (400 MHz, D_2O): δ 3.90 (dd, *J* = 8.7, 2.5 Hz, H-2), 3.88-3.76 (m, 2H, H-1 and H-4), 3.69 (dd, *J* = 9.2, 2.5 Hz, 1H, H-3), 1.96-1.48 (series of m, 8H, CH_2). ^{13}C-NMR (100 MHz, D_2O): δ 74.9, 72.4, 71.7, 70.0, 32.5, 31.4, 23.8, 23.2; IR (KBr, cm^{-1}): 3399, 2932, 2872, 2114, 1453, 1416, 1359, 1260, 1161, 1092, 1053, 1001, 982. Anal. Calcd for $C_8H_{15}N_3O_3$ (201.11): C 47.75; H 7.51; N 20.88; found C 47.60; H, 7.41; N 20.79.

(1*S*(*R*),2*S*(*R*),3*S*(*R*),4*R*(*S*))-3-Azidocyclooctane-1,2,4-triyl triacetate (17): The azidotriol **16** (200 mg, 0.99 mmol) was submitted to acetylation with Ac_2O and DMAP in pyridine following the method described above for the acetylation of **10** to give **17**: 243 mg, 74%; as a

colourless oil. ^{1}H-NMR (400 MHz, $CDCl_3$): δ 5.47 (dd, $J_{2,1}$ = 9.4 Hz, $J_{2,3}$ = 2.2 Hz, 1H, H-2), 5.28-5.21 (m, 1H, H-1), 4.96 (t, J = 7.8 Hz, 1H, H-4), 3.95 (dd, J = 8.6 Hz, $J_{3,2}$ = 2.2 Hz, H-3), 2.10 (s, 6H, 2xOAc) 2.02 (s, 3H, OAc) 2.10-1.55 (series of m, 8H, CH_2). ^{13}C-NMR (100 MHz, $CDCl_3$): δ 170.0, 169.9, 169.7, 75.3, 72.6, 71.9, 65.4, 30.3, 28.4, 24.6, 21.7, 21.1, 21.0, 20.7; IR (KBr, cm^{-1}): 2943, 2887, 2118, 1742, 1472, 1445, 1371, 1253, 1201, 1094, 1033 960. Anal. Calcd for $C_{14}H_{21}N_3O_6$ (327.14): C 51.37; H, 6.47; N, 12.84; found C 51.43; H, 6.33; N, 12.73.

(1*S*(*R*),2*S*(*R*),3*S*(*R*),4*R*(*S*))-3-Aminocyclooctane-1,2,4-triol (18): The azidotriol **16** (200 mg, 0.99 mmol) was hydrogenated with Pd (10% on activated carbon; 25 mg) as described above for the synthesis of **12** to give aminotriol **18** (170 mg, 97%); as a colourless oil. ^{1}H-NMR (400 MHz, D_2O): δ 3.74 (ddd, J = 8.2, 6.6, 1.4 Hz, 1H, H-1), 3.61 (dd, J = 8.8, $J_{2,3}$ = 2.7 Hz, 1H, H-2), 3.60-3.54 (m, 1H, H-4) 2.90 (dd, $J_{3,4}$ = 9.4, $J_{3,2}$ = 2.7 Hz, 1H, H-3), 1.90-1.32 (series of m, 8H, CH_2). ^{13}C-NMR (100 MHz, D_2O): δ 76.5, 73.4, 72.8, 56.7, 32.9, 32.2, 24.7, 23.3; IR (KBr, cm^{-1}): 3390, 2935, 1466, 1386, 1123, 1037. Anal. Calcd for $C_8H_{17}NO_3$ (175.12): C 54.84; H 9.78; N 7.99; found C 54.74; H 9.66; N 7.89.

(1*S*(*R*),2*R*(*S*),3*R*(*S*),4*R*(*S*))-3-Chlorocyclooctane-1,2,4-triol (19): To a magnetically stirred solution of the *trans*-epoxide **13** (2.0 g, 12.64 mmol) in absolute methanol (5 mL) was cooled to 0 °C. Then, absolute methanol (30 mL) containing 20% HCl gas was added into the reaction mixture and stirred at room temperature for 19 h. Evaporation of the solvent under reduced pressure and crystallization of the residue from MeOH/ether (8:2) gave chlorotriol **19** as colourless crystals (2.37 g, 96%), mp 117-119 °C. ^{1}H-NMR (400 MHz, D_2O): δ 4.14 (dt, J = 9.0, $J_{3,4}$ = 2.8 Hz, H-4), 4.07 (dd, J = 8.6, $J_{3,4}$ = 2.8 Hz, H-3), 3.74 (t, J = 8.8 Hz, H-2), 3.60 (ddd, J = 9.3, 5.7, 4.0 Hz, H-1), 1.90-1.34 (series of m, 8H, CH_2). ^{13}C-NMR (100 MHz, D_2O): δ 74.8, 71.8, 71.4, 69.4, 33.0, 31.2, 23.6, 20.9; IR (KBr, cm^{-1}): 3292, 2934, 2875, 1459, 1402, 1322, 1295, 1256, 1234, 1215, 1144, 1040, 975. Anal. Calcd for $C_8H_{15}ClO_3$ (194.07): C 49.36; H 7.77; found C 49.42; H 7.75.

(1*S*(*R*),2*R*(*S*),3*R*(*S*),4*R*(*S*))-3-Chlorocyclooctane-1,2,4-triyl triacetate (20): The chlorotriol **19** (2.5 g, 12.84 mmol) was submitted to acetylation with Ac_2O and DMAP in pyridine following the method described above for the acetylation of **10** to give **20**: 3.76 g, 91%. Chlorotriacetate **20** was recrystallized from CH_2Cl_2/hexane (9:1) as colourless crystals, mp 75-76 °C. ^{1}H-NMR (400 MHz, $CDCl_3$): δ 5.39 (t, J = 8.8 Hz, H-2), 5.35 (dt, J = 9.7, $J_{3,4}$ = 2.5

Hz, H-4), 5.12-5.06 (m, H-1), 4.35 (dd, J = 9.0, $J_{3,4}$ = 2.5 Hz, H-3), 2.11 (s, 3H), 2.10 (s, 3H), 2.03 (s, 3H), 2.20-1.57 (series of m, 8H, CH_2). ^{13}C-NMR (100 MHz, $CDCl_3$): δ 170.3, 169.8, 169.8, 74.3, 71.4, 71.3, 64.1, 31.7, 29.1, 23.3, 21.9, 21.2, 21.0, 20.8; IR (KBr, cm^{-1}): 3292, 2934, 2875, 1459, 1402, 1322, 1295, 1256, 1215, 1144, 1097, 1040, 975, 948. Anal. Calcd for $C_{14}H_{21}ClO_6$ (320.10): C 52.42; H 6.60; found C 52.47; H 6.49.

Acetylation and reaction of epoxy-diol 22 with HCl(g)-MeOH: To a magnetically stirred solution of epoxydiol **22** (500 mg, 3.16 mmol) in absolute methanol (5 mL) was cooled to 0 °C. Then, Absolute methanol (15 mL) containing 40% HCl gas was added into the reaction mixture and stirred at room temperature for 2.5 h. Evaporation of the solvent gave a mixture of chlorotriol **23** and chlorotriol **24** (590 mg, total yield 96%). From ^{1}H NMR spectroscopy, it was observed that the mixture of **23** and **24** was in an 85:15 ratio. But these isomers could not be obtained in pure form, although all chromatographic purification methods were employed. Then, 450 mg (2.31 mmol) of the resultant mixture was submitted to acetylation with Ac_2O and DMAP in pyridine following the method described above for the acetylation of **10** to give a mixture of the diacetate isomer (670 mg), total yield 90%. Chromatography of the mixture on a silica gel column (80 g) eluting with EtOAc/hexane (10:90) gave the first fraction of chlorotriacetate **26** (90 mg, 12%, as a colourless oil) and the second chlorotriacetate **25** (0.55 g, 74%, as a colourless oil).

(1*S*(*R*),2*S*(*R*),3*R*(*S*),4*R*(*S*))-4-Chlorocyclooctane-1,2,3-triyl triacetate (25): ^{1}H-NMR (400 MHz, $CDCl_3$): δ 5.62 (dd, J = 9.7, 8.1 Hz, H-3), 5.25 (dt, J = 7.3, $J_{1,2}$ = 2.3 Hz, H-1), 5.05 (dd, J = 8.0, $J_{1,2}$ = 2.3 Hz, H-2) 4.26 (ddd, J = 13.1, 8.7, 3.3 Hz, H-4), 2.09 (s, 3H), 2.07 (s, 3H), 2.03 (s, 3H), 2.25-1.50 (series of m, 8H, CH_2). ^{13}C-NMR (100 MHz, $CDCl_3$): δ 170.2, 169.7, 169.0, 71.9, 71.4, 70.6, 62.3, 30.0, 27.5, 21.5, 20.9, 20.5, 20.5, 19.8; IR (KBr, cm^{-1}): 2942, 2863, 1747, 1434, 1372, 1251, 1053, 966. Anal. Calcd for $C_{14}H_{21}ClO_6$ (320.10): C 52.42; H 6.60; found C 52.53; H 6.70.

(1*S*(*R*),2*S*(*R*),3*R*(*S*),4*S*(*R*))-3-Chlorocyclooctane-1,2,4-triyl triacetate (26): ^{1}H-NMR (400 MHz, $CDCl_3$): δ 5.71 (t, J = 2.3 Hz, H-2), 5.25-5.18 (m, H-4) 5.15-5.09 (m, H-1) 4.34 (dd, J = 9.5, 1.9 Hz, H-3), 2.18 (s, 3H), 2.10 (s, 3H), 2.05 (s, 3H), 2.00-1.34 (series of m, 8H, CH_2). ^{13}C-NMR (100 MHz, $CDCl_3$): δ 170.2, 169.9, 169.7, 75.5, 74.0, 73.6, 62.5, 29.7, 27.1, 22.7, 22.2, 21.0, 21.0, 20.7; IR (KBr, cm^{-1}): 2937, 1740, 1645, 1435, 1370, 1239, 1092, 1035, 963. Anal. Calcd for $C_{14}H_{21}ClO_6$ (320.10): C 52.42; H 6.60; found C 52.30; H 6.64.

(1*R*(*S*),2*R*(*S*),3*S*(*R*),4*S*(*R*))-4-Chlorocyclooctane-1,2,3-triol (23): To a magnetically stirred solution of chlorotriacetate **25** (200 mg, 0.62 mmol) in absolute methanol (3 mL) was cooled to 0 °C. Then, Absolute methanol (15 mL) containing 20% HCl gas was added into the reaction mixture and stirred at room temperature for 2.5 h. Evaporation of the solvent gave chlorotriol **23** (118 mg, yield 97%). The chlorotriol **23** was recrystallized from CH_2Cl_2/hexane (9:1) as colourless crystals, mp 107-108 °C. ^{1}H-NMR (400 MHz, $CDCl_3$): δ 4.03-3.96 (m, H-1), 3.89 (dt, J = 7.8, 2.6 Hz, H-4), 3.85 (dd, J = 9.6, $J_{3,2}$ = 7.9 Hz, H-3) 3.54 (dd, $J_{2,3}$ = 7.9, 2.7 Hz, H-2), 2.00-1.20 (series of m, 8H, CH_2). ^{13}C-NMR (100 MHz, $CDCl_3$): δ 72.5, 72.0, 71.2, 67.9, 30.5, 28.9, 21.5, 20.3; IR (KBr, cm^{-1}): 3400, 2925, 2878, 2863, 2731, 1467, 1458, 1392, 1372, 1284, 1267, 1221, 1067, 980, 959. Anal. Calcd for $C_8H_{15}ClO_3$ (194.07): C 49.36; H 7.77; found C 49.43; H 7.66.

(1*R*(*S*),2*R*(*S*),3*S*(*R*),4*R*(*S*))-3-Chlorocyclooctane-1,2,4-triol (24): The chlorotriacetate **26** (300 mg, 0.93 mmol) was submitted to hydrolysis with HCl(g)-MeOH following the method described above for the hydrolysis of **25** to give **24**: 173 mg, 95%, as a colourless oil. ^{1}H-NMR (400 MHz, $CDCl_3$): δ 4.17 (dd, J = 3.0, 1.6 Hz, H-2), 4.04 (dd, $J_{3,4}$ = 9.2, J = 1.4 Hz, H-3), 3.92-3.86 (m, H-4), 3.64 (dt, J = 11.9, 3.6 Hz, 1H), 2.00-0.95 (series of m, 8H, CH_2). ^{13}C-NMR (100 MHz, $CDCl_3$): δ 75.7, 74.5, 74.4, 69.7, 30.9, 28.4, 22.8, 21.9; IR (KBr, cm^{-1}): 3410, 2983, 2934, 1466, 1370, 1254, 1218, 1163, 1121, 1051, 961. Anal. Calcd for $C_8H_{15}ClO_3$ (194.07): C 49.36; H 7.77; found C 49.26; H 7.69.

References

1 Salamci, E. *Tetrahedron* **2010**, *66*, 4010-4015.

2 Karavaizoglu, U. N.; Salamci, E. *New J. Chem*. **2020**, *44*, 17976-17983.

B. NMR Spectra

(2s,3a*R*(*S*),4*S*(*R*),9*R*(*S*),9a*S*(*R*))-2-Oxidooctahydrocycloocta[*d*][1,3,2]dioxathiole-4,9-diyl diacetate (8): $CDCl_3$ (^1H NMR and ^{13}C NMR)

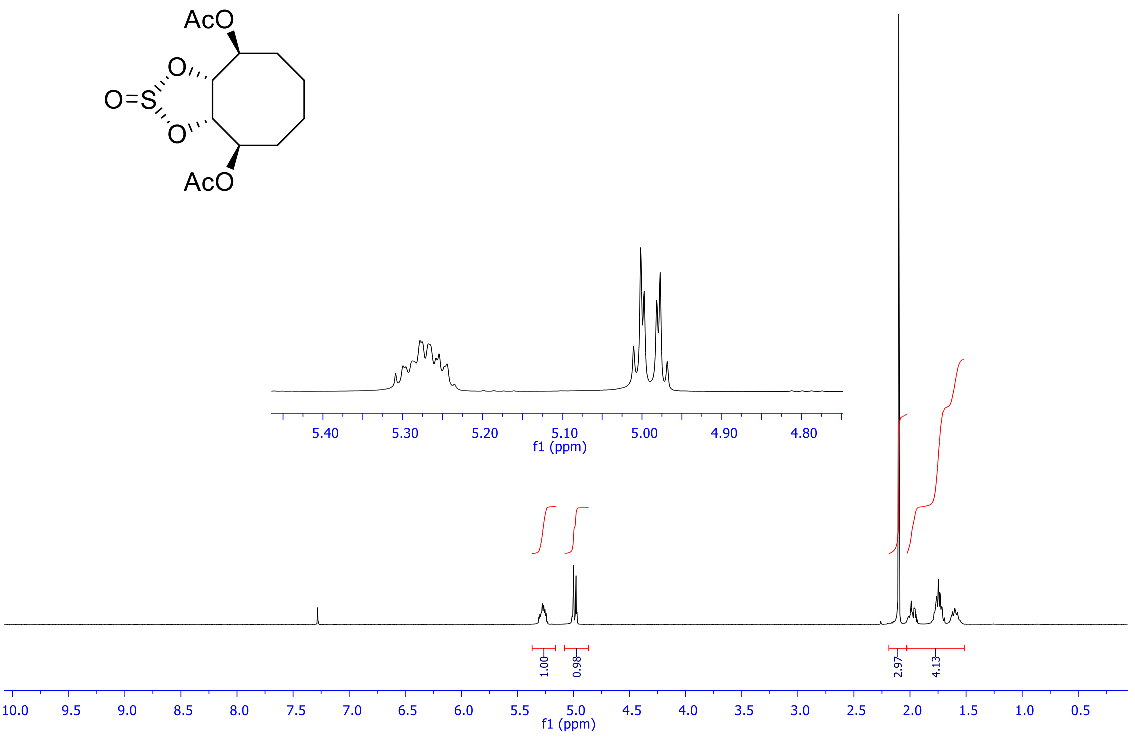

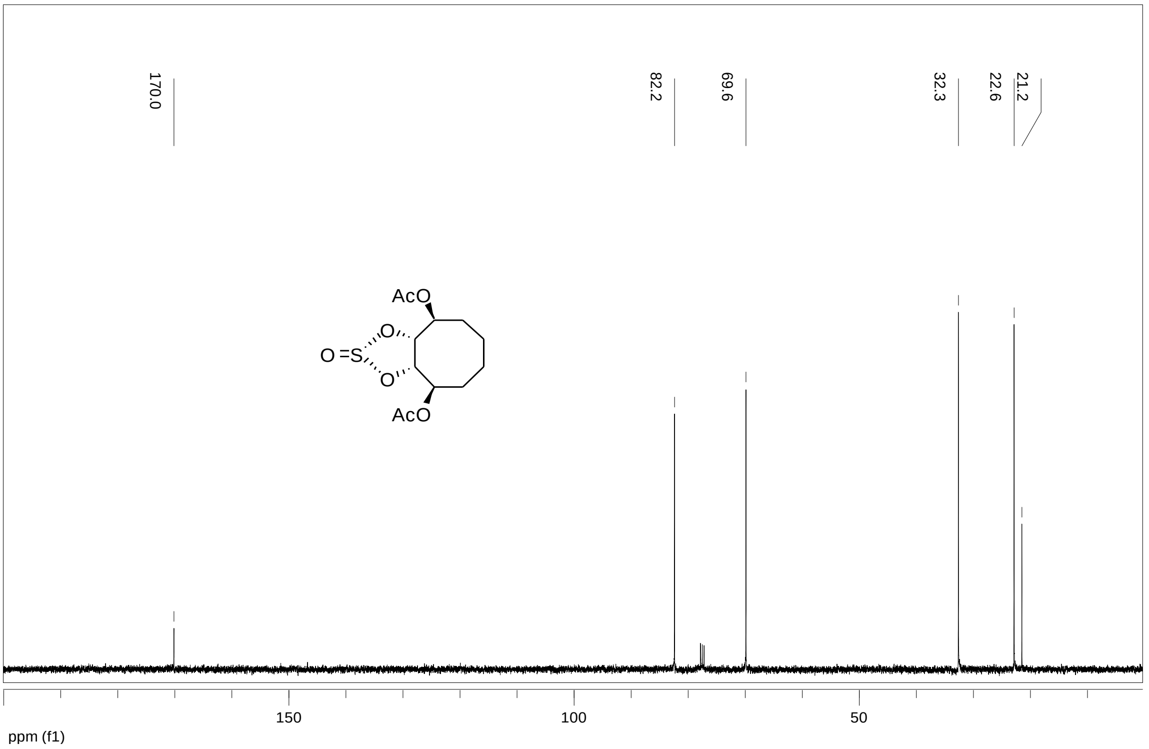

(2s,3a*R*(*S*),4*S*(*R*),9*R*(*S*),9a*S*(*R*))-2-Oxidooctahydrocycloocta[*d*][1,3,2]dioxathiole-4,9-diyl diacetate (8): $CDCl_3$-HMQC

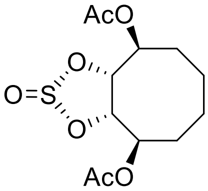

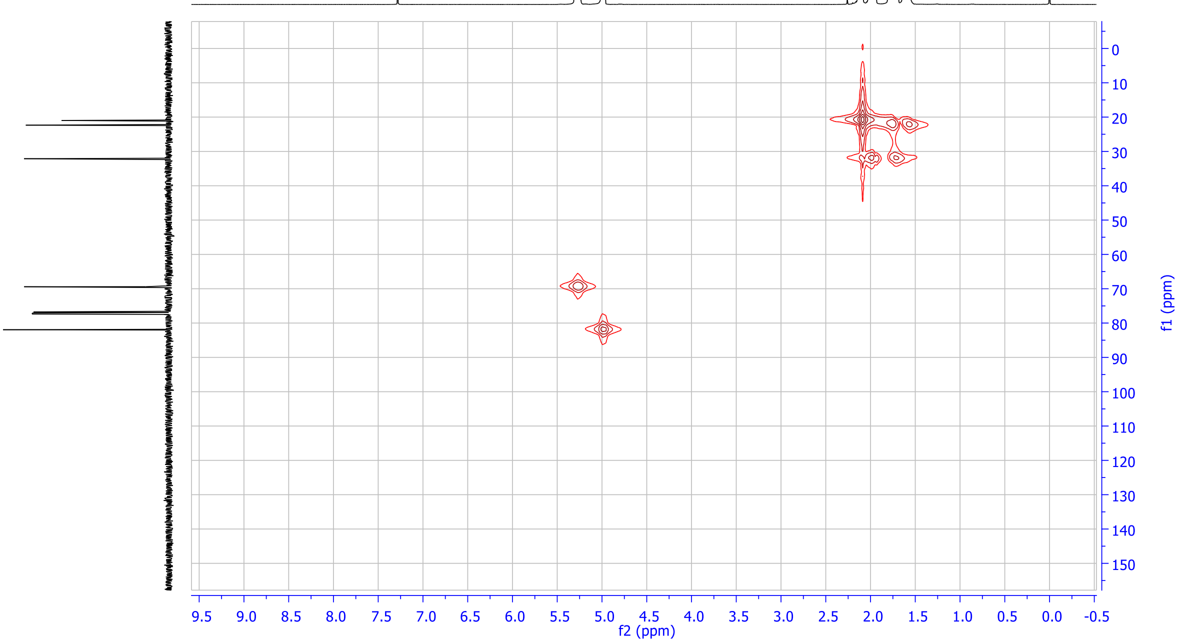

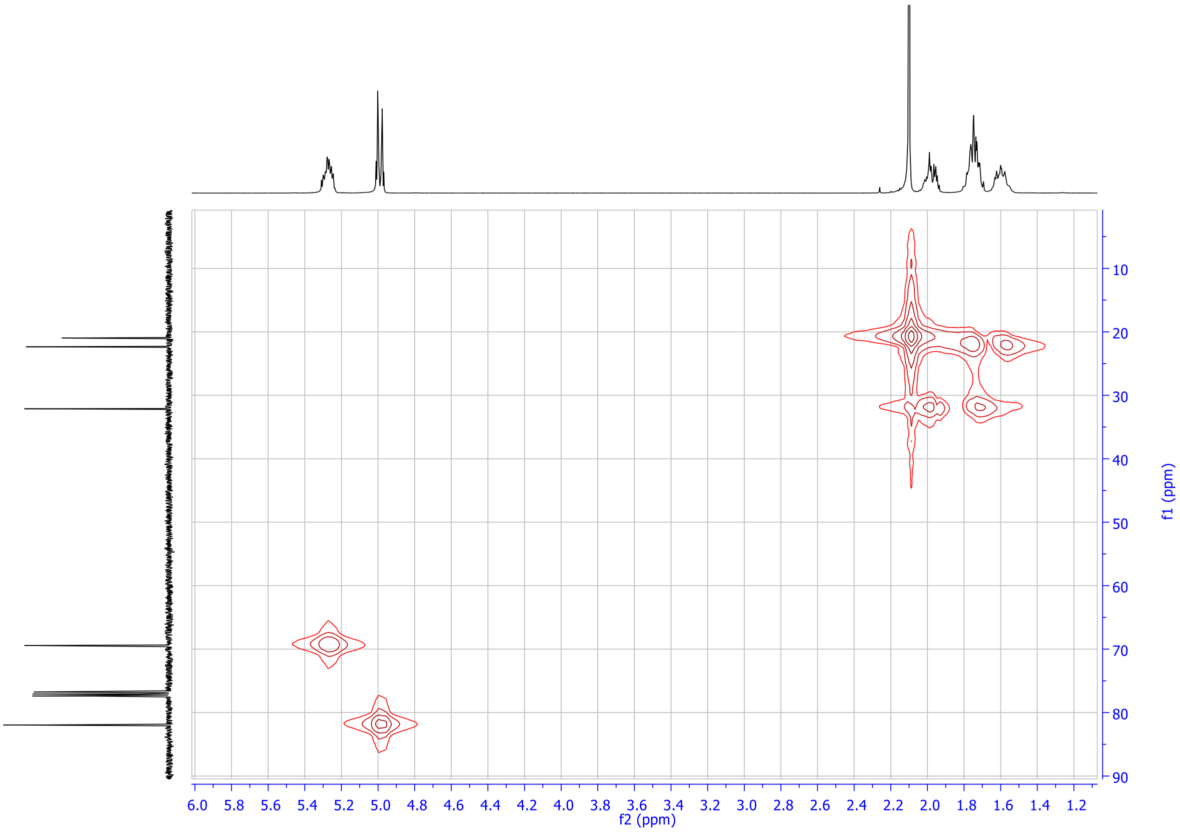

(2s,3a*R*(*S*),4*S*(*R*),9*R*(*S*),9a*S*(*R*))-2-Oxidooctahydrocycloocta[*d*][1,3,2]dioxathiole-4,9-diyl diacetate (8):CDCl$_3$-COSY

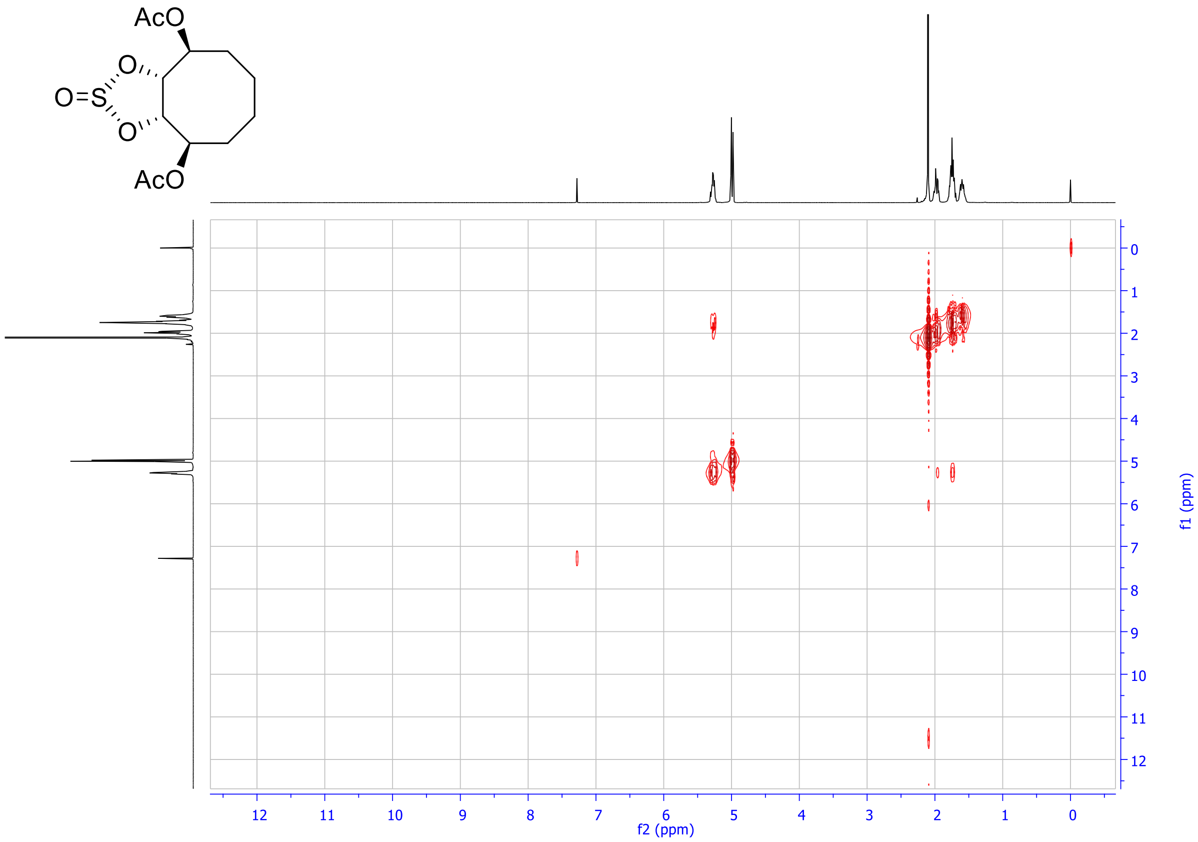

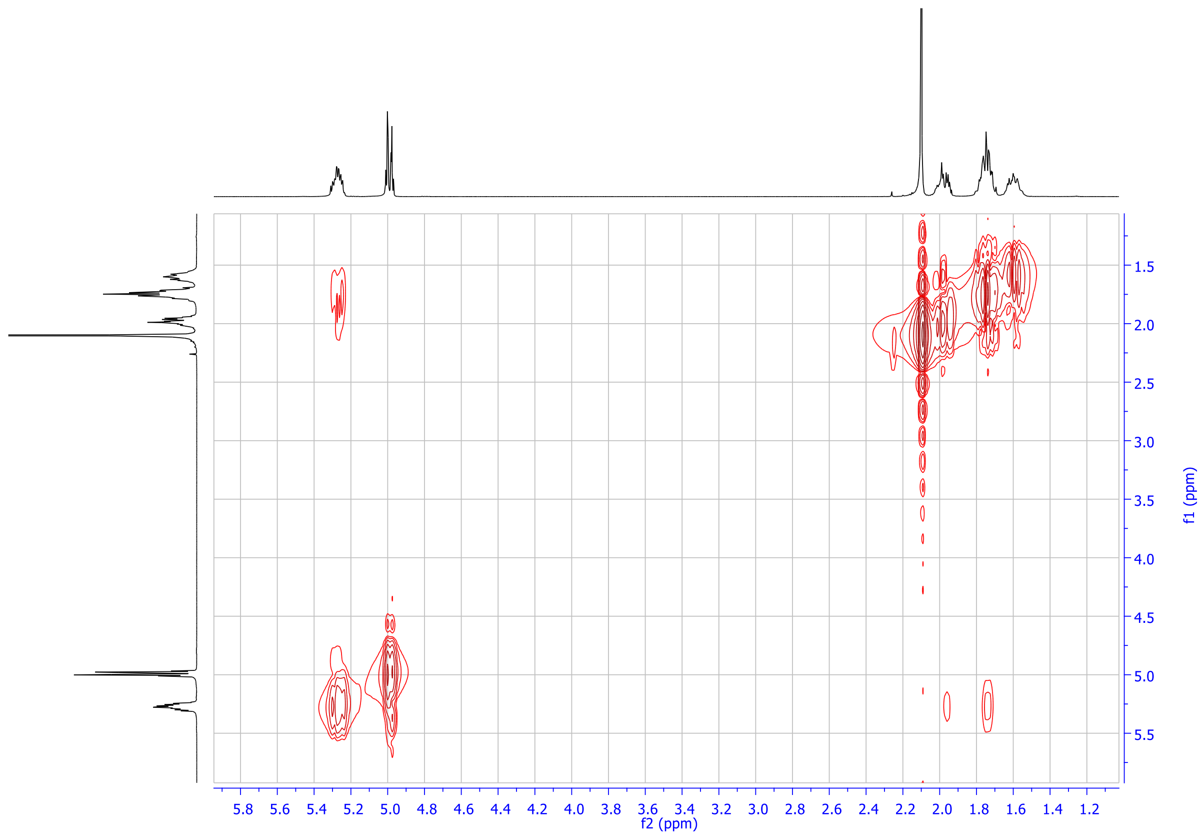

(3a*R*(*S*),4*S*(*R*),9*R*(*S*),9a*S*(*R*))-2,2-Dioxidooctahydrocycloocta[*d*][1,3,2]dioxathiole-4,9-diyl diacetate (9): $CDCl_3$ (^1H NMR and ^{13}C NMR)

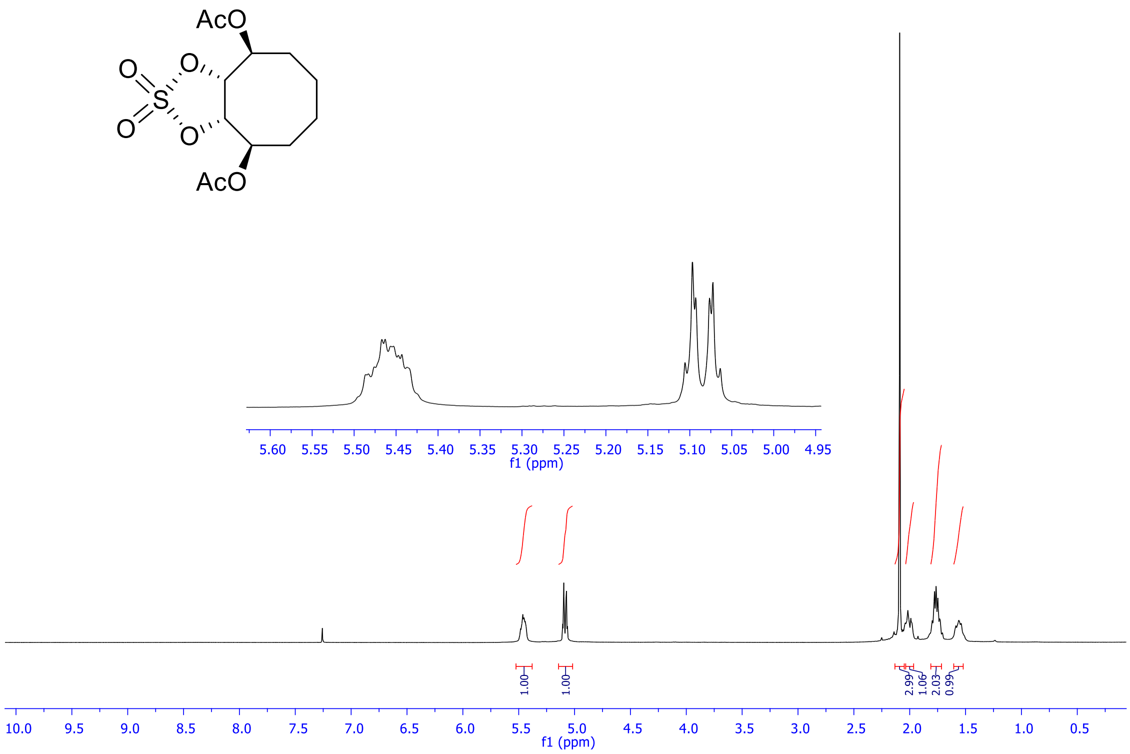

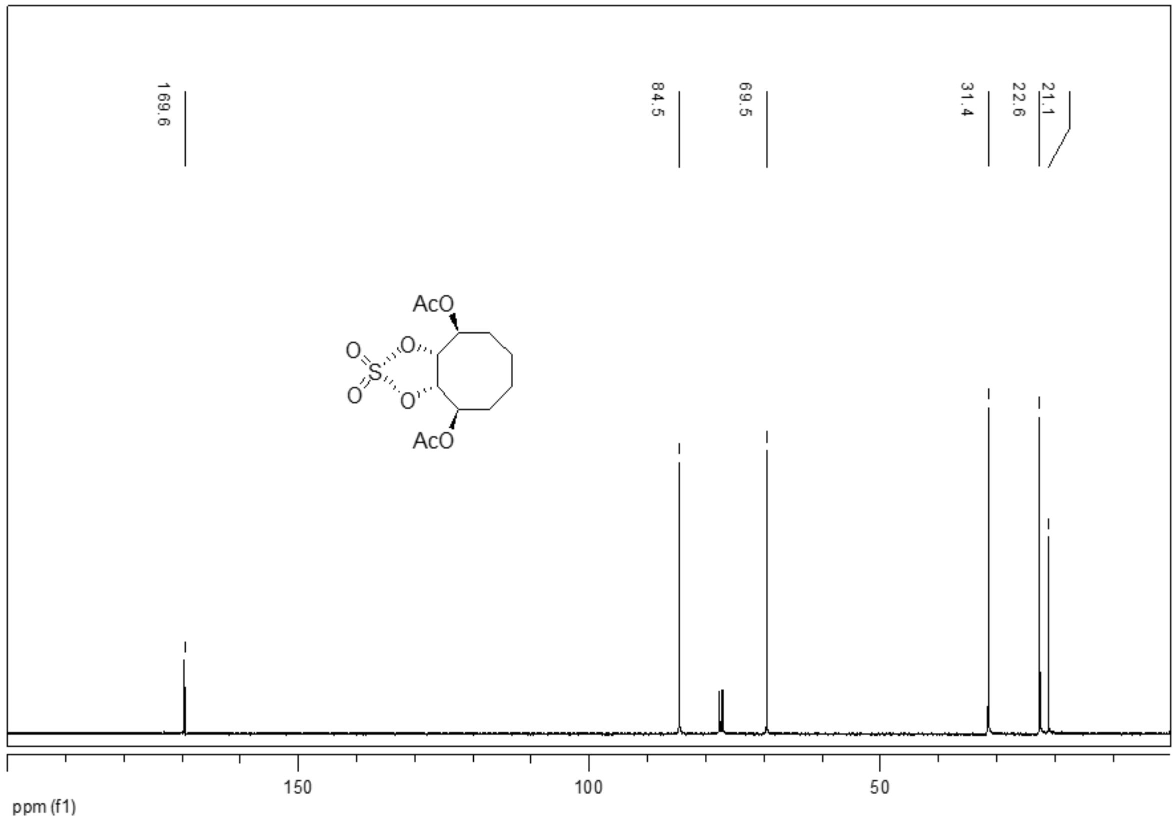

1*S*(*R*),2*S*(*R*),3*R*(*S*),4*R*(*S*))-3-Azidocyclooctane-1,2,4-triol (10): D_2O (^1H NMR and ^{13}C NMR)

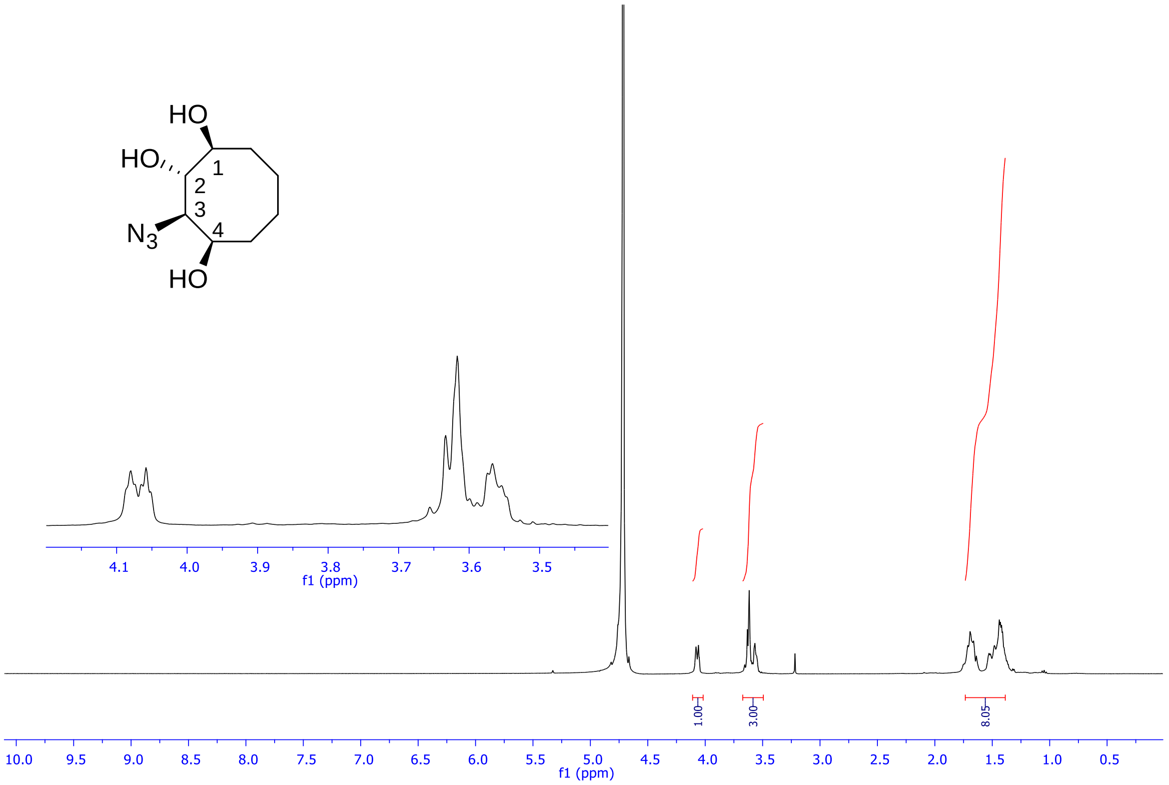

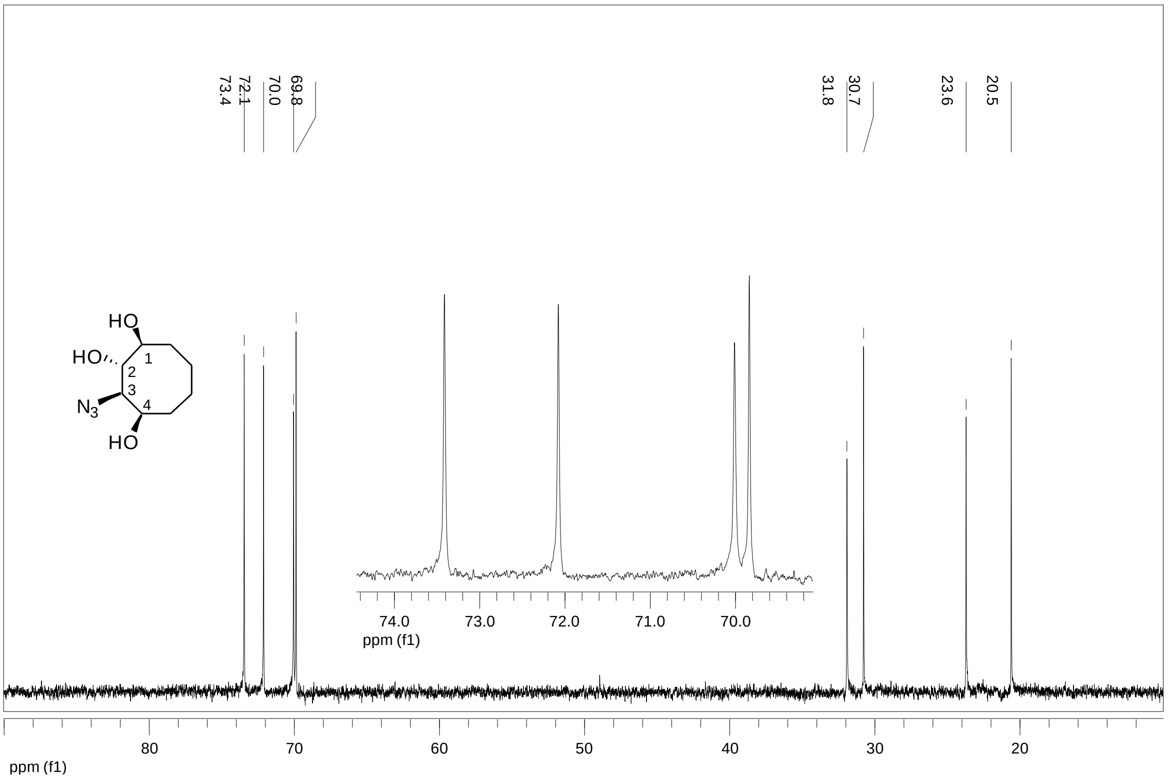

(1*S*(*R*),2*S*(*R*),3*R*(*S*),4*R*(*S*))-3-Azidocyclooctane-1,2,4-triyl triacetate (11): $CDCl_3$ (^{1}H NMR and Double Resonance)

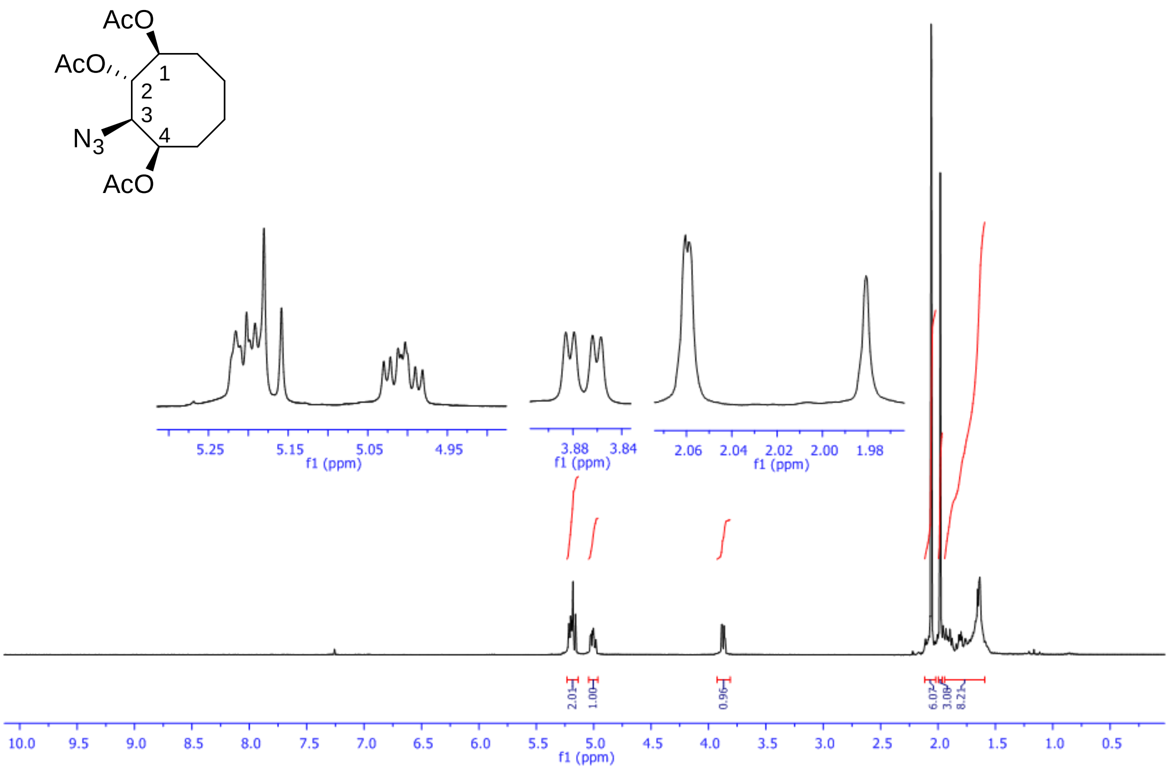

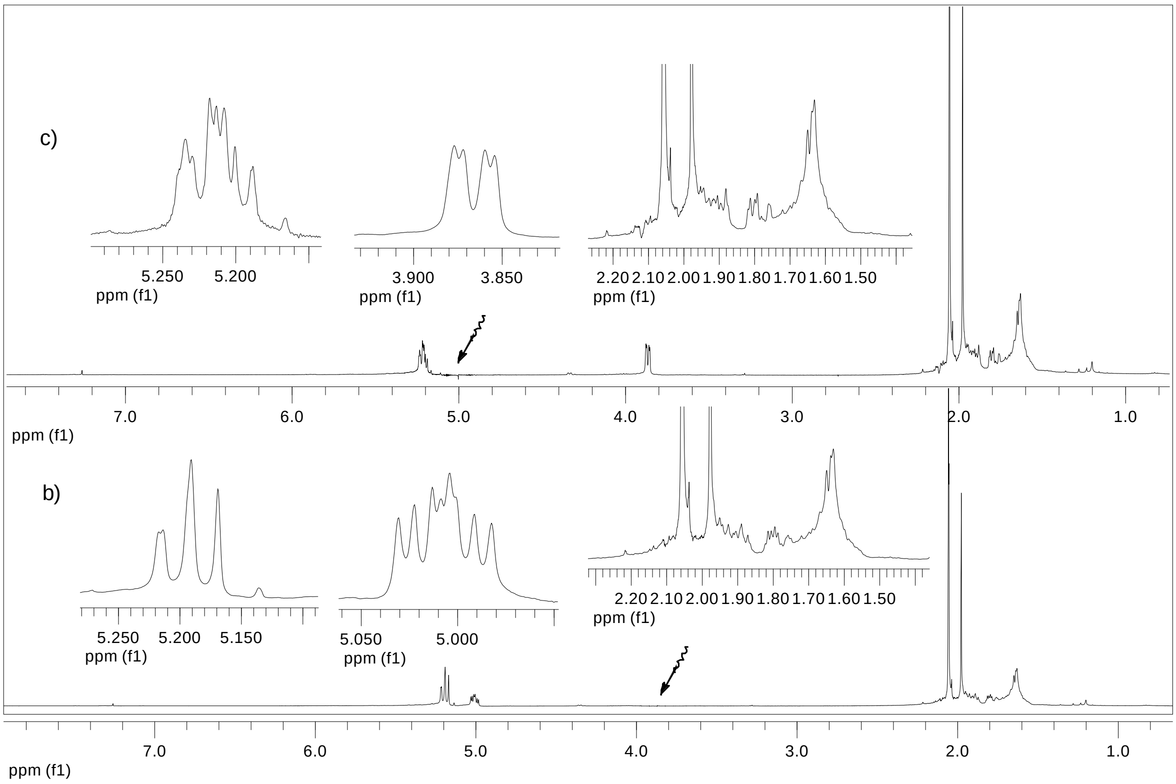

(1*S*(*R*),2*S*(*R*),3*R*(*S*),4*R*(*S*))-3-Azidocyclooctane-1,2,4-triyl triacetate (11): $CDCl_3$ (^{13}C NMR)

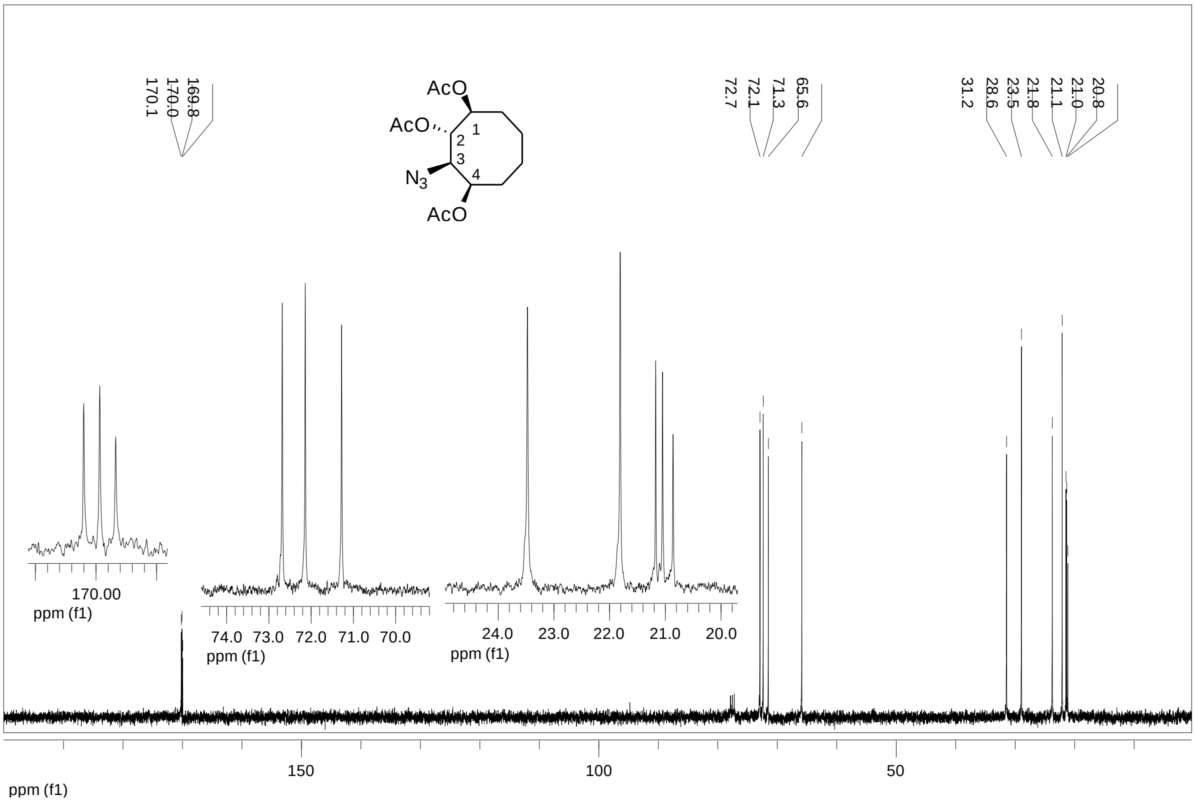

(1*S*(*R*),2*S*(*R*),3*R*(*S*),4*R*(*S*))-3-Azidocyclooctane-1,2,4-triyl triacetate (11): NOE-Dif spectra

a)

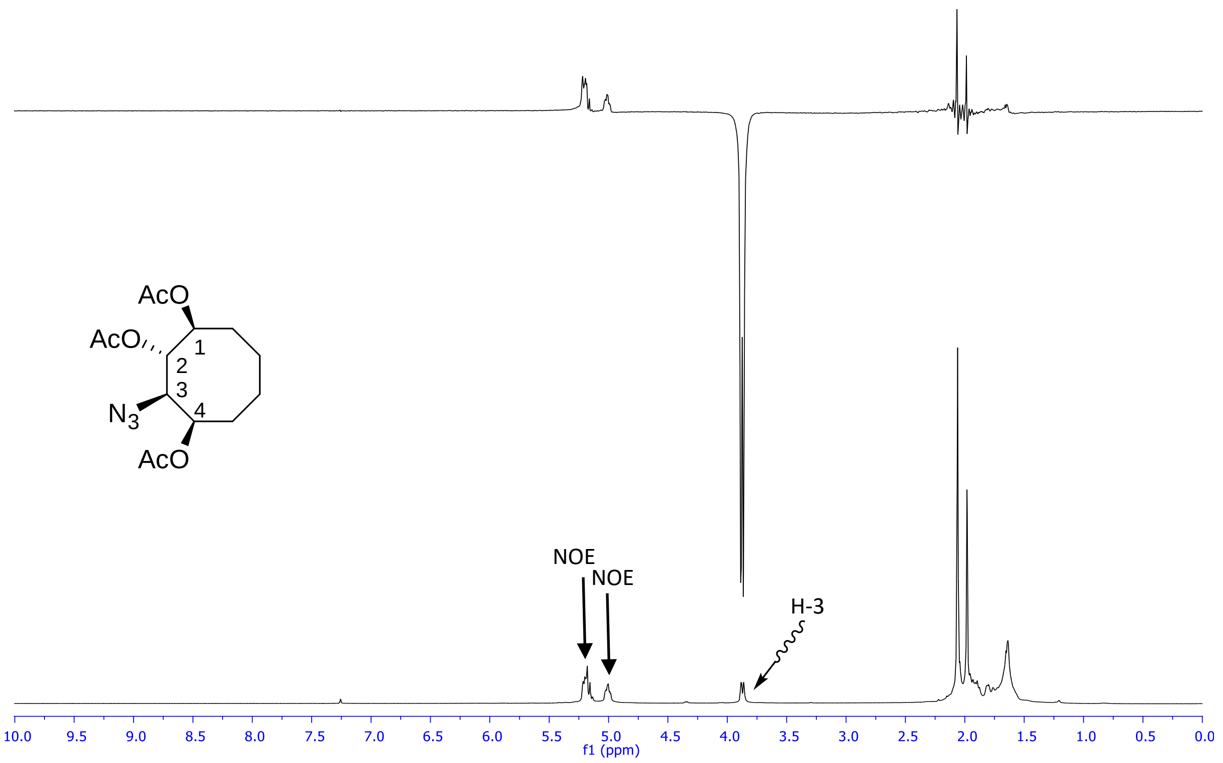

b)

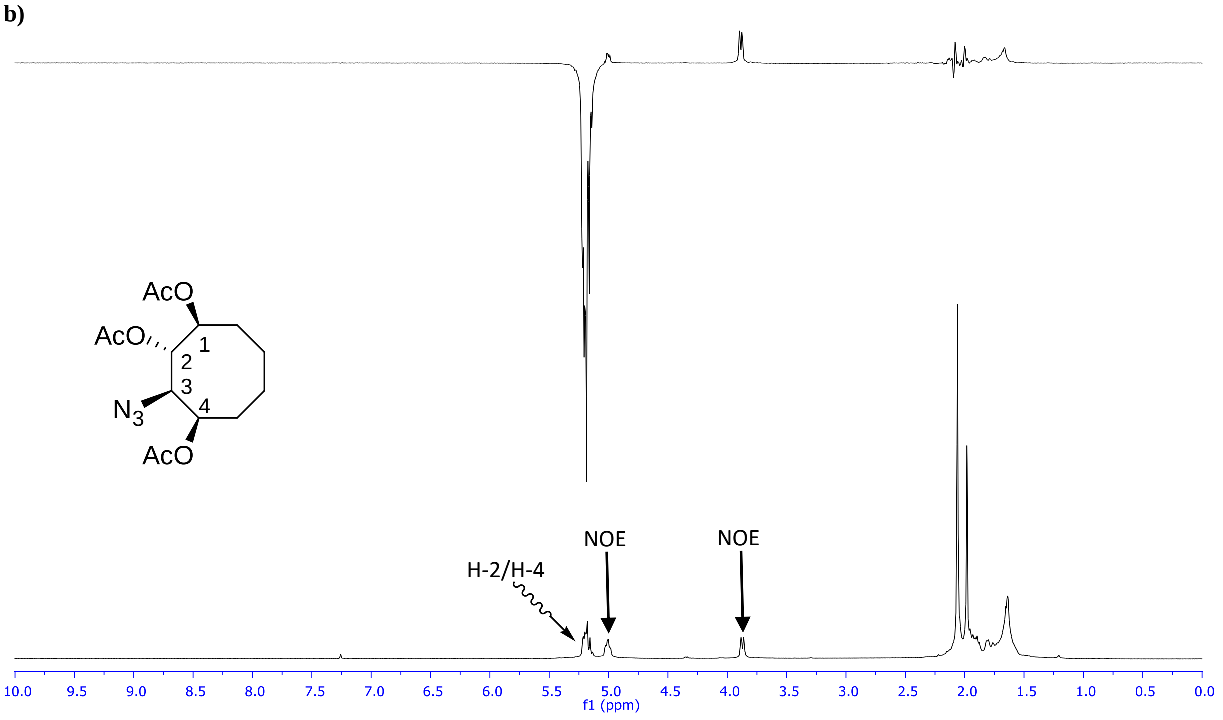

c)

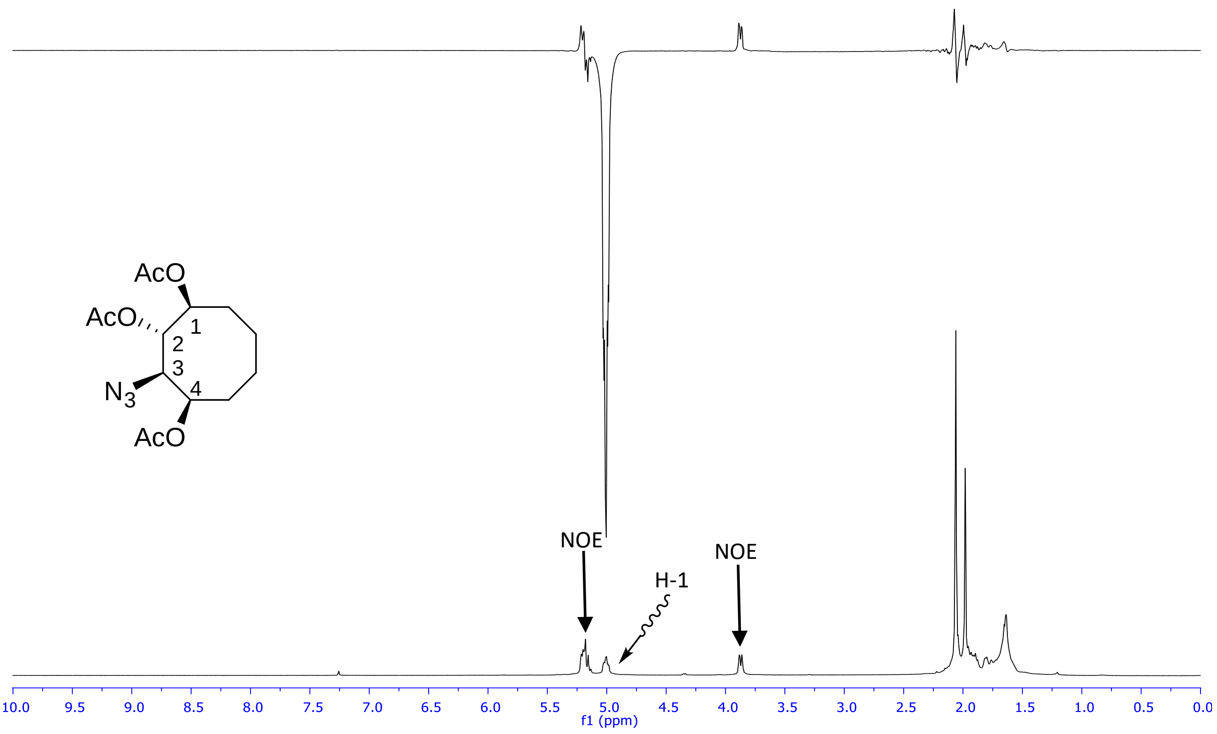

AcO
AcO
1
2
3
N3
4
AcO
NOE
H-1
NOE
10.0 9.5 9.0 8.5 8.0 7.5 7.0 6.5 6.0 5.5 5.0 4.5 4.0 3.5 3.0 2.5 2.0 1.5 1.0 0.5 0.0
f1 (ppm)

(1*S*(*R*),2*S*(*R*),3*R*(*S*),4*R*(*S*))-3-Azidocyclooctane-1,2,4-triyl triacetate (11): HMQC

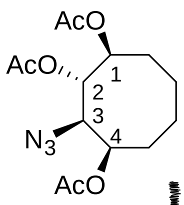

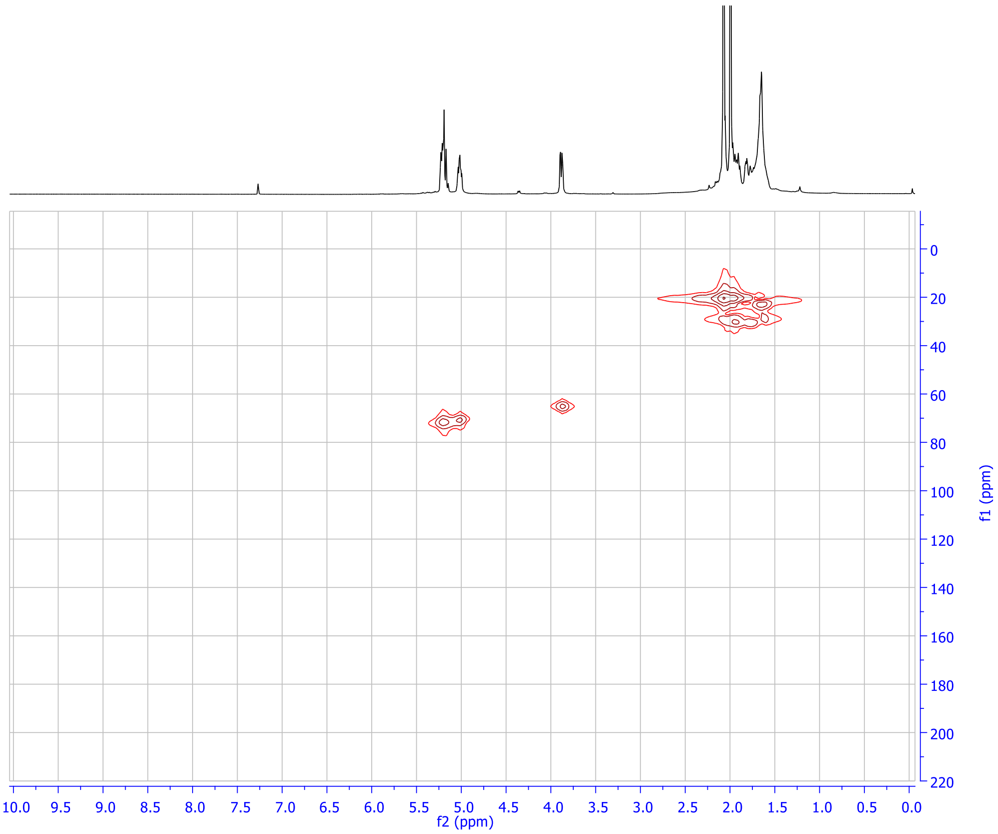

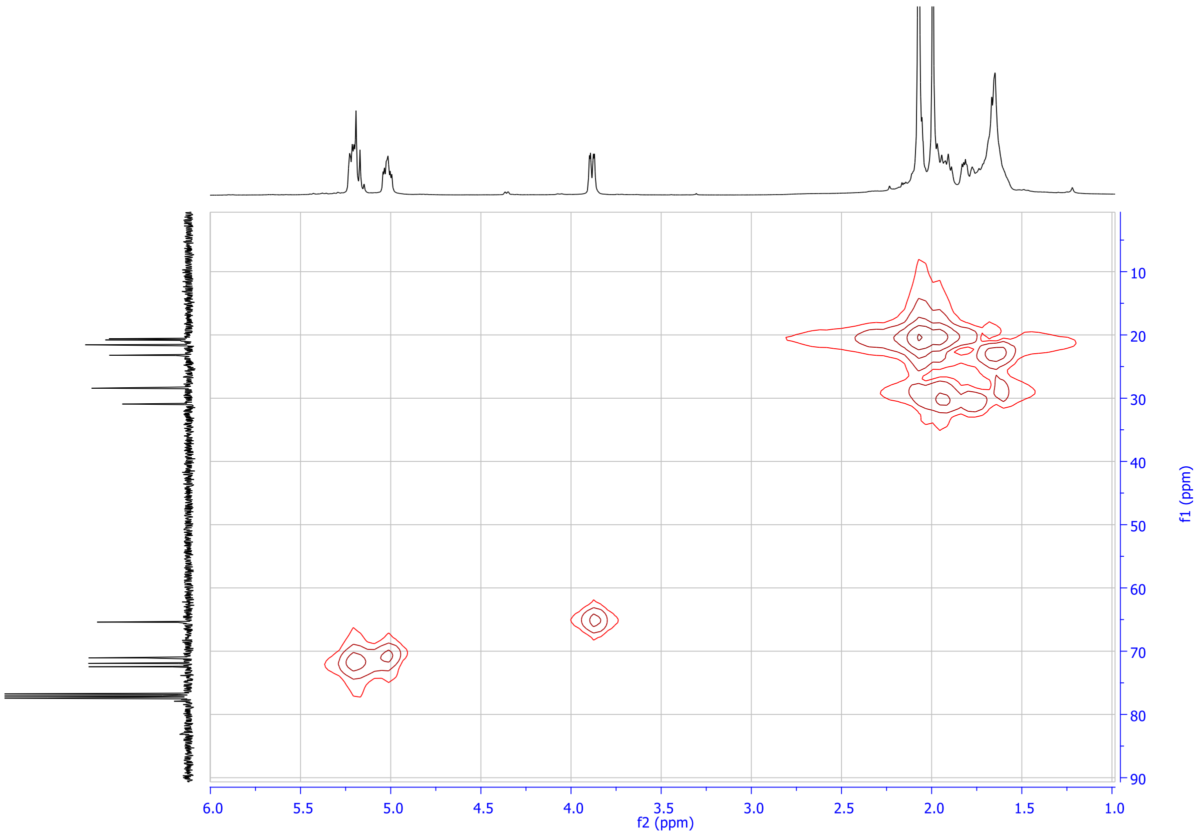

(1*S*(*R*),2*S*(*R*),3*R*(*S*),4*R*(*S*))-3-Azidocyclooctane-1,2,4-triyl triacetate (11): COSY

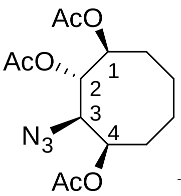

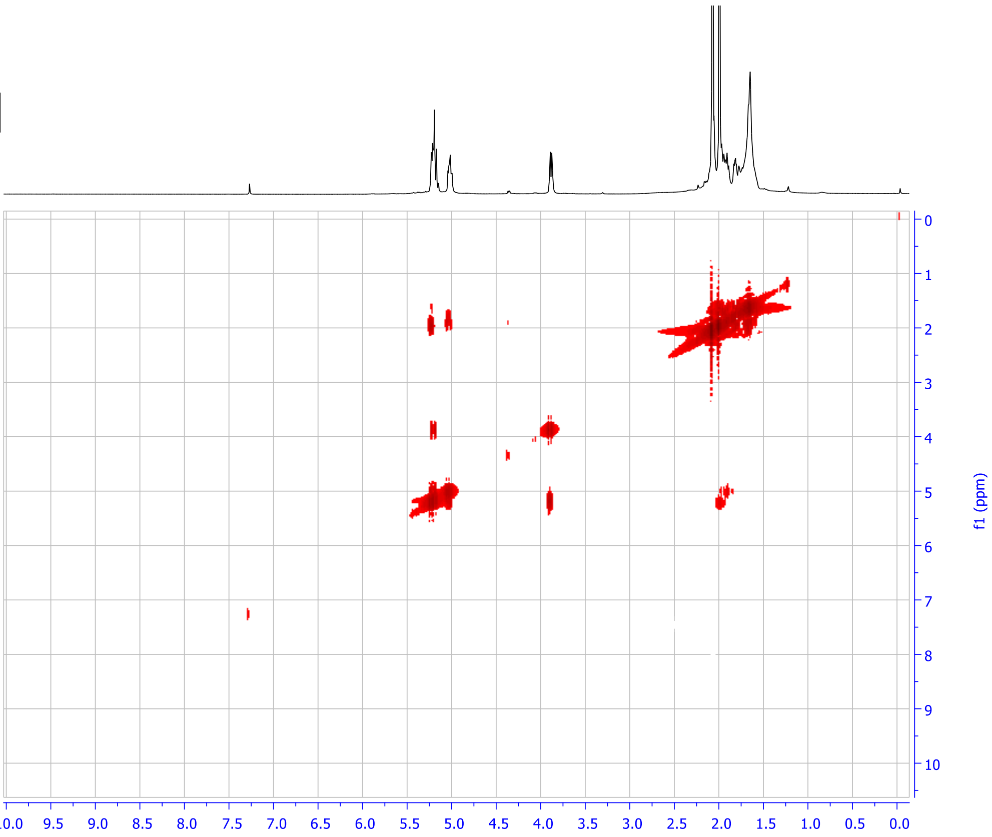

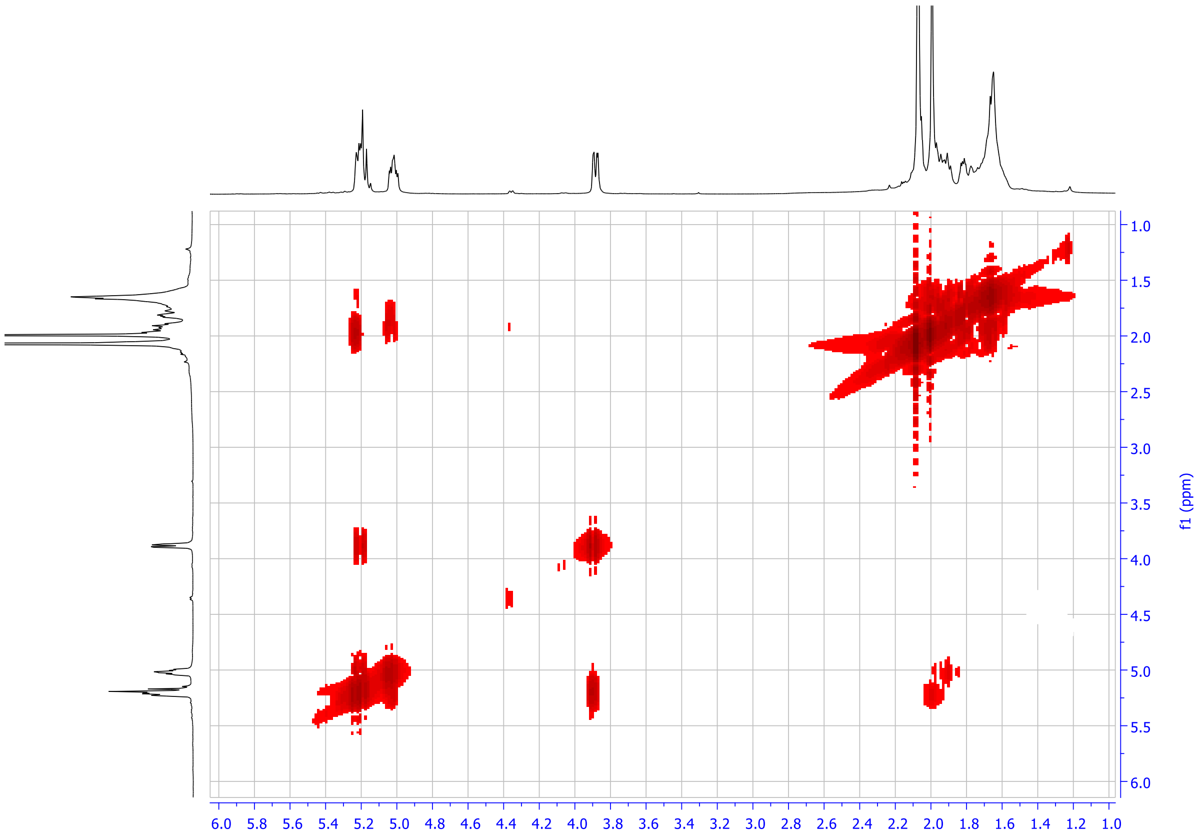

(1*S*(*R*),2*S*(*R*),3*R*(*S*),4*R*(*S*))-3-Aminocyclooctane-1,2,4-triol (12): CD_3OD (^1H NMR and ^{13}C NMR)

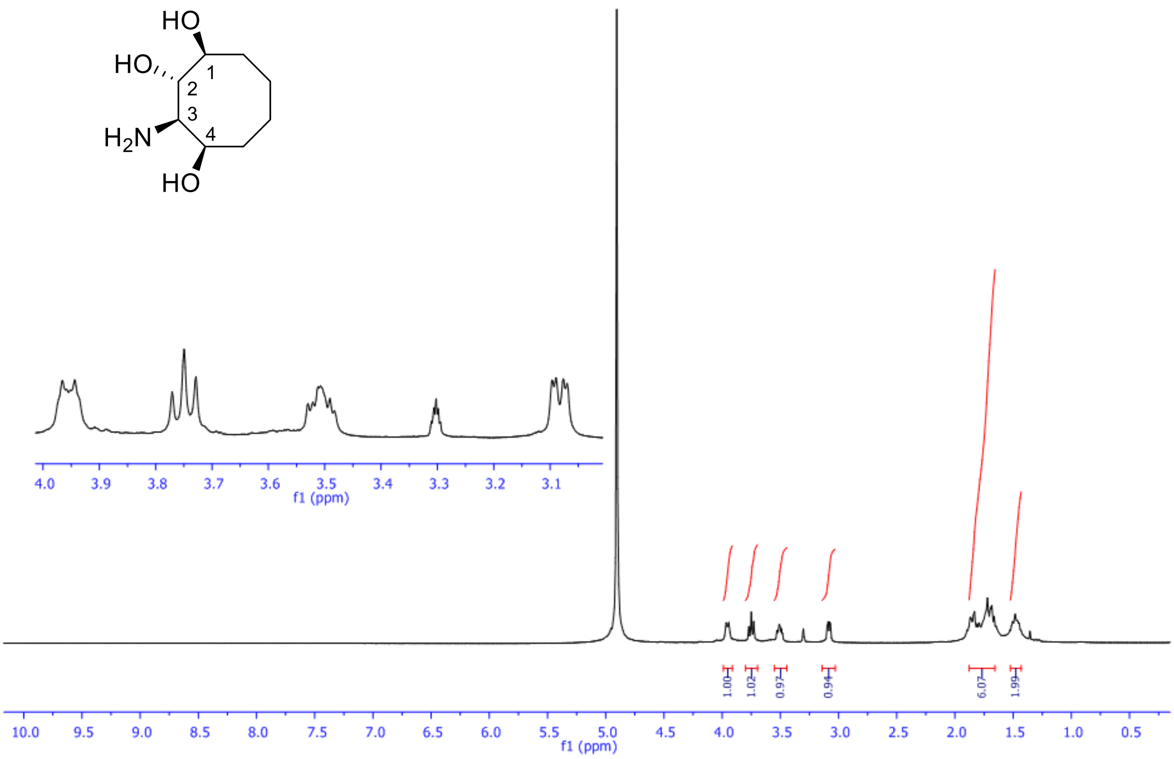

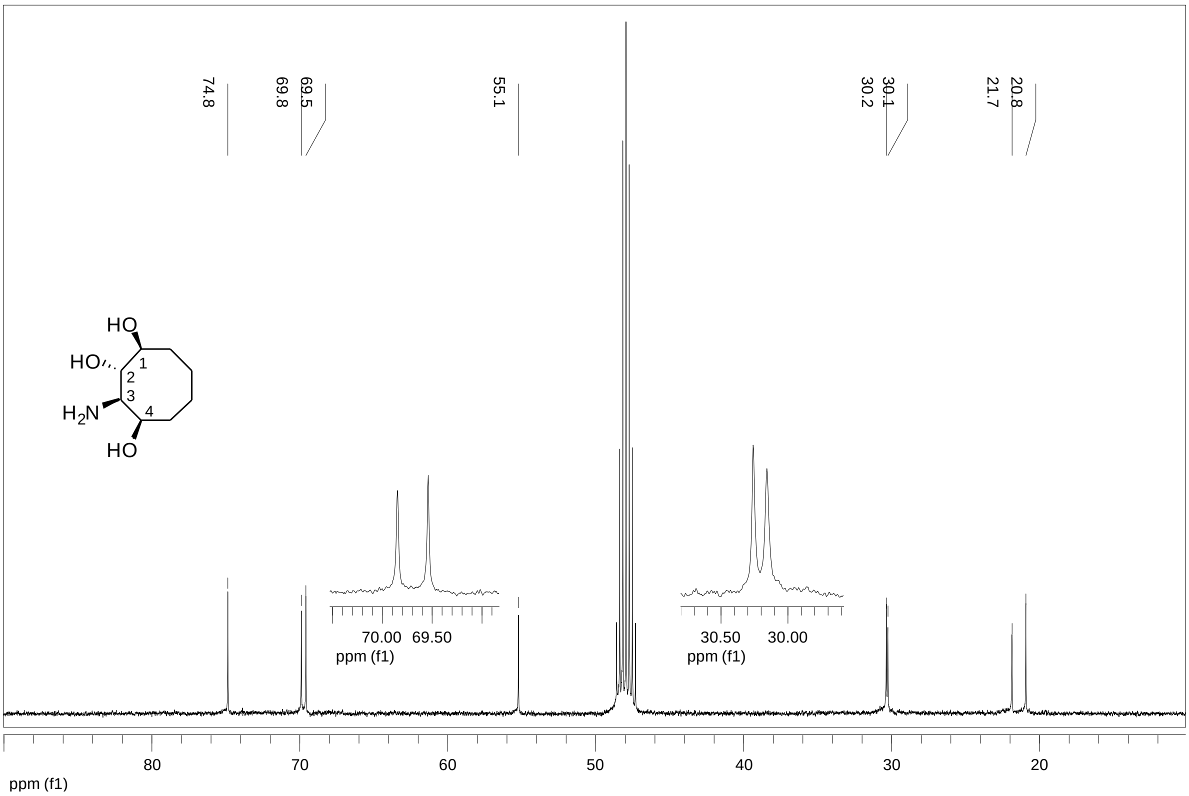

(1*S*(*R*),2*R*(*S*),3*R*(*S*),4*R*(*S*))-3-Bromocyclooctane-1,2,4-triol (14): CD_3OD (^1H NMR and ^{13}C NMR)

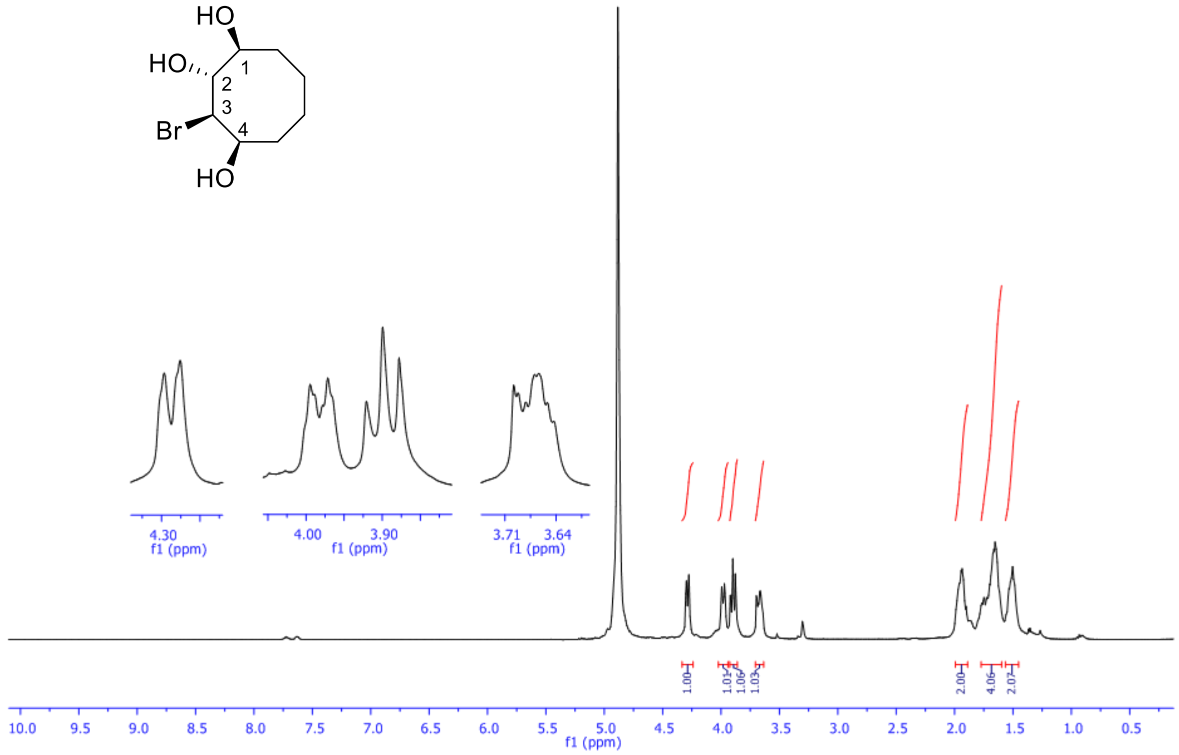

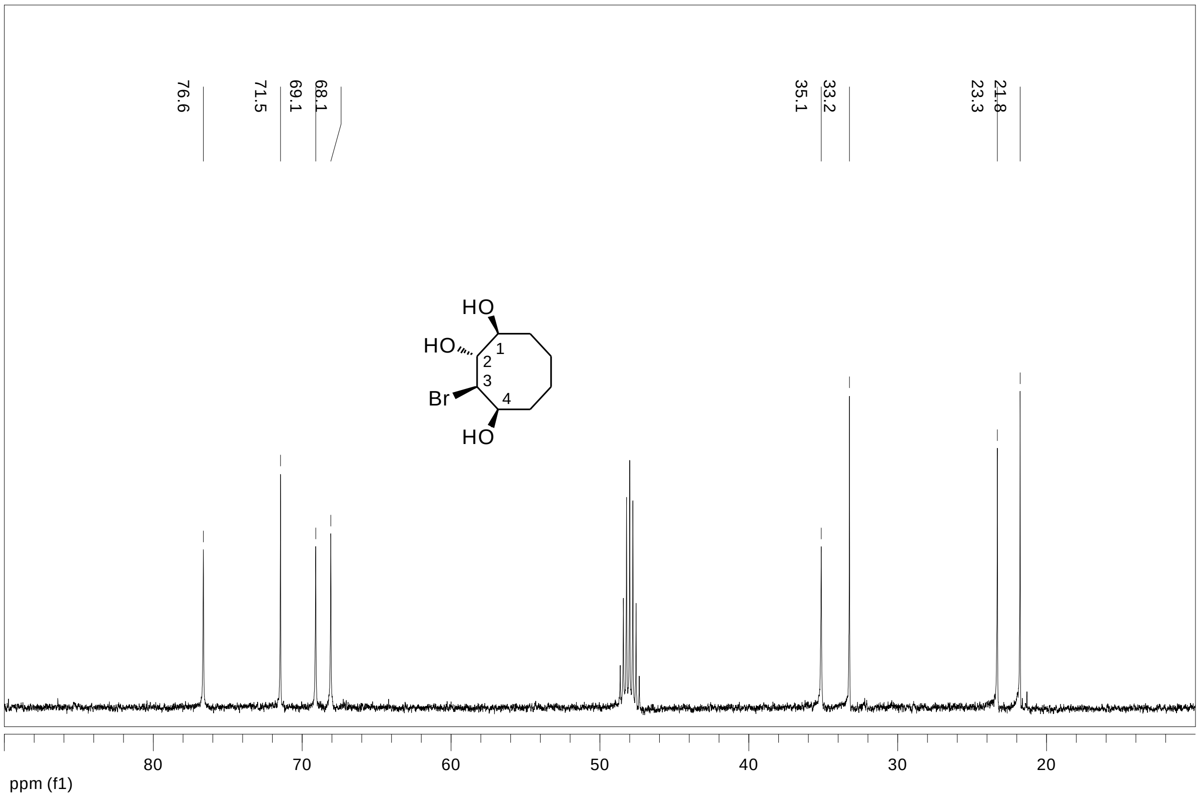

(1*S*(*R*),2*R*(*S*),3*R*(*S*),4*R*(*S*))-3-Bromocyclooctane-1,2,4-triyl triacetate (15): $CDCl_3$ (^1H NMR and double resonance)

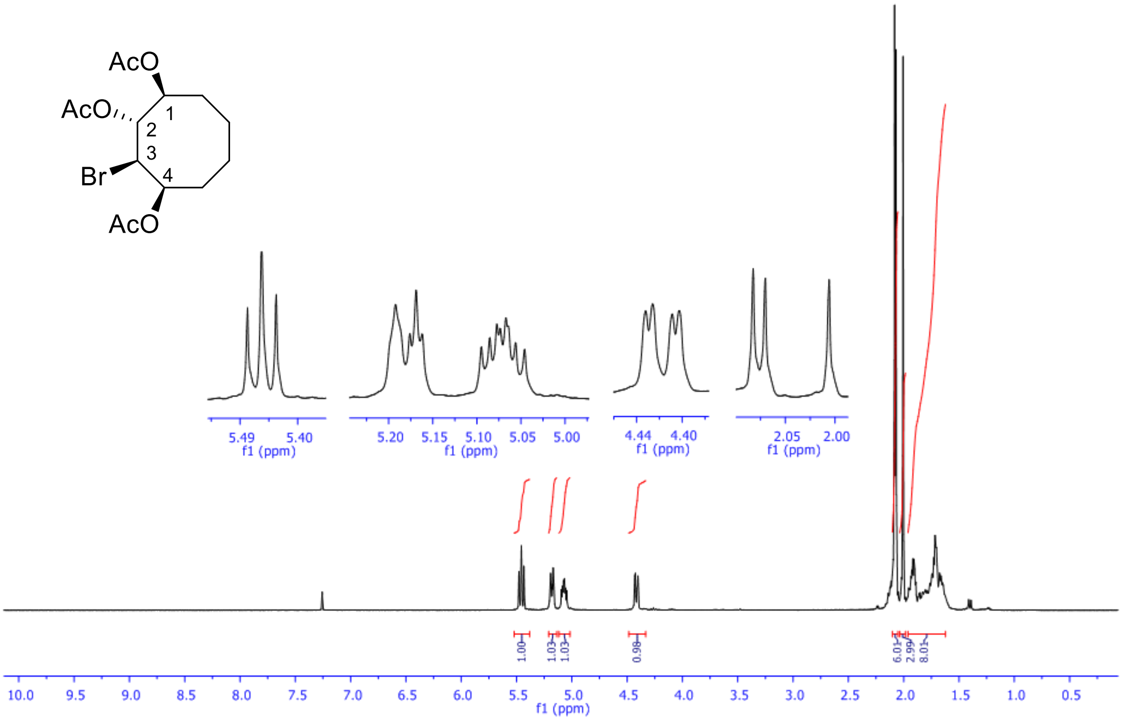

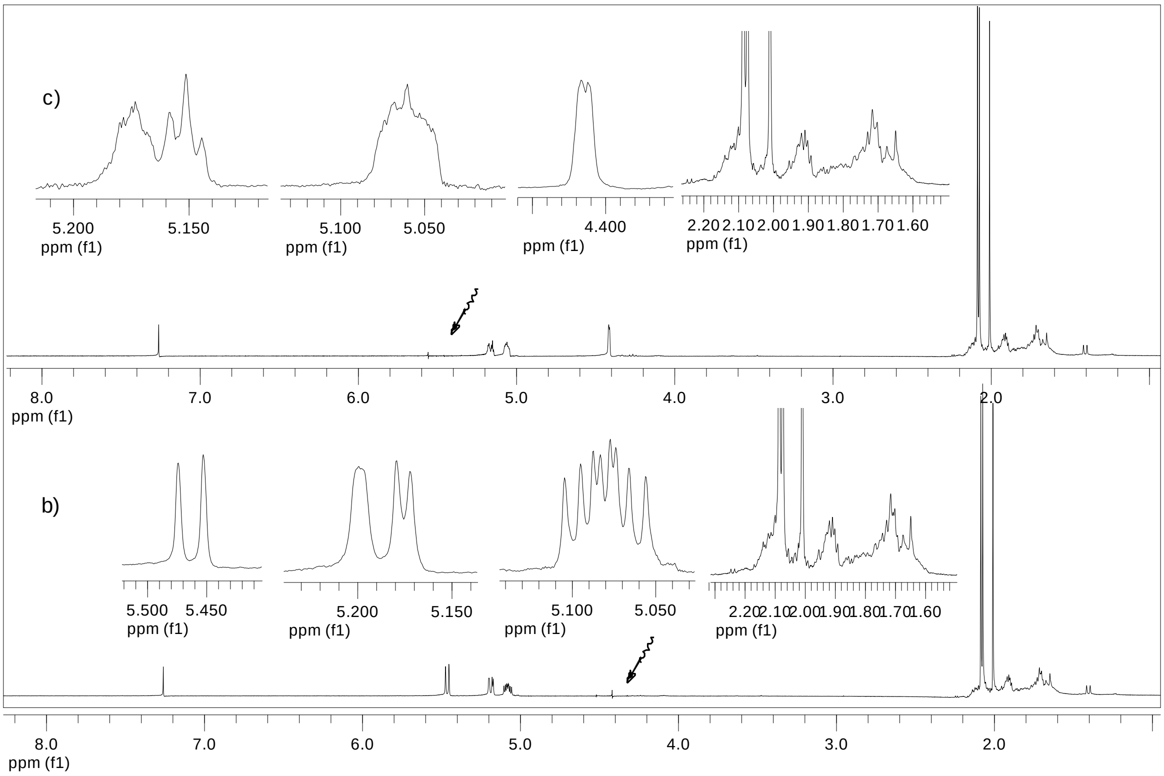

(1*S*(*R*),2*R*(*S*),3*R*(*S*),4*R*(*S*))-3-Bromocyclooctane-1,2,4-triyl triacetate (15): $CDCl_3$ (^{13}C NMR)

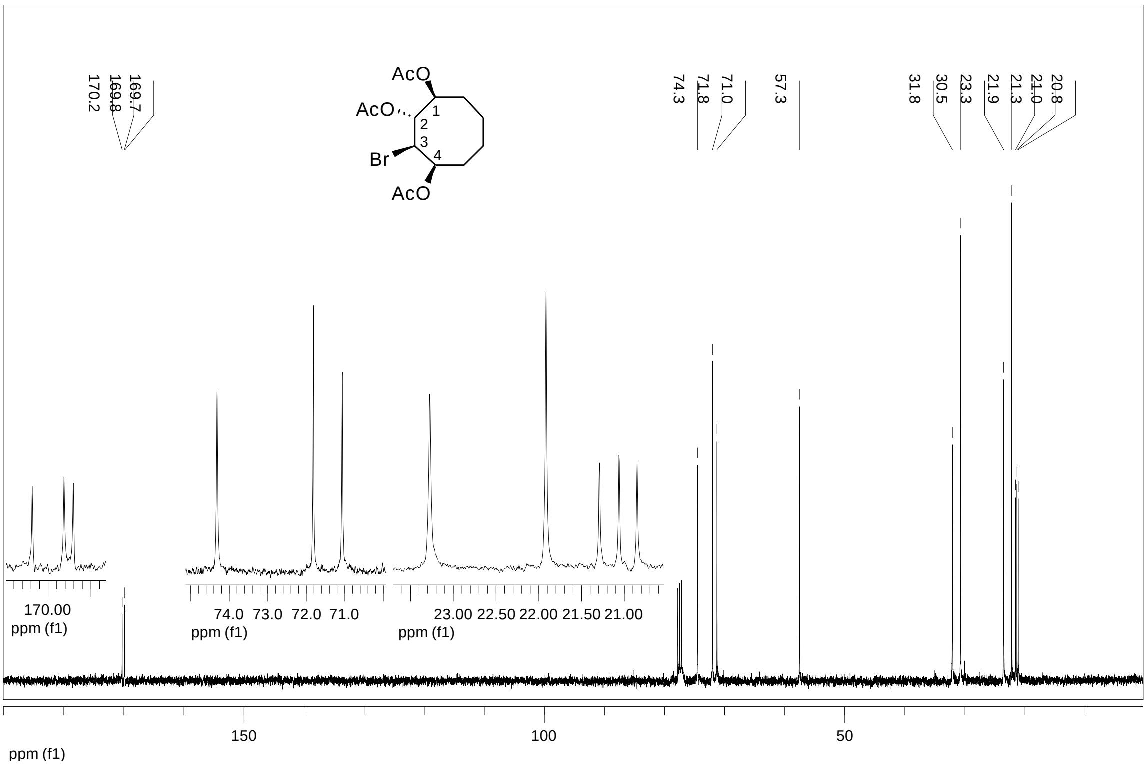

(1*S*(*R*),2*S*(*R*),3*S*(*R*),4*R*(*S*))-3-Azidocyclooctane-1,2,4-triol (16): D_2O (^1H NMR and ^{13}C NMR)

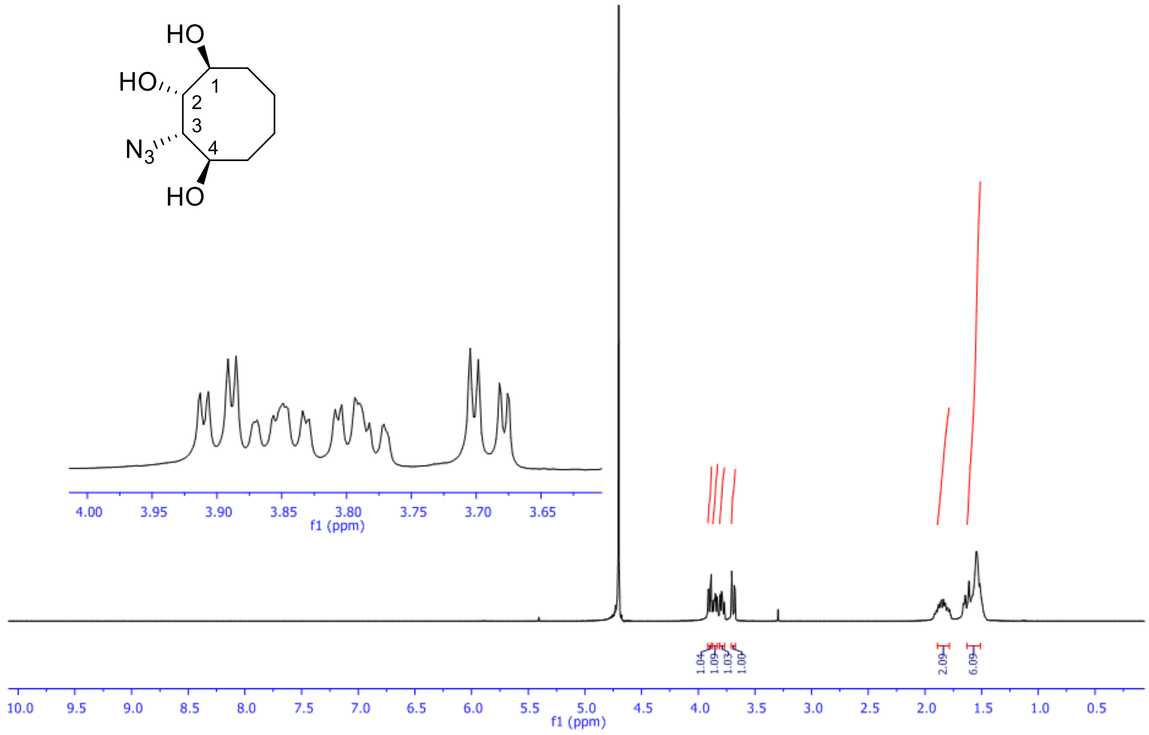

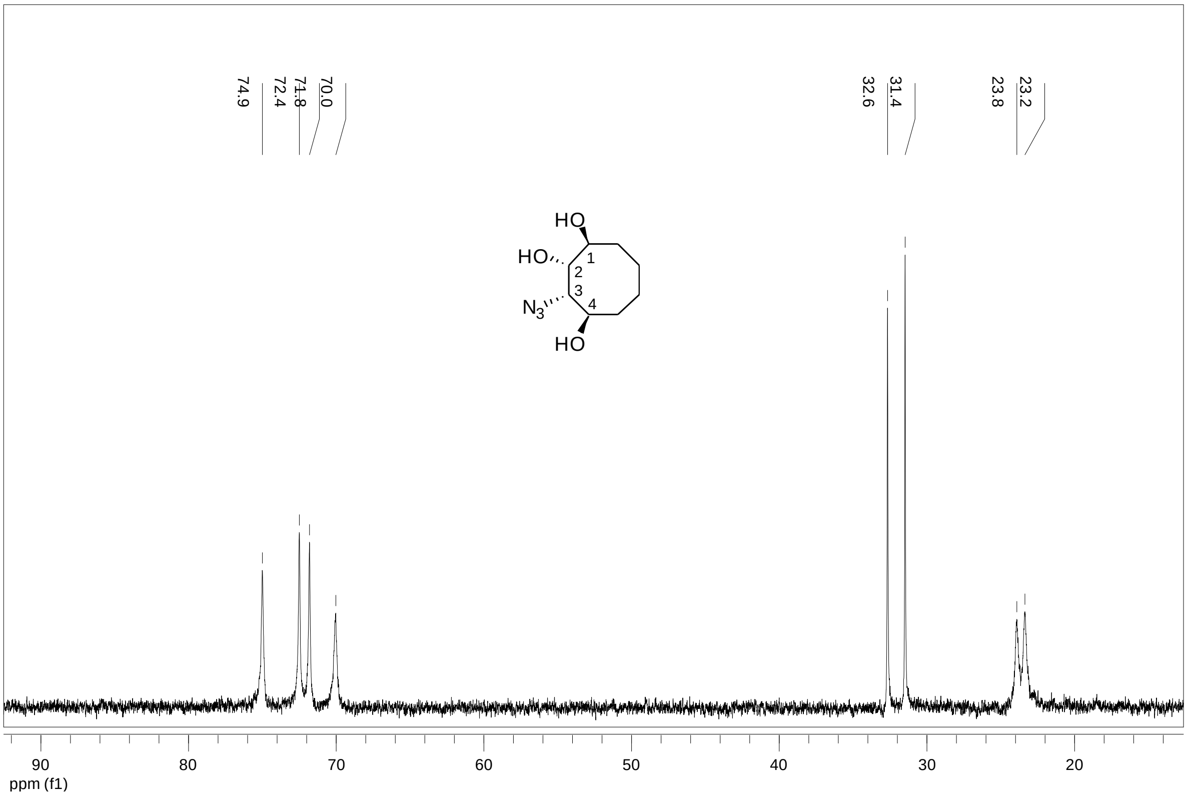

(1*S*(*R*),2*S*(*R*),3*S*(*R*),4*R*(*S*))-3-Azidocyclooctane-1,2,4-triyl triacetate (17): CDCl₃ (^{1}H NMR and double resonance)

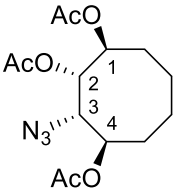

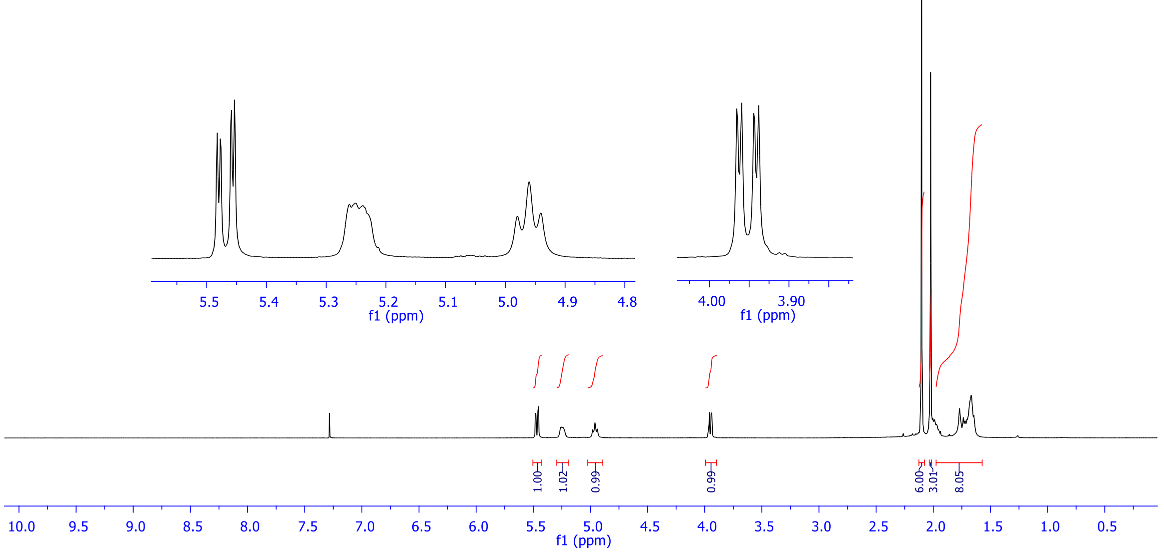

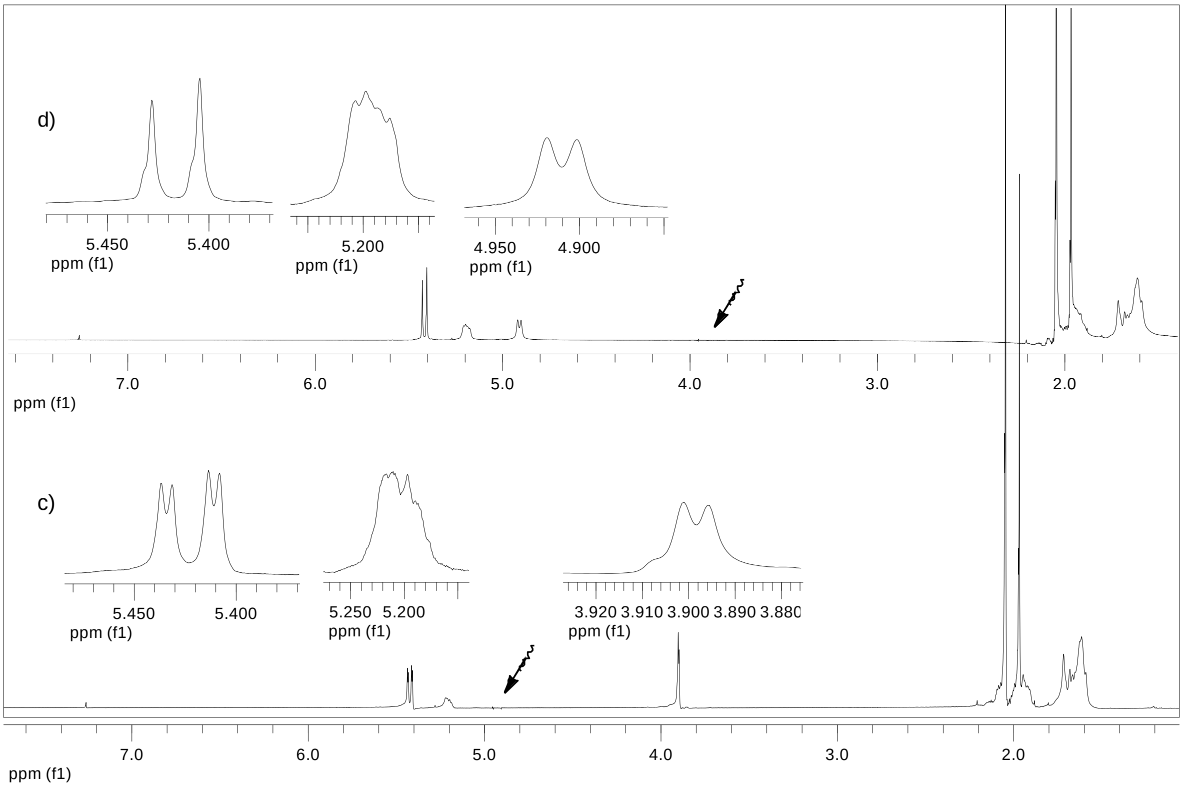

(1*S*(*R*),2*S*(*R*),3*S*(*R*),4*R*(*S*))-3-Azidocyclooctane-1,2,4-triyl triacetate (17): $CDCl_3$ (^{13}C NMR)

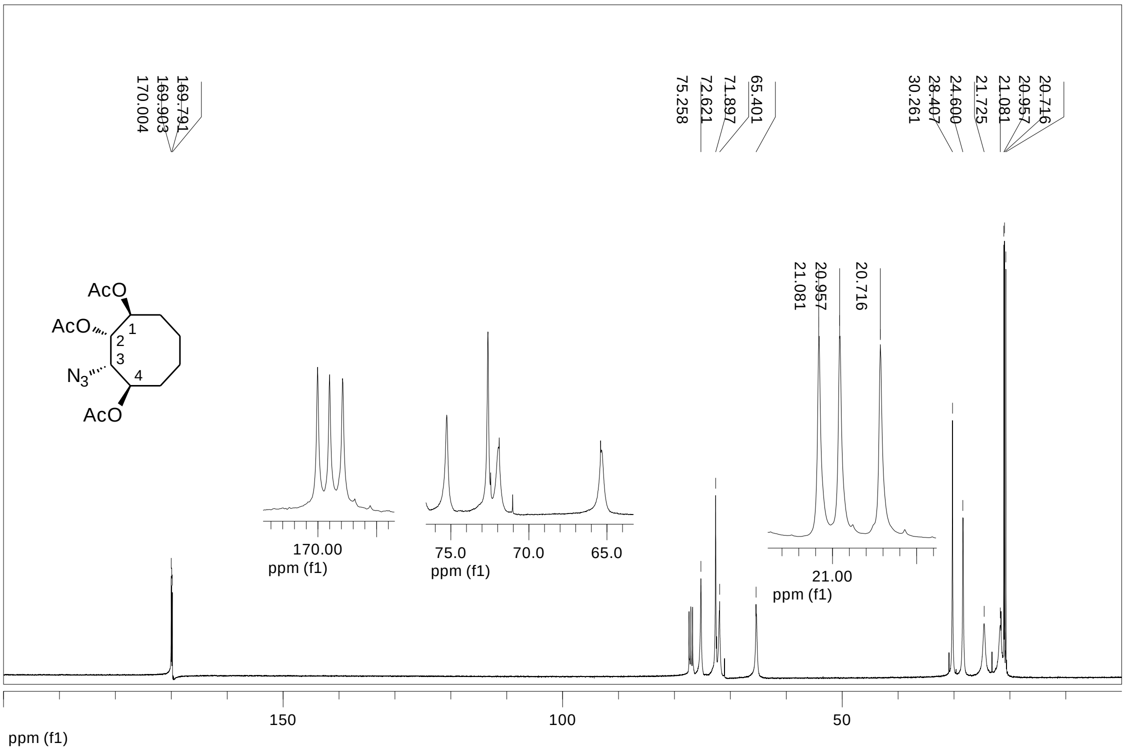

(1*S*(*R*),2*S*(*R*),3*S*(*R*),4*R*(*S*))-3-Azidocyclooctane-1,2,4-triyl triacetate (17): $CDCl_3$-HMQC

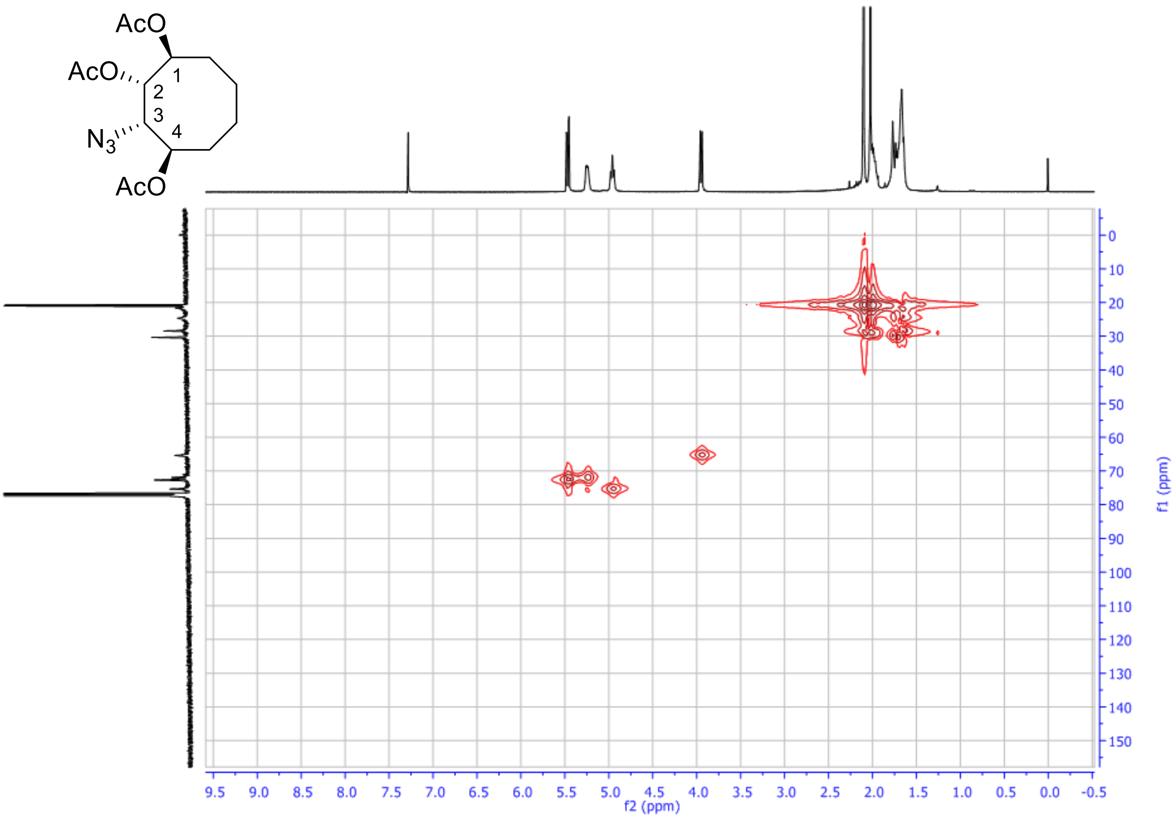

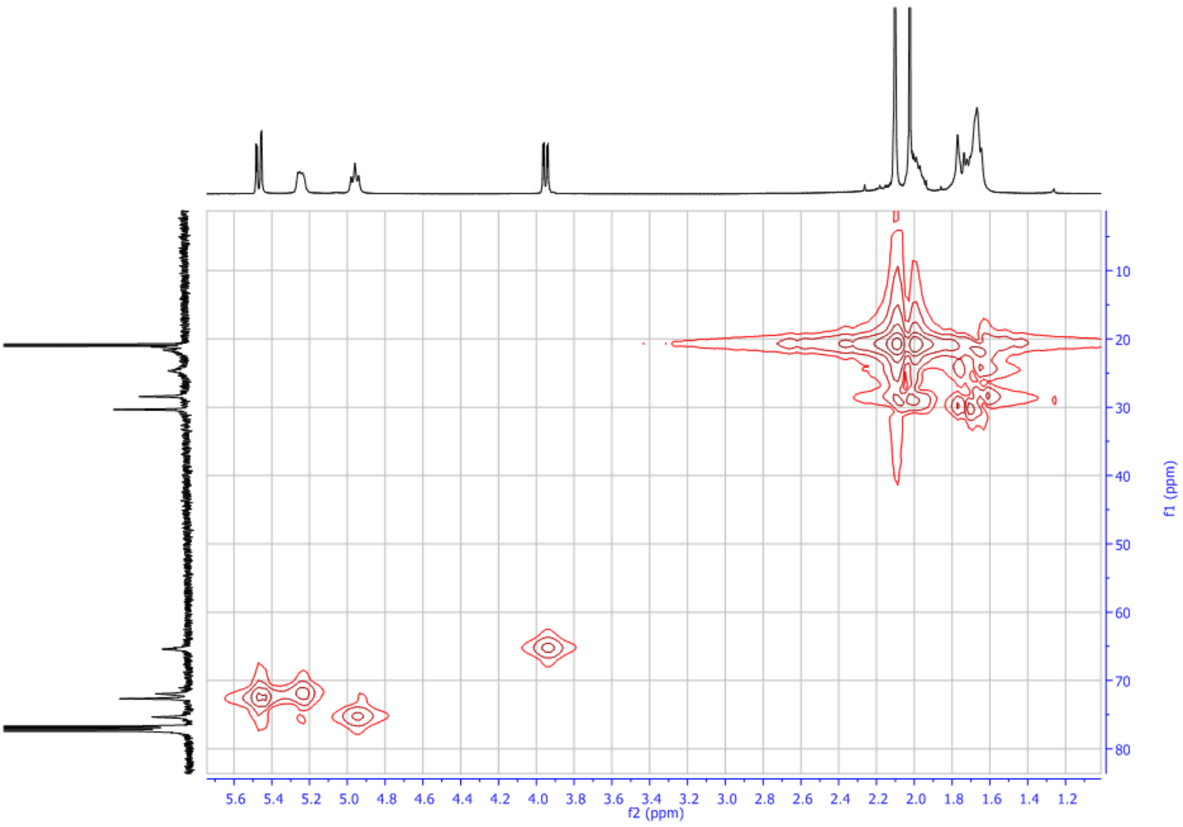

(1*S*(*R*),2*S*(*R*),3*S*(*R*),4*R*(*S*))-3-Azidocyclooctane-1,2,4-triyl triacetate (17): CDCl$_3$-COSY

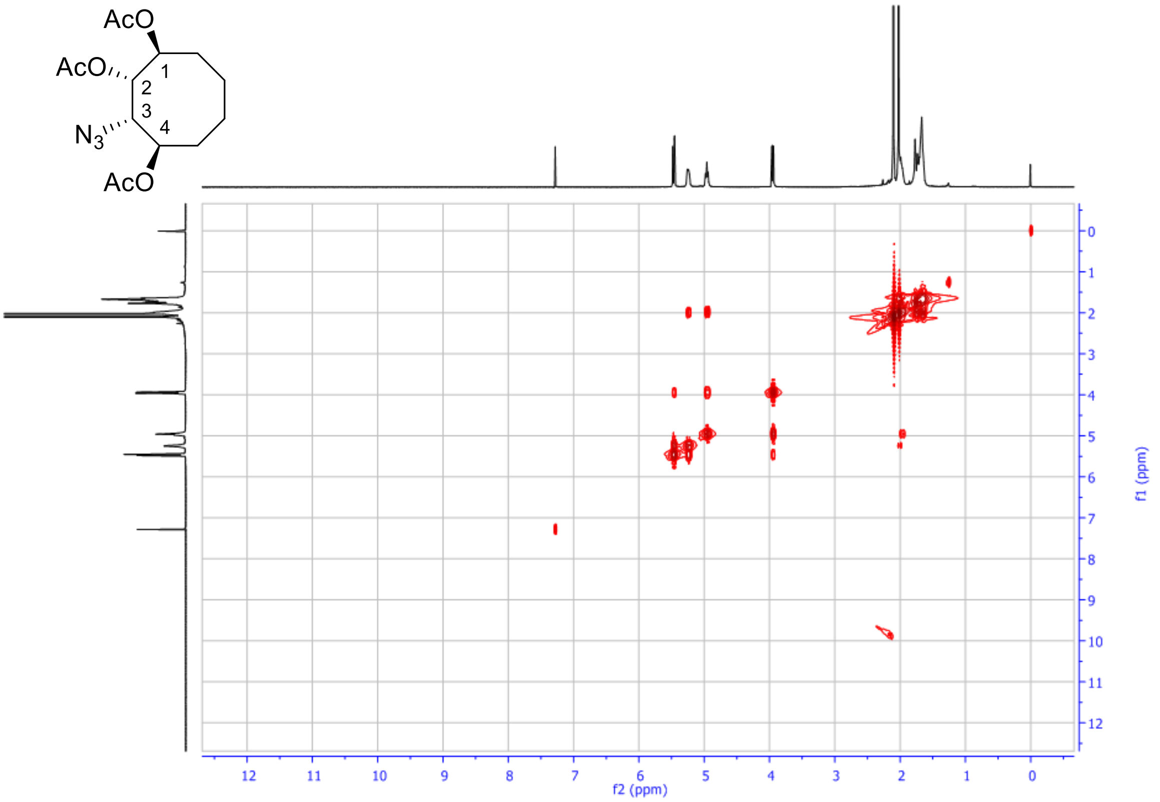

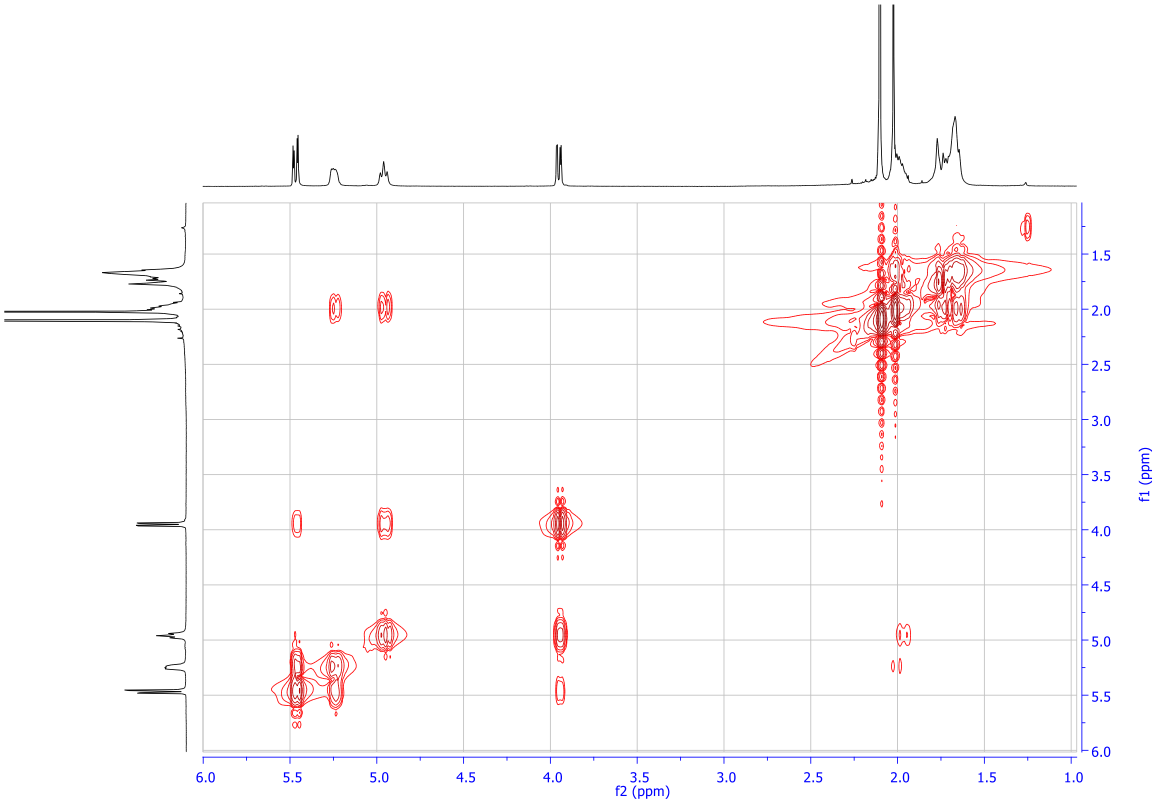

(1*S*(*R*),2*S*(*R*),3*S*(*R*),4*R*(*S*))-3-Aminocyclooctane-1,2,4-triol (18): D_2O (^{1}H NMR and ^{13}C NMR)

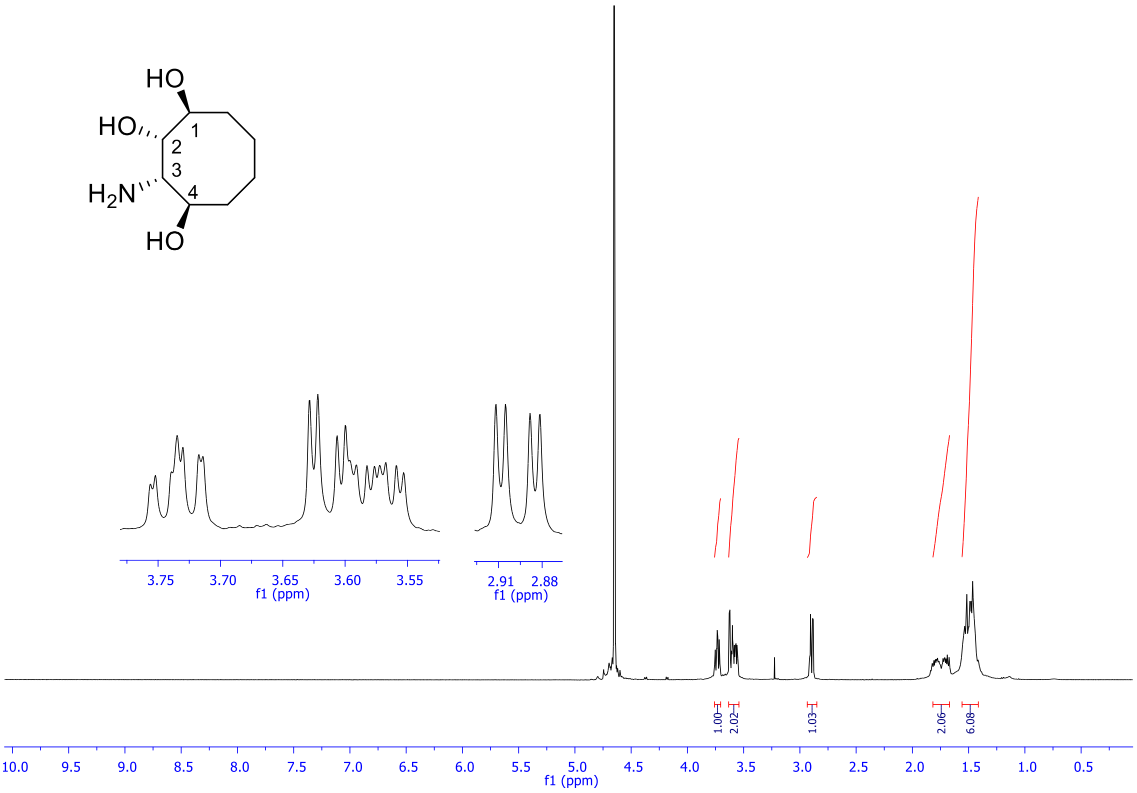

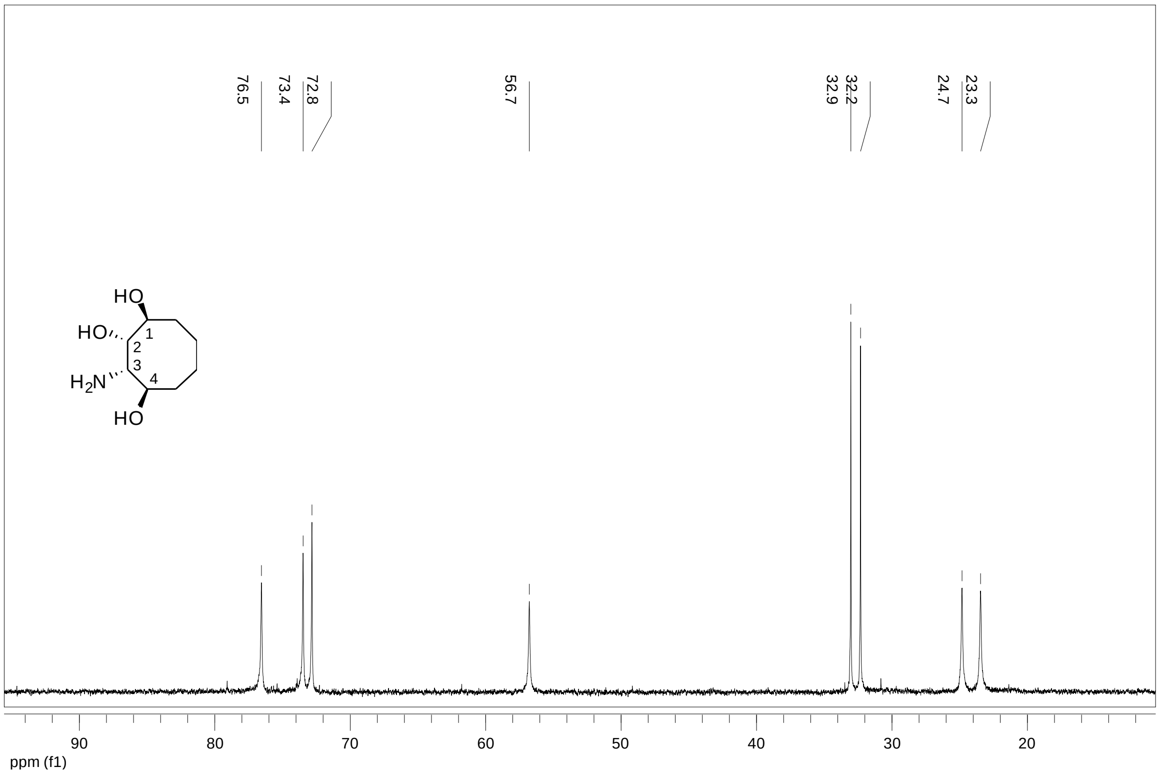

(1*S*(*R*),2*R*(*S*),3*R*(*S*),4*R*(*S*))-3-Chlorocyclooctane-1,2,4-triol (19): D_2O (^1H NMR and ^{13}C NMR)

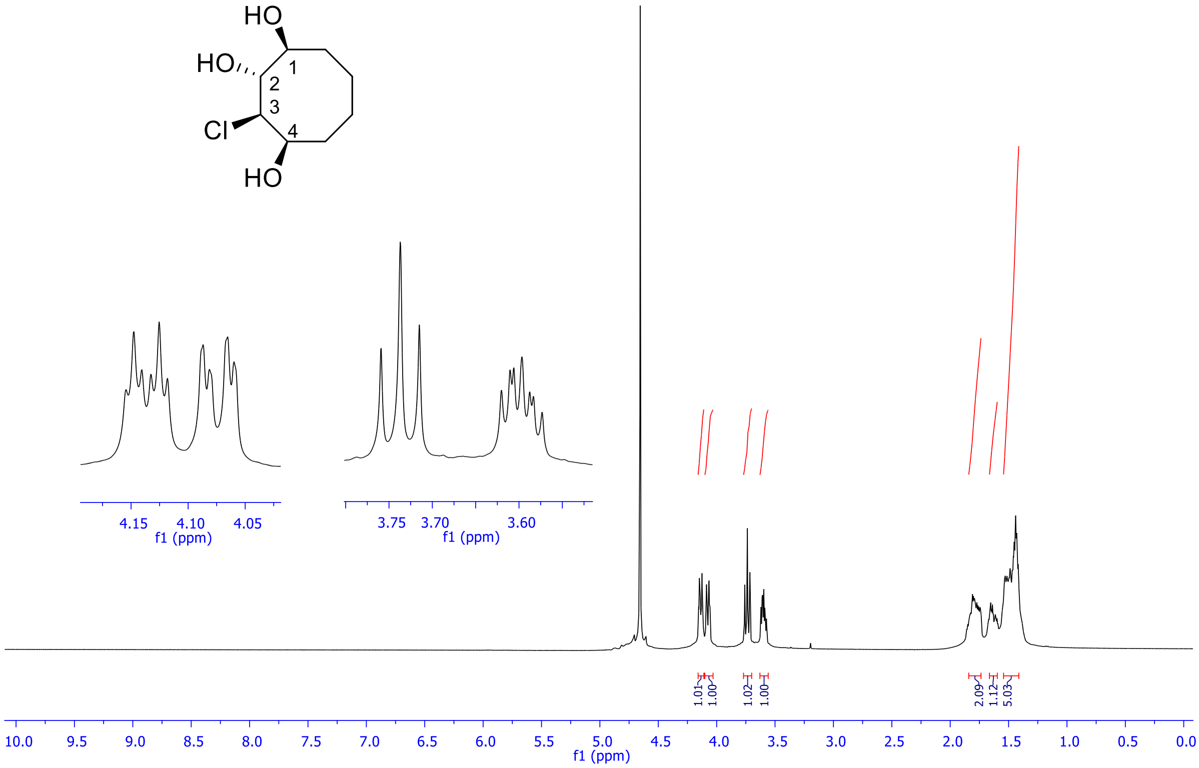

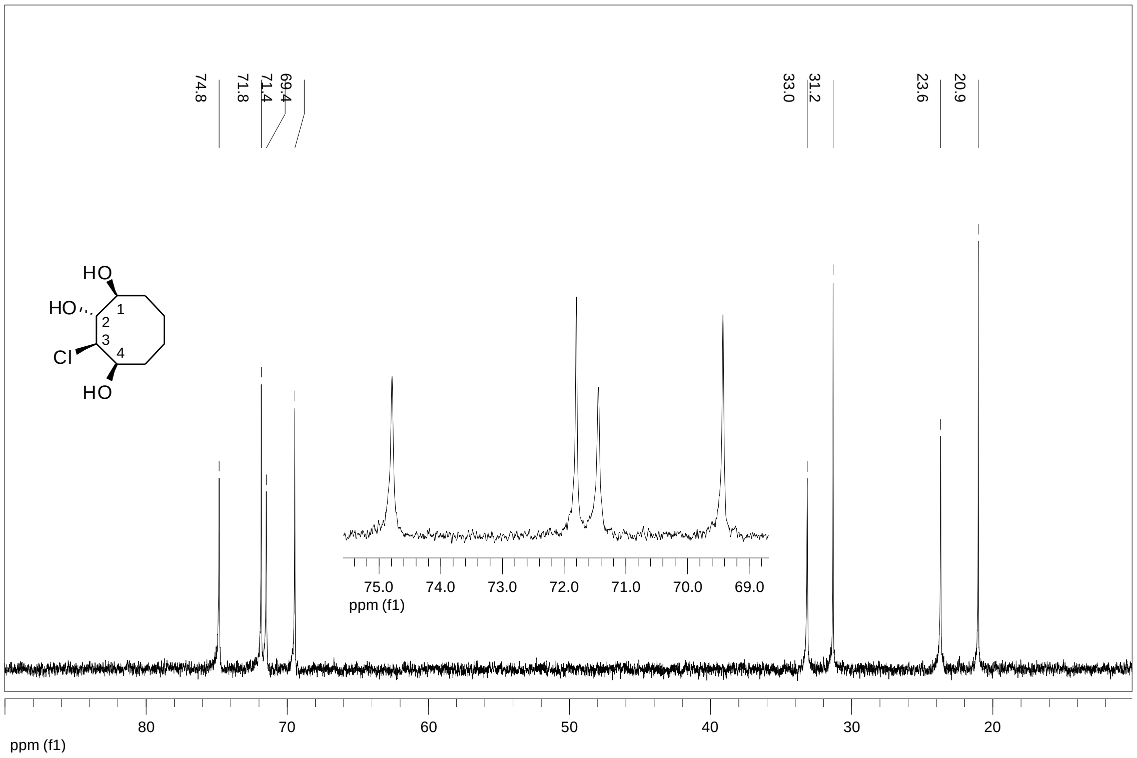

(1*S*(*R*),2*R*(*S*),3*R*(*S*),4*R*(*S*))-3-Chlorocyclooctane-1,2,4-triyl triacetate (20): $CDCl_3$ (^{1}H NMR and double resonance)

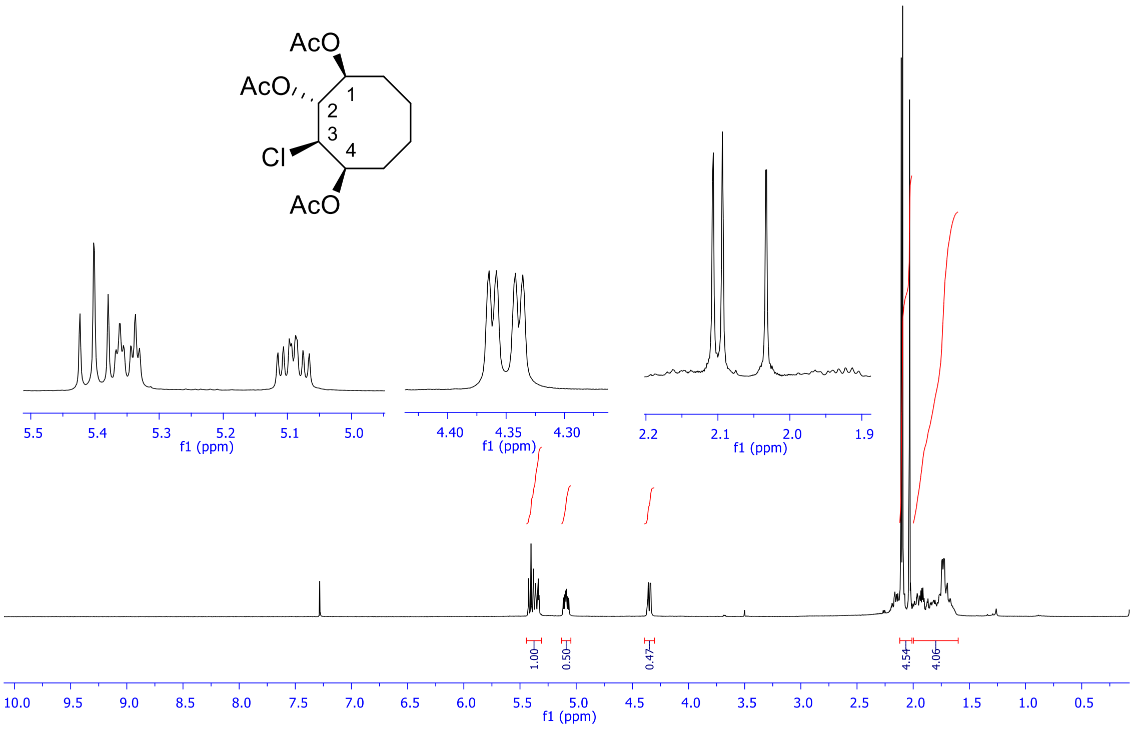

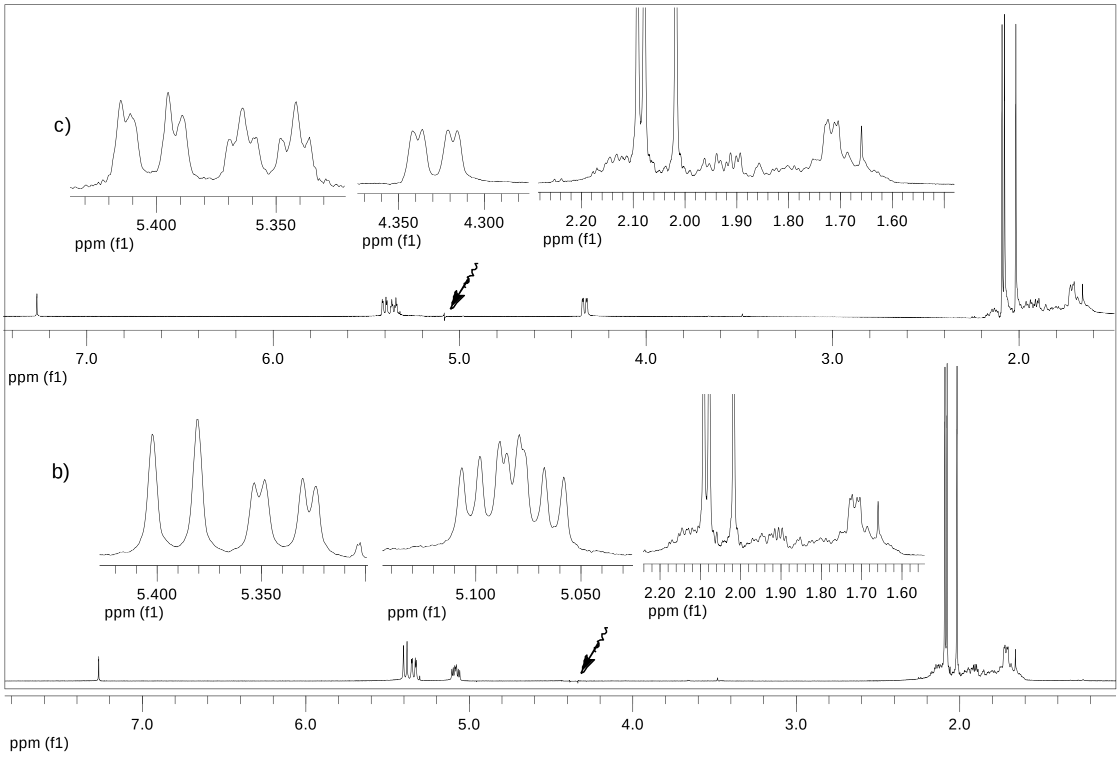

(1*S*(*R*),2*R*(*S*),3*R*(*S*),4*R*(*S*))-3-Chlorocyclooctane-1,2,4-triyl triacetate (20): $CDCl_3$ (^{13}C NMR)

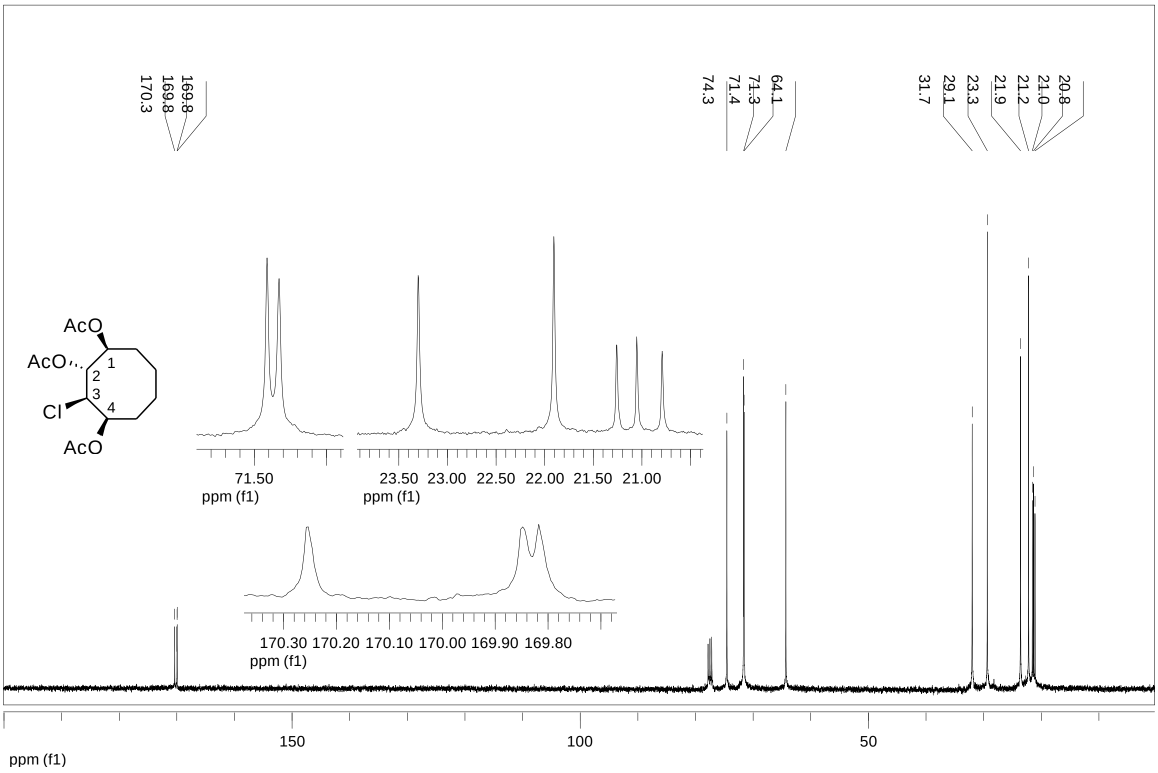

(1*S*(*R*),2*R*(*S*),3*R*(*S*),4*R*(*S*))-3-Chlorocyclooctane-1,2,4-triyl triacetate (20): $CDCl_3$-HMQC

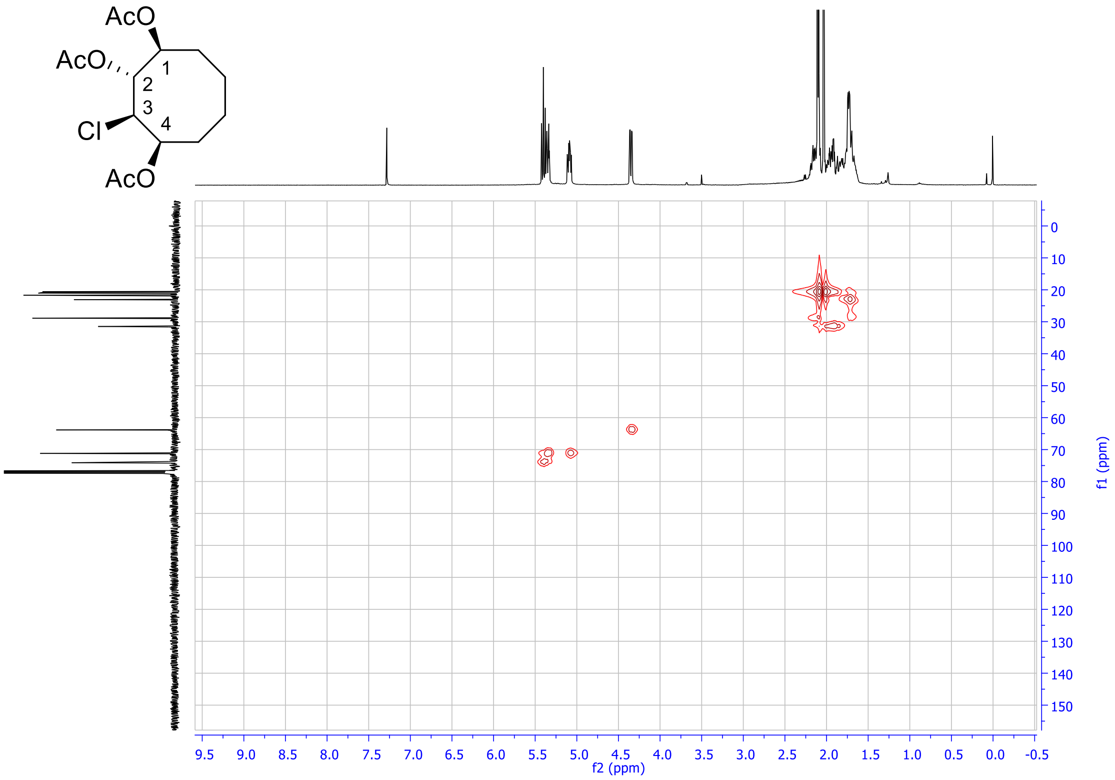

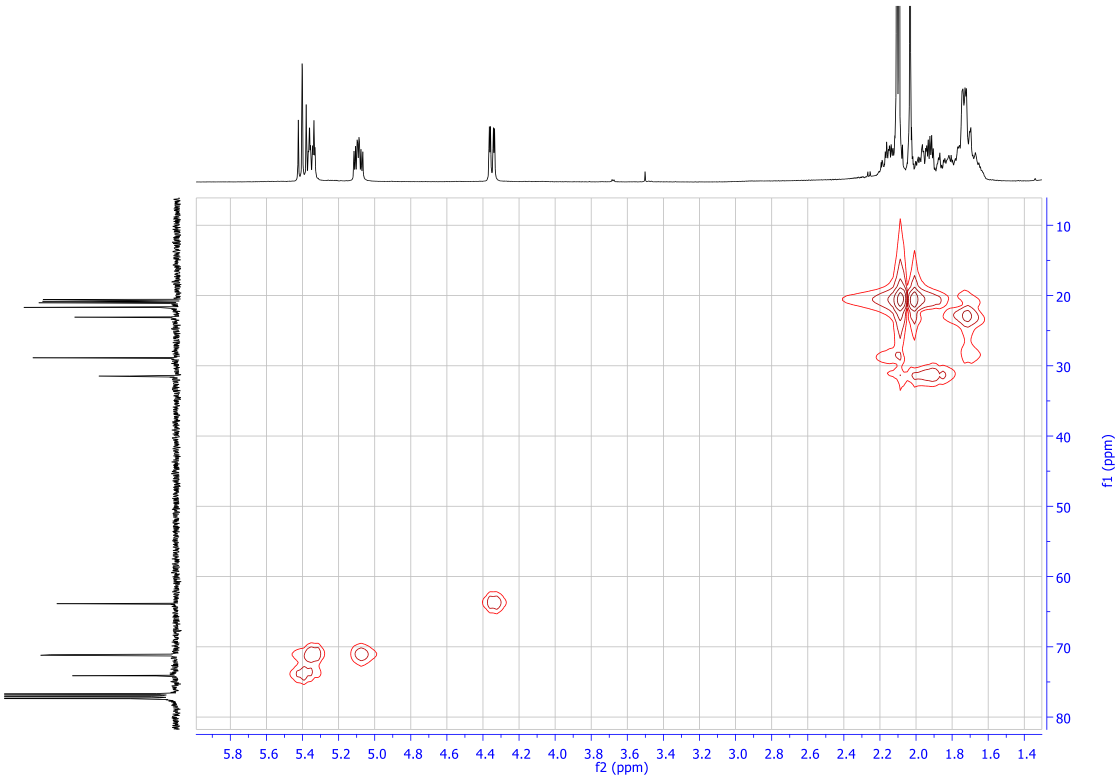

1*S*(*R*),2*R*(*S*),3*R*(*S*),4*R*(*S*))-3-Chlorocyclooctane-1,2,4-triyl triacetate (20): $CDCl_3$-COSY

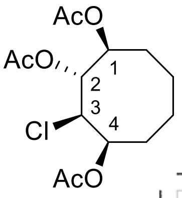

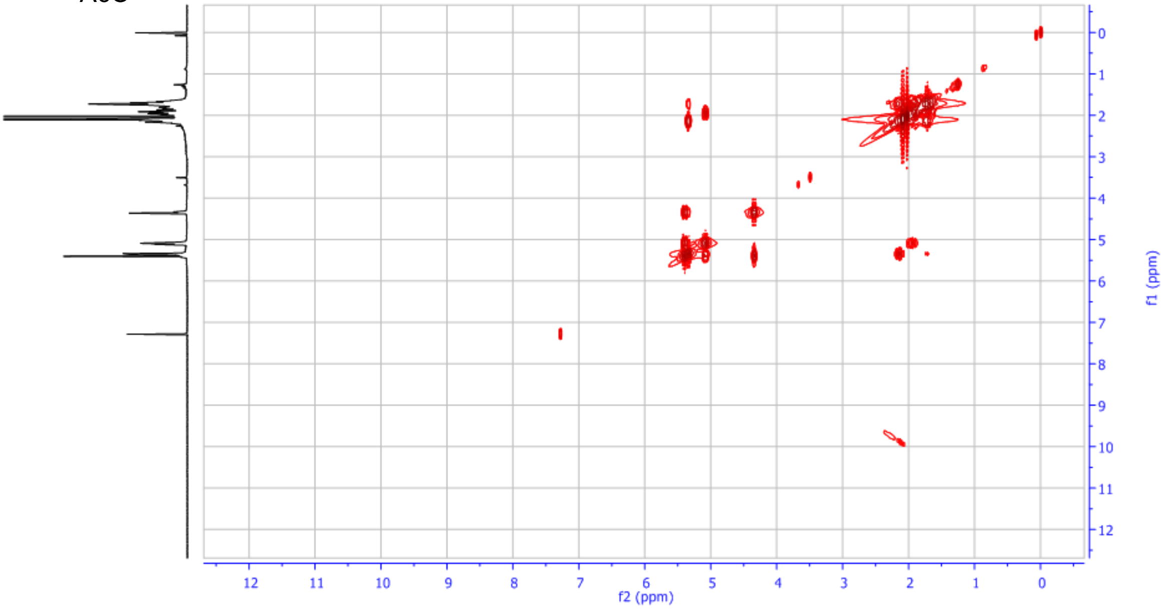

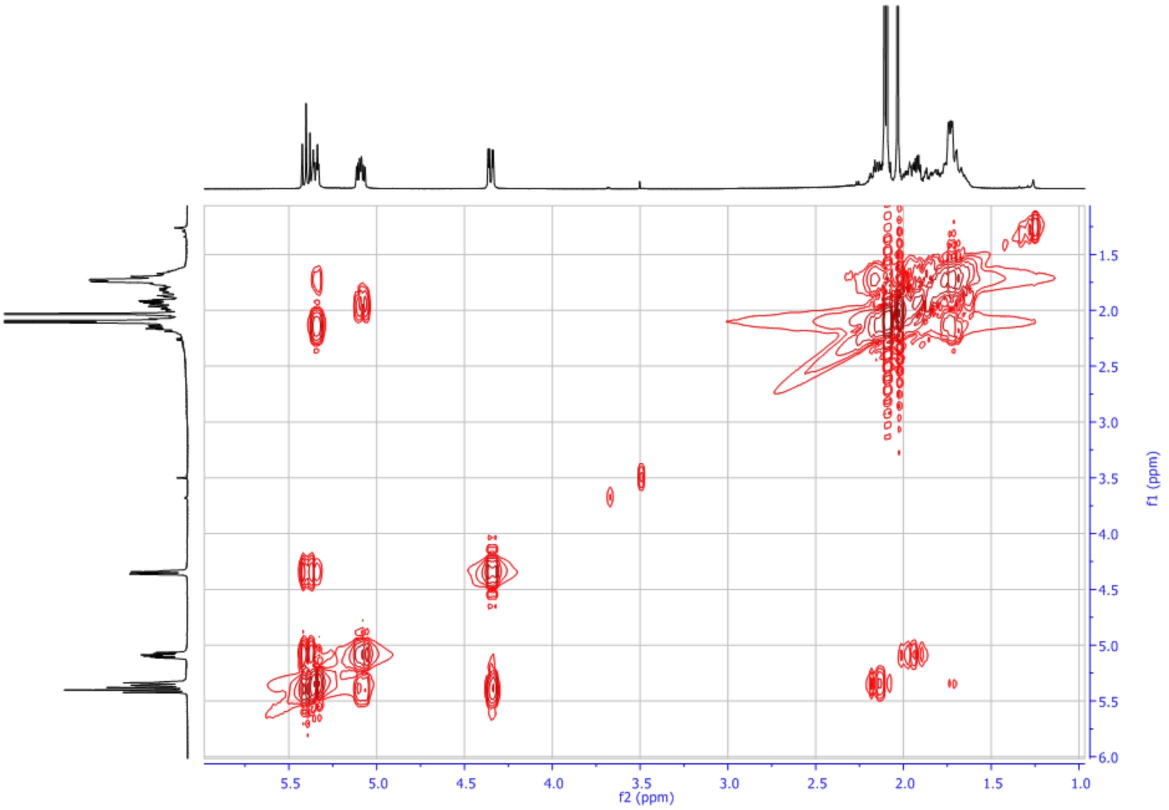

(1*R*(*S*),2*R*(*S*),3*S*(*R*),4*S*(*R*))-4-Chlorocyclooctane-1,2,3-triol (23): D_2O (^{1}H NMR and ^{13}C NMR)

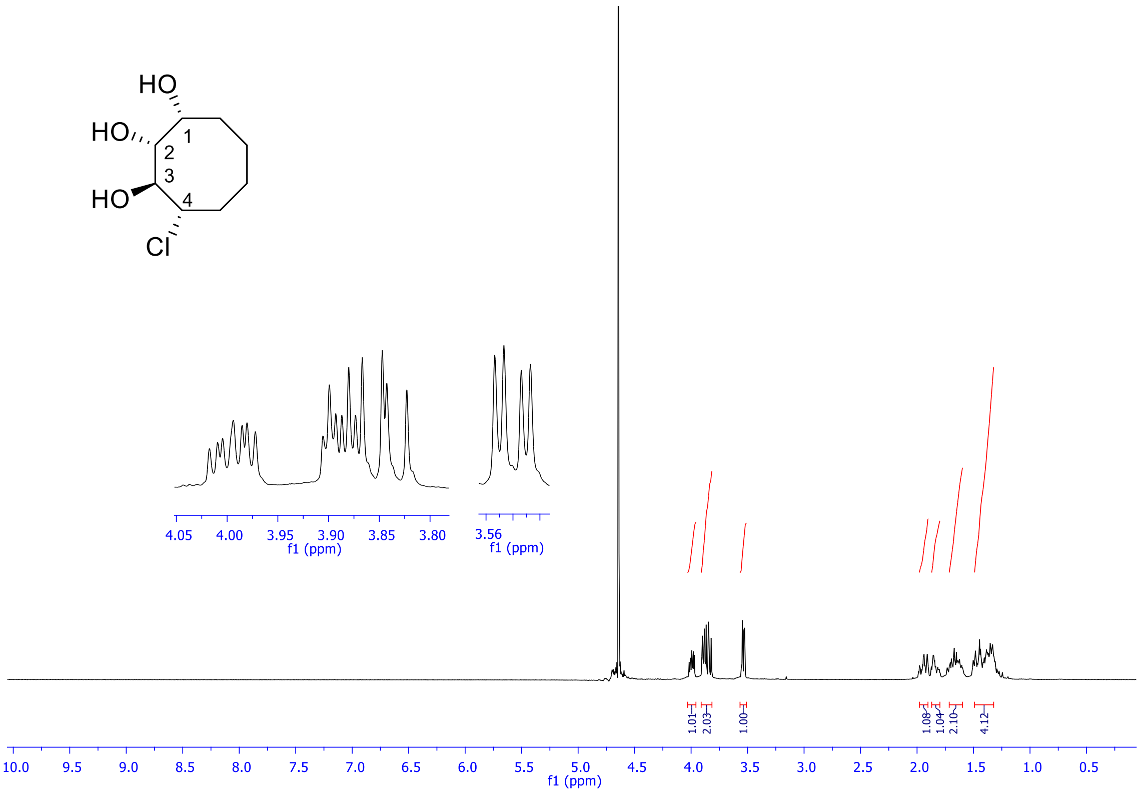

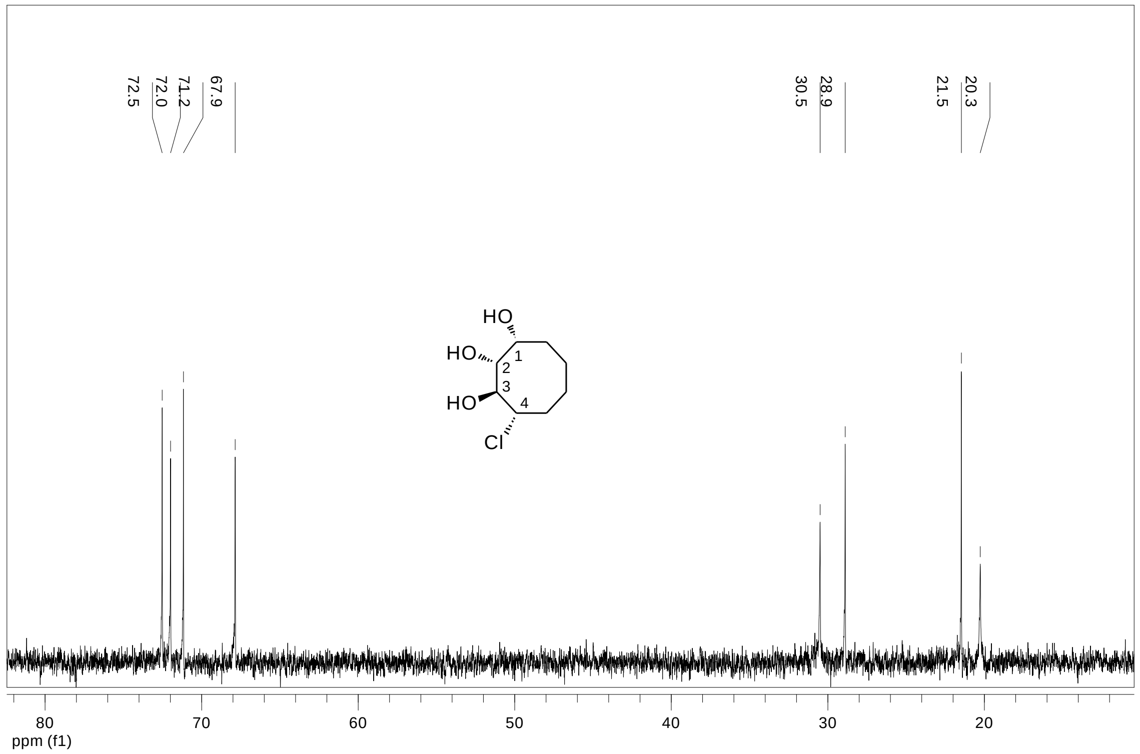

(1*R*(*S*),2*R*(*S*),3*S*(*R*),4*R*(*S*))-3-Chlorocyclooctane-1,2,4-triol (24): D$_2$O (^{1}H NMR and ^{13}C NMR)

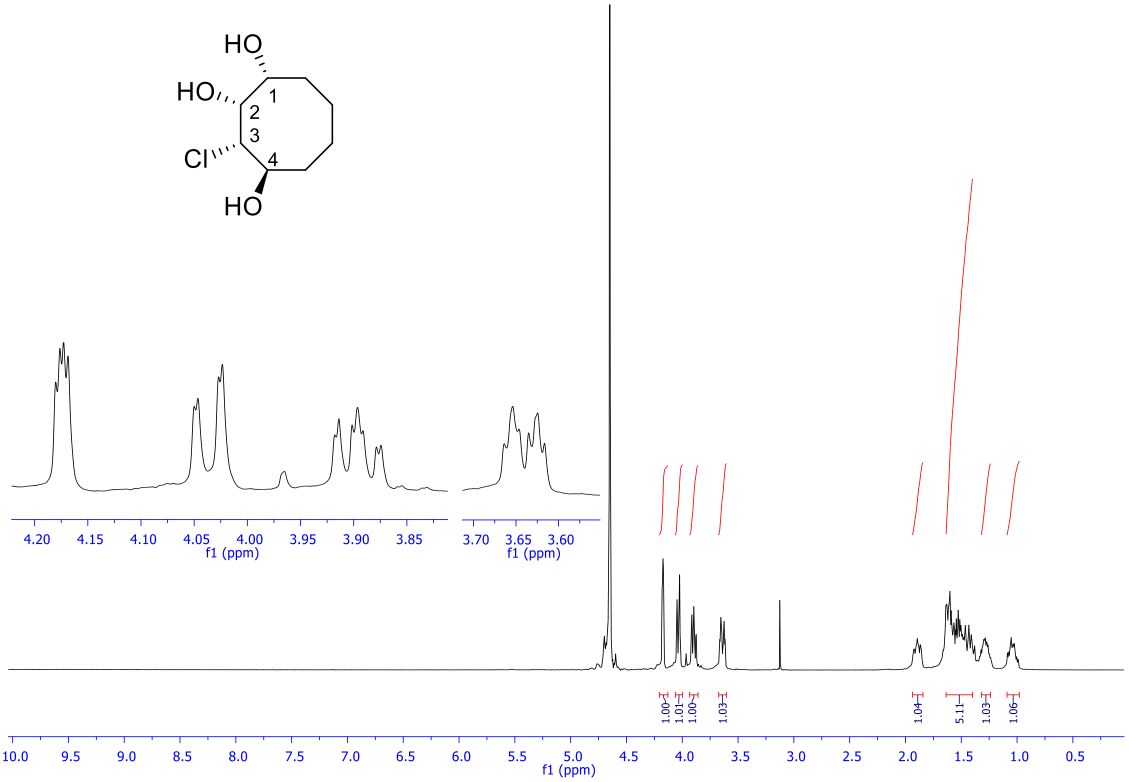

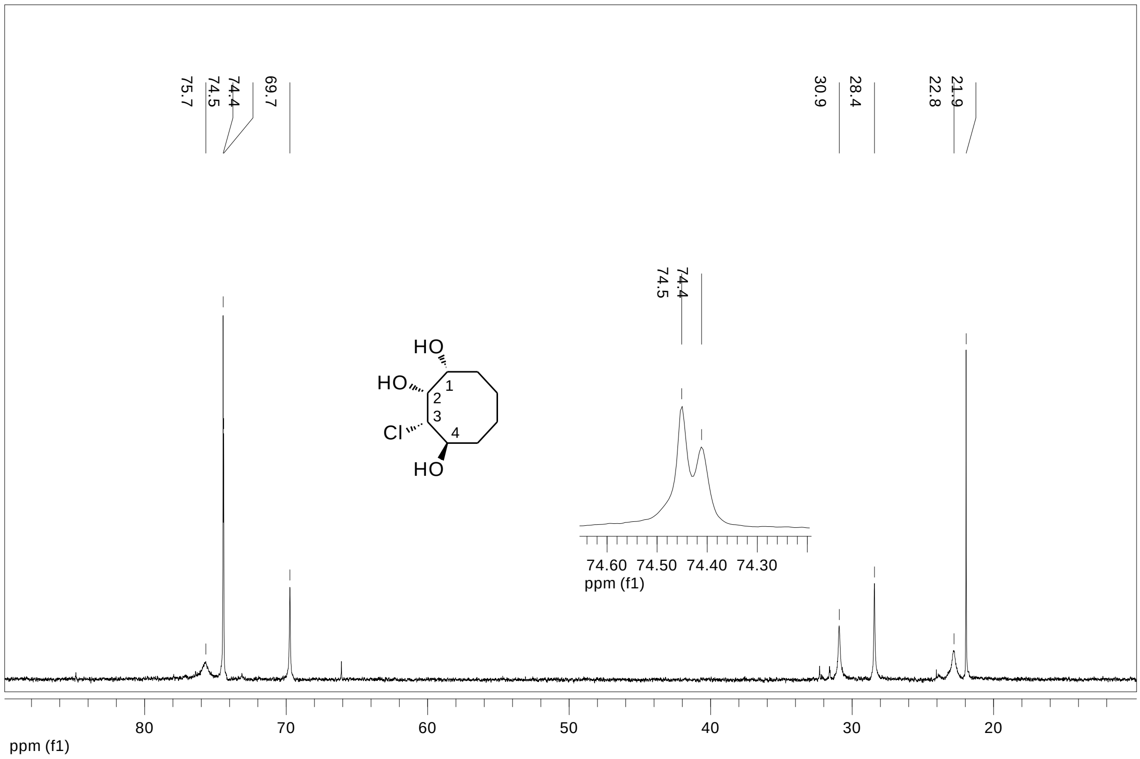

(1*R*(*S*),2*R*(*S*),3*S*(*R*),4*S*(*R*))-4-Chlorocyclooctane-1,2,3-triyl triacetate (25): $CDCl_3$ (^1H NMR and ^{13}C NMR)

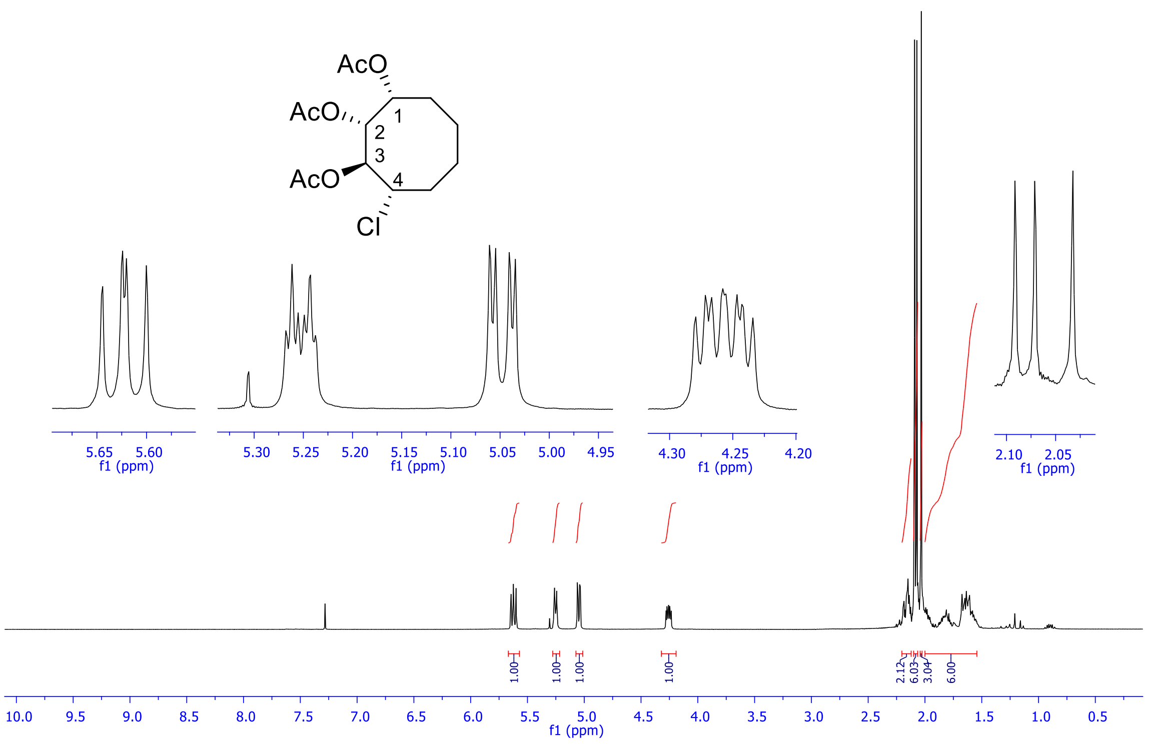

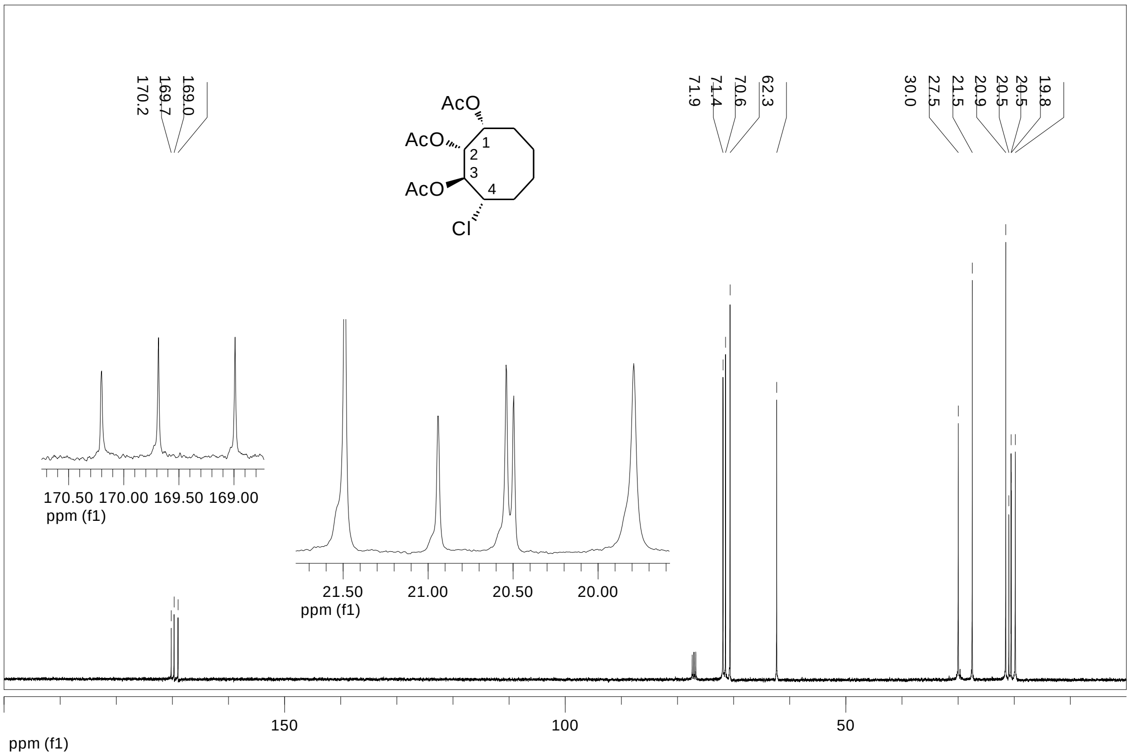

1*R*(*S*),2*R*(*S*),3*S*(*R*),4*S*(*R*))-4-Chlorocyclooctane-1,2,3-triyl triacetate (25): CDCl$_3$-HMQC

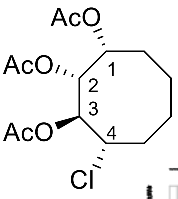

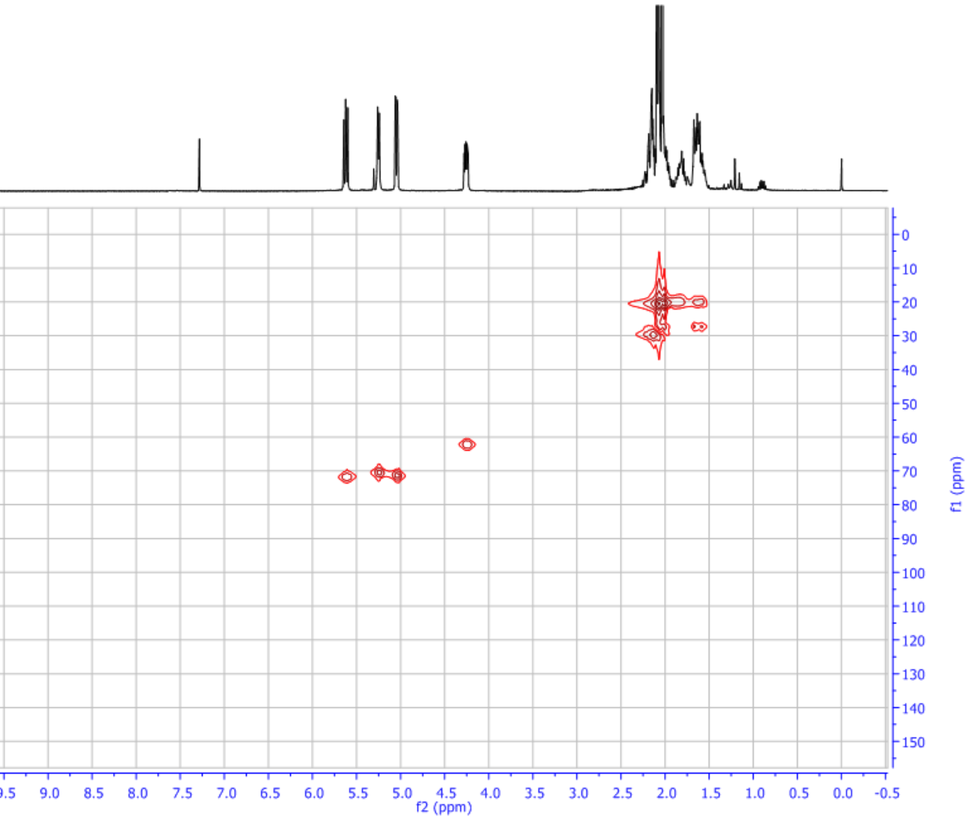

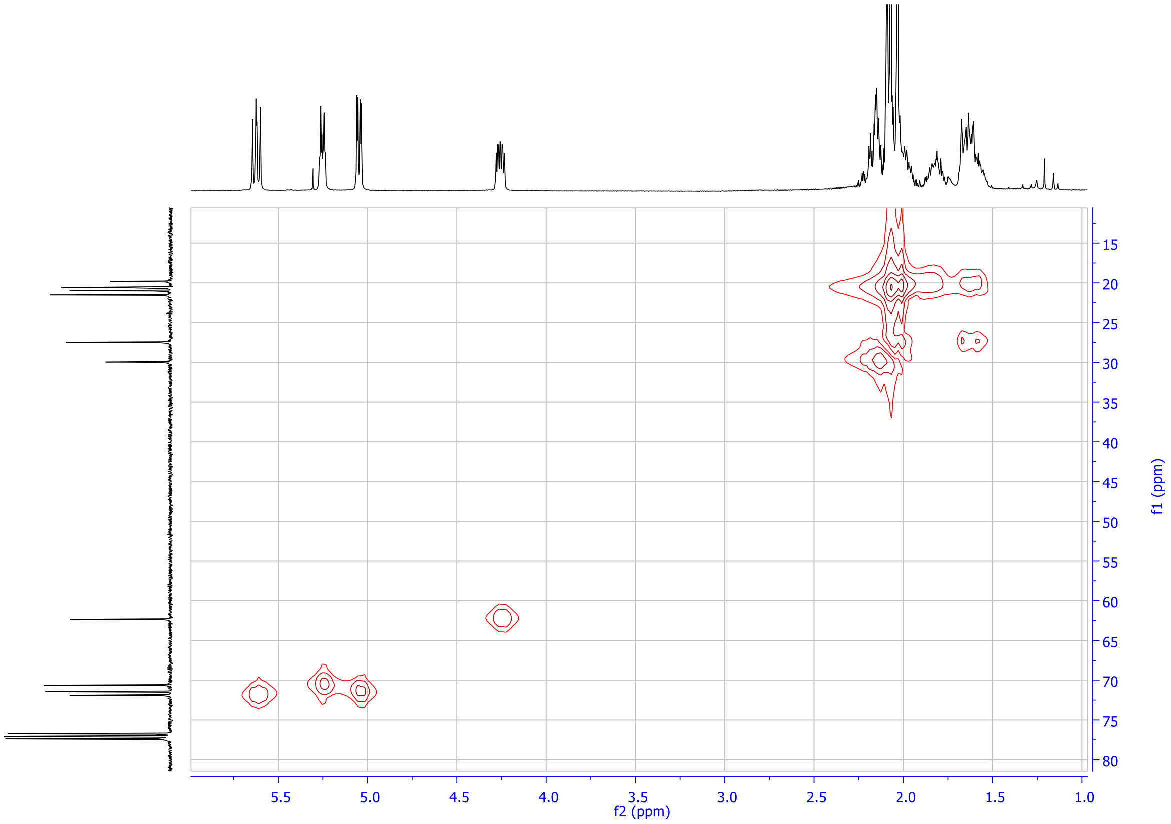

(1*R*(*S*),2*R*(*S*),3*S*(*R*),4*S*(*R*))-4-Chlorocyclooctane-1,2,3-triyl triacetate (25): $CDCl_3$-COSY

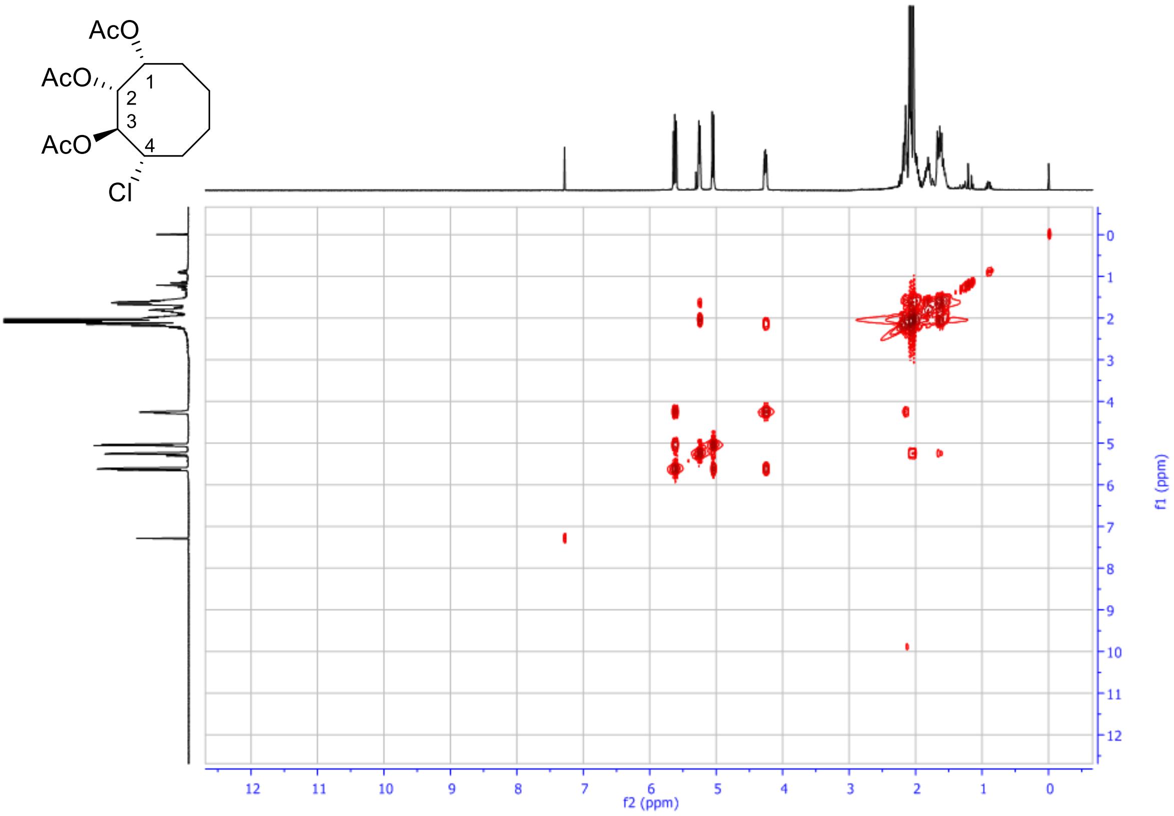

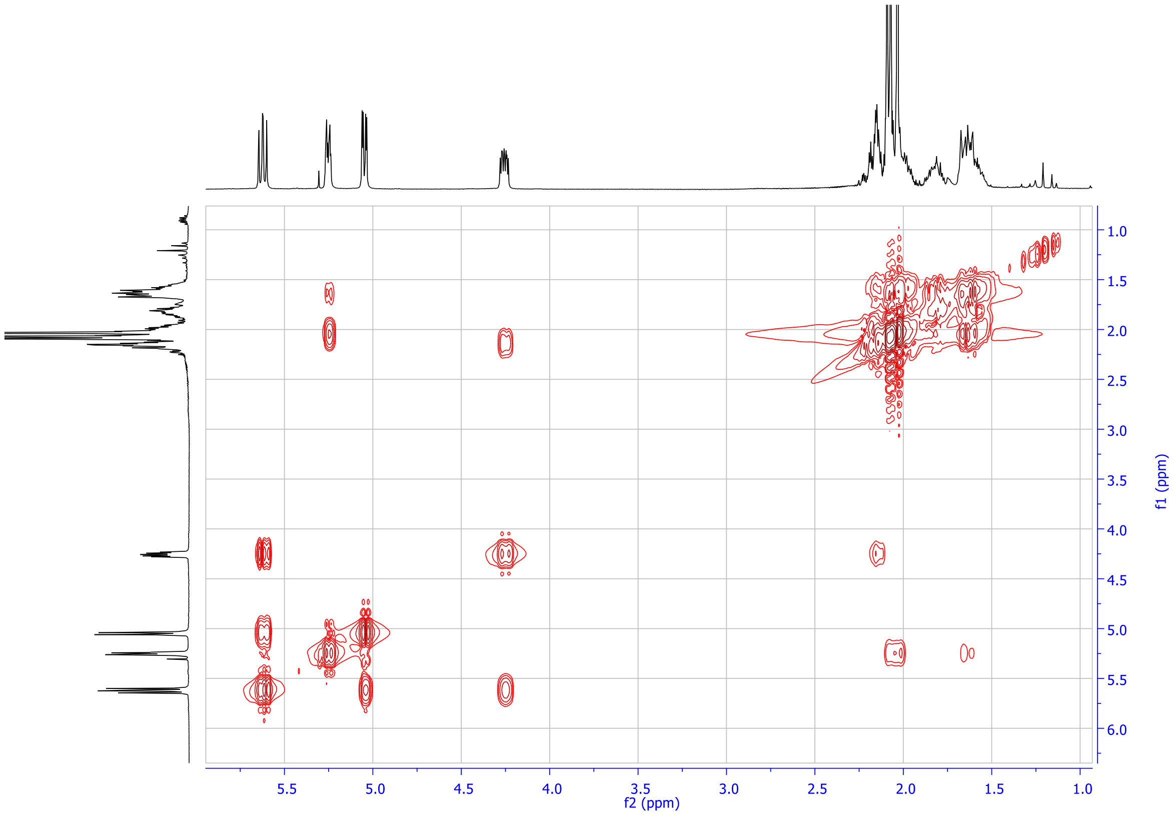

(1*R*(*S*),2*R*(*S*),3*S*(*R*),4*R*(*S*))-3-Chlorocyclooctane-1,2,4-triyl triacetate (26): $CDCl_3$ (^1H NMR and ^{13}C NMR)

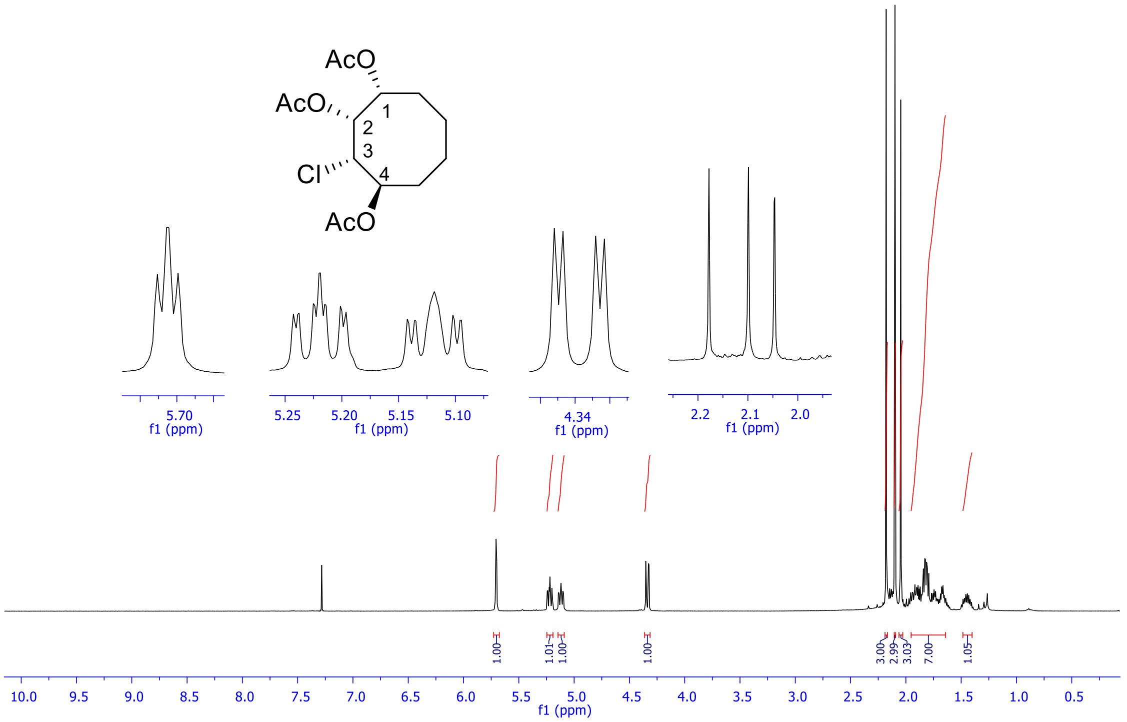

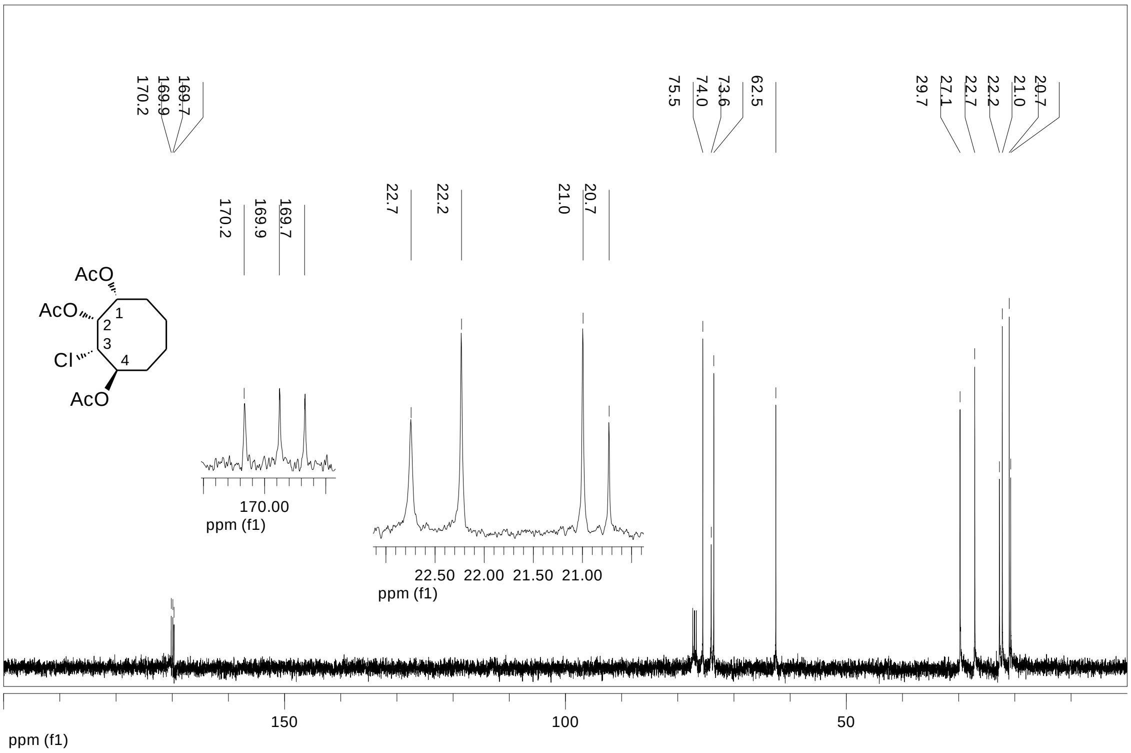

(1*R*(*S*),2*R*(*S*),3*S*(*R*),4*R*(*S*))-3-Chlorocyclooctane-1,2,4-triyl triacetate (26): CDCl$_3$-HMQC

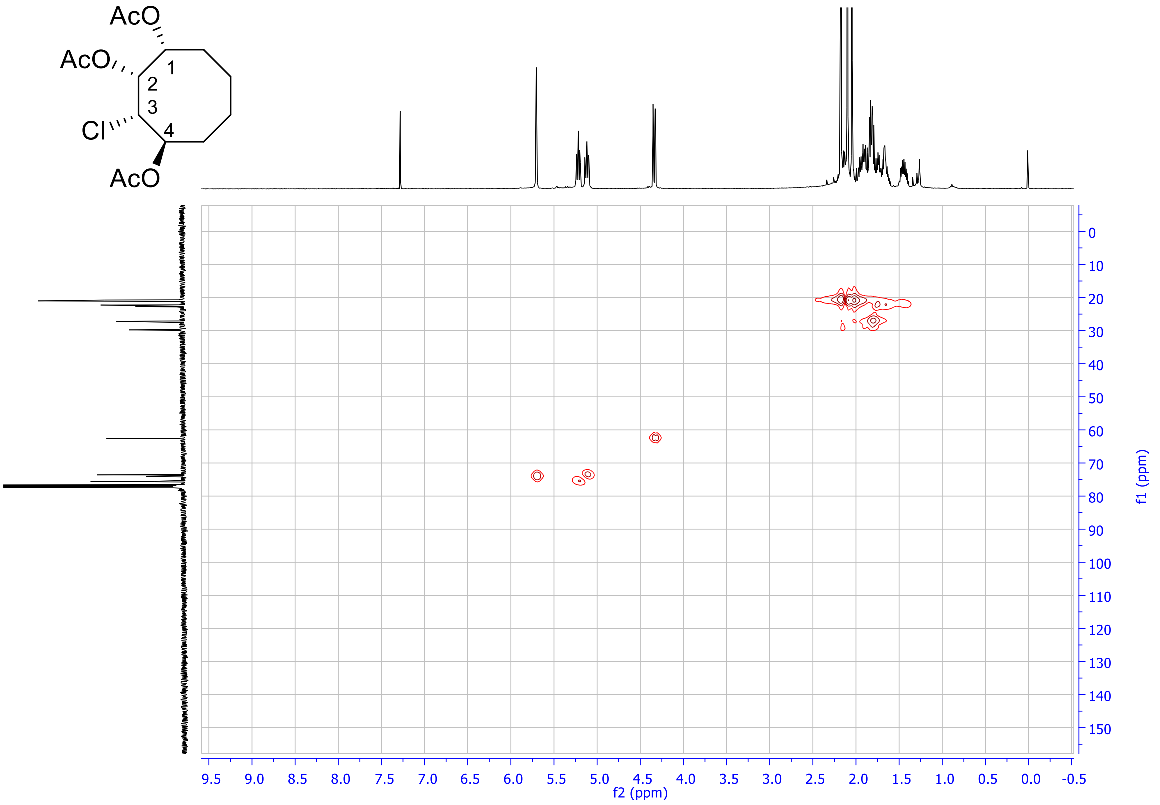

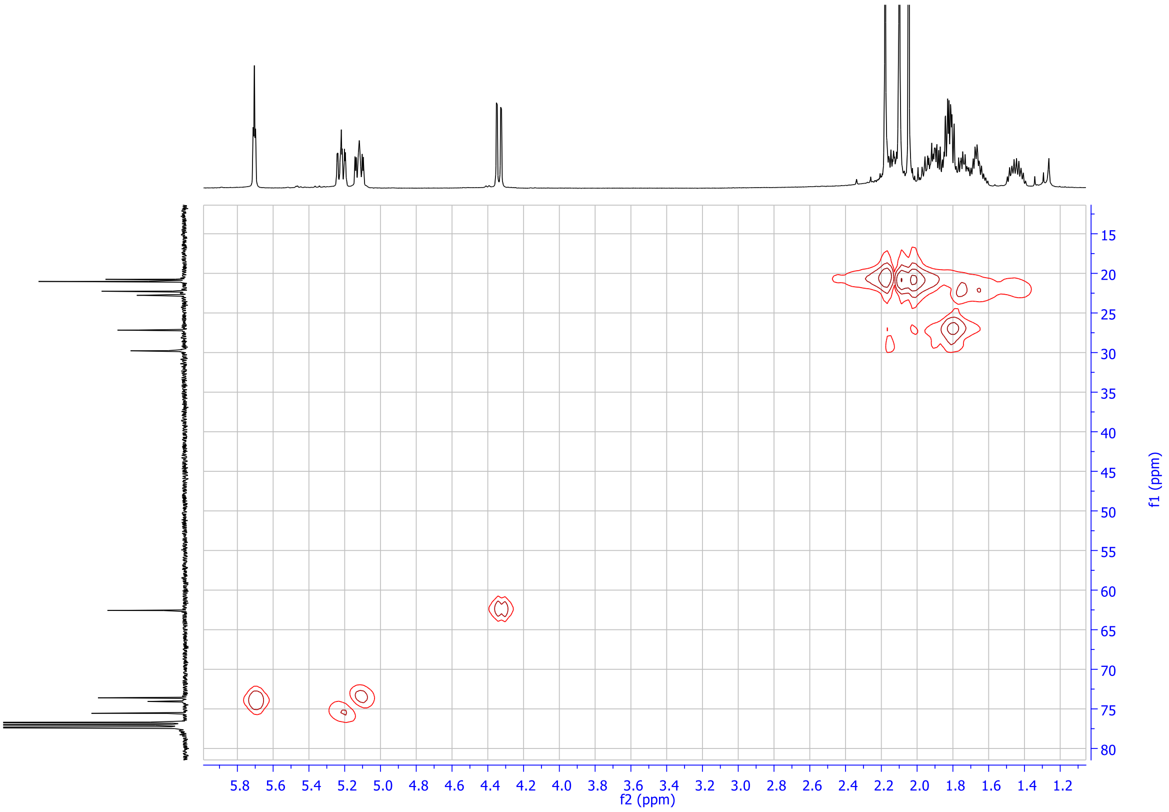

(1*R*(*S*),2*R*(*S*),3*S*(*R*),4*R*(*S*))-3-Chlorocyclooctane-1,2,4-triyl triacetate (26): $CDCl_3$-COSY

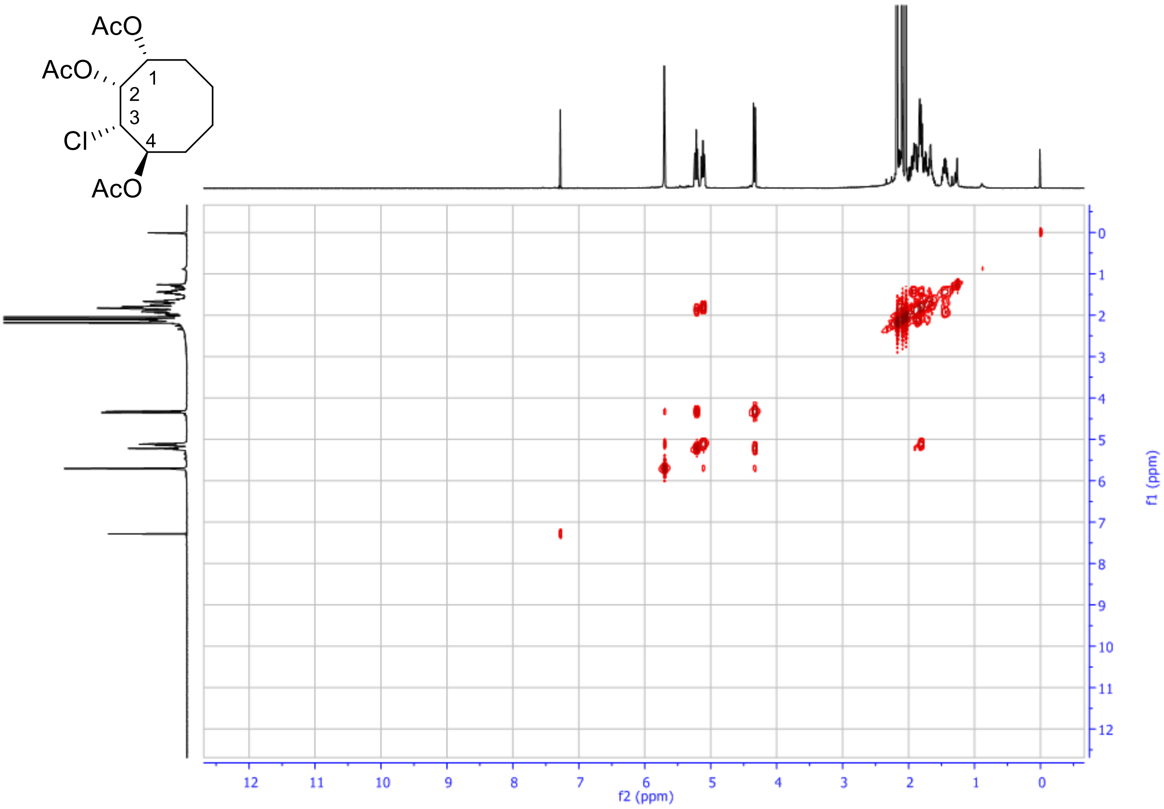

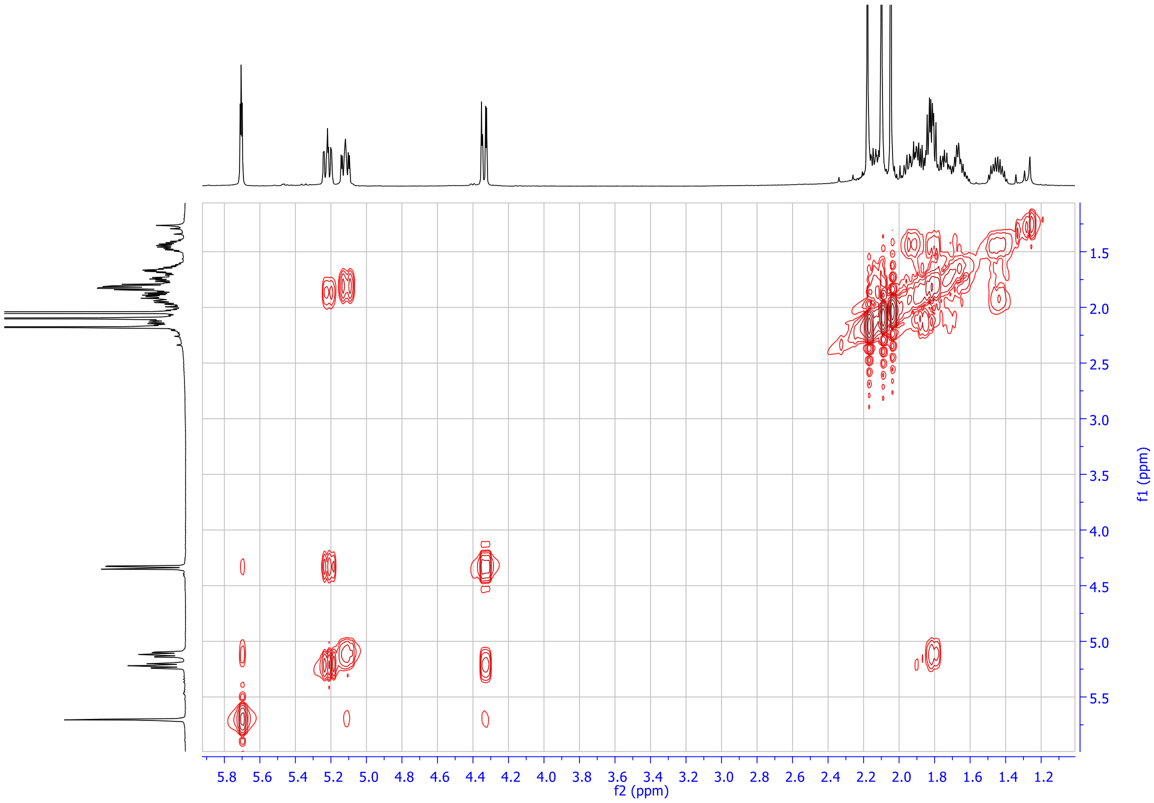

Supplement: File 1 — Experimental section, 1H and 13C NMR spectra for all new compounds, as well as selected 2D NMR spectra. [file Beilstein_J_Org_Chem-17-705-s001.pdf]
